# Rifamycin W Analogues from *Amycolatopsis mediterranei* S699 Δ*rif*-*orf5* Strain

**DOI:** 10.3390/biom11070920

**Published:** 2021-06-22

**Authors:** Yanrong Shi, Feng Ye, Yuliang Song, Xiaochun Zhang, Chunhua Lu, Yuemao Shen

**Affiliations:** 1 Key Laboratory of Chemical Biology (Ministry of Education), School of Pharmaceutical Sciences, Cheeloo College of Medicine, Shandong University, Jinan 250012, China; yrshi910212@sdu.edu.cn (Y.S.); yefeng1997@mail.sdu.edu.cn (F.Y.); 201936124@mail.sdu.edu.cn (Y.S.); 201816082@mail.sdu.edu.cn (X.Z.); ahua0966@sdu.edu.cn (C.L.); 2 Key Laboratory Experimental Teratology of the Ministry of Education, Department of Physiology, School of Basic Medical Sciences, Cheeloo College of Medicine, Shandong University, Jinan 250012, China

**Keywords:** *Amycolatopsis mediterranei* S699, rifamycin W, polyketide backbone rearrangement, oxidative cleavage

## Abstract

Rifamycin W, the most predominant intermediate in the biosynthesis of rifamycin, needs to undergo polyketide backbone rearrangement to produce rifamycin B via an oxidative cleavage of the C-12/C-29 double bond. However, the mechanism of this putative oxidative cleavage has not been characterized yet. Rif-Orf5 (a putative cytochrome P450 monooxygenase) was proposed to be involved in the cleavage of this olefinic moiety of rifamycin W. In this study, the mutant strain *Amycolatopsis mediterranei* S699 Δ*rif-orf5* was constructed by in-frame deleting the *rif*-*orf5* gene to afford thirteen rifamycin W congeners (**1**–**13**) including seven new ones (**1**–**7**). Their structures were elucidated by extensive analysis of 1D and 2D NMR spectroscopic data and high-resolution ESI mass spectra. Presumably, compounds **1**–**4** were derivatized from rifamycin W via C-5/C-11 retro-Claisen cleavage, and compounds **1**–**3**, **9** and **10** featured a hemiacetal. Compounds **5**–**7** and **11** showed oxygenations at various sites of the *ansa* chain. In addition, compounds **1**–**3** exhibited antibacterial activity against *Staphylococcus aureus* with minimal inhibitory concentration (MIC) values of 5, 40 and 0.5 µg/mL, respectively. Compounds **1** and **3** showed modest antiproliferative activity against HeLa and Caco-2 cells with half maximal inhibitory concentration (IC_50_) values of about 50 µM.

## 1. Introduction

Ansamycins are a family of macrolactam antibiotics that are synthesized by type I polyketide synthase (PKS), which are structurally characterized by an aromatic moiety bridged at nonadjacent positions by an aliphatic chain (*ansa* chain) [1,2]. As the representative members of the ansamycin family, rifamycins were first isolated from *Amycolatopsis mediterranei* S699 in 1957 [3,4,5]. Semi-synthetic rifamycin derivatives, such as rifampicin, rifapentine and rifambutin, have long been the first-line antituberculosis drugs since the mid-1960s, and are effective in combating leprosy and tuberculosis involved in AIDS-related mycobacterial infections [6,7,8,9]. However, *Mycobacterium tuberculosis* has developed significantly increased resistance to rifamycin antibiotics due to their extensive clinical use during recent decades [10,11].

The biosynthesis of rifamycins has been continuously studied since the 1980s, which can be divided into three stages. During the first two stages, the biosynthesis of start unit 3-amino-5-hydroxybenzoic acid (AHBA) and the polyketide skeleton were investigated, respectively. The third stage is still in progress, involving exploring hypotheses concerning post-PKS modifications [12,13,14,15,16]. As the most predominant intermediate in rifamycin biosynthesis, rifamycin W must undergo C-12/C-29 double bond oxidative cleavage to form 27-*O*-demethyl-25-*O*-deacetyl-rifamycin S (DMDARS) that is the basic rifamycin B polyketide skeleton. However, the mechanism of this putative oxidative cleavage has not been characterized yet [16,17].

**Figure 1:**
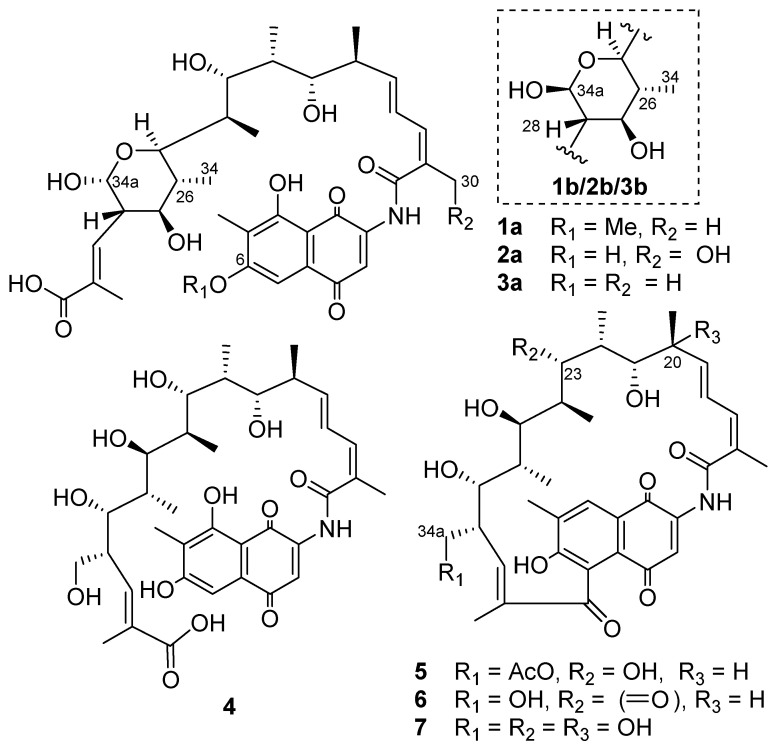
Structures of rifamycin W congeners **1**–**7**.

In this study, a candidate cytochrome P450 oxidase gene, *rif-orf5*, which may be responsible for the oxidative cleavage of the rifamycin W *ansa* chain, was inactivated in *A. mediterranei* S699. The mutant strain *A. mediterranei* S699 Δ*rif*-*orf5* cultivated for 7 d on YMG agar medium resulted in the isolation of thirteen rifamycin W congeners (**1**–**13**) including seven new ones (**1**–**7**) (Figure 1).

## 2. Materials and Methods

### 2.1. Bacterial Strains, Plasmids and Culture Media

The *Amycolatopsis mediterranei* S699 strain, isolated in 1957 in St. Raphael, France [5], was stored in our lab. The *A. mediterranei* S699 Δ*rif-orf5* strain was constructed by deleting the *rif-orf5* gene through homologous recombination. The *A. mediterranei* S699 Δ*rif-orf5*::*orf5* strain was constructed by transformation of the *rif-orf5* gene into the Δ*rif*-*orf5* mutant through electroporation. These strains were grown on YMG (yeast extract 4 g, malt extract 10 g, glucose 4 g, 20 g agar, ddH_2_O 1000 mL, pH 7.2) agar media at 28 °C for the production of rifamycins.

The *Escherichia* *coli* DH5α strain was used for plasmid propagation. Suicide vector pOJ260 was used for gene knock-out. Integrating vector pSET152 was used for gene complementation [18]. *E*. *coli* strains were maintained in LB (tryptone 10 g, yeast extract 5 g, NaCl 10 g, ddH_2_O 1000 mL, pH 7.2) media at 37 °C. Apramycin was added into media at a final concentration of 50 μg∙mL^−1^. Cells were stocked with 20% glycerol and stored at −80 °C.

### 2.2. Molecular Cloning and Mutant Construction

#### 2.2.1. Construction of the *rif*-*orf5* Gene Knock-Out Mutant Δ*rif*-*orf5*

First, the *rif*-*orf5* gene knock-out vector pOJ260-orf5 was constructed. Two ca. 2 kb DNA fragments flanking upstream and downstream of the target gene were amplified from the genomic DNA of *A. mediterranei* S699, and named HF1 and HF2, respectively. The purified homologous fragments HF1 and HF2 were digested with HindIII/XbaI and XbaI/EcoRI, and cloned into linearized HindIII/EcoRI digested pOJ260. The ligation product was transformed into DH5α-competent cells. Positive clones were verified by restriction enzyme digestion and sequencing (Figures S1A and S2). The gene knock-out vector pOJ260-orf5 was introduced into the rifamycin-producing strain *A. mediterranei* S699 by electrotransformation [19]. Apramycin-resistant (AprR) colonies were selected and confirmed to be single cross-over mutants by PCR amplification (Figures S1B and S3A). Apramycin-sensitive (AprS) colonies were counterselected from the initial AprR single cross-over colonies after several rounds of nonselective growth, and confirmed to be double cross-over gene knock-out mutant Δ*rif*-*orf5* by PCR amplification (Figures S1B and S3B).

#### 2.2.2. Construction of the *rif*-*orf5* Gene Complementation Mutant Δ*rif*-*orf5*::*orf5*

First, the *rif*-*orf5* gene complementation vector pSET152-orf5 was constructed. The targeted gene *rif*-*orf5* was amplified using the genomic DNA of *A. mediterranei* S699 as a template. The purified PCR fragment was digested with NdeI and XbaI, and cloned into the downstream of the *rifK*p promoter in pSET152 through Gibson assembly [20]. Similarly, the assembled product was transformed into DH5α-competent cells, and positive clones were verified by restriction enzyme digestion and sequencing (Figure S4). The gene complementation vector pSET152-orf5 was transformed into the *rif*-*orf5* gene knock-out mutant Δ*rif*-*orf5* by electroporation. Apramycin-sensitive (AprS) colonies were selected and confirmed to be the *rif*-*orf5* gene complementation mutant Δ*rif*-*orf5*::*orf5* by PCR amplification (Figure S5). 

Primers used in this study are shown in Table S1.

### 2.3. HPLC Detection of the Metabolites in Mutants

*A. mediterranei* S699 mutants were inoculated on YMG agar media (100 mL) and cultivated for 7 days at 28 °C. The culture was diced and extracted overnight with EtOAc at room temperature. The concentrated crude extract was dissolved in 1 mL MeOH, and analyzed by high-pressure liquid chromatography (HPLC; Agilent 1200, Santa Clara, CA, USA) in a gradient system consisting of ddH_2_O + 0.5% formic acid as solvent A and acetonitrile as solvent B. The program of solvent gradient was as follows: 20–35% B in the first 5 min, 35–55% B from 5 to 19 min, 55–65% B from 19 to 23 min, 65–100% B from 23 to 27 min. Flow rate was 1 mL/min, and UV detection was monitored at 254 nm (Figure S6).

### 2.4. Extraction and Isolation of the Metabolites from the Δrif-orf5 Strain

#### 2.4.1. General Experimental Procedures

The nuclear magnetic resonance (NMR) spectra were recorded on Bruker 400 MHz NMR spectrometer. HRESIMS analyses were carried out on an LTQ-Orbitrap XL (Thermo Scientific, Waltham, MA, USA). HPLC was performed on an Agilent 1200. Semi-preparative HPLC was performed on a Waters 1525 Binary HPLC Pump (Agilent Eclipse XDB-C_18_, 5 µm, 9.4 × 250 mm) with a Waters 996 Photodiode Array Detector (Milford, MA, USA). Sephadex LH-20 was obtained from GE Amersham Biosciences (Piscataway, NJ, USA). Column chromatography (CC) was performed over reversed-phase (RP) C_18_ silica gel (Merck, Darmstadt, Germany). Silica gel GF_254_ for thin-layer chromatography (TLC) was purchased from Qingdao Marine Chemical Ltd. (Qingdao, China). Optical rotations were measured on an Auton Paar MCP200 Automatic Polarimeter. IR spectra (KBr) were obtained on a Thermo Fisher Scientific Nicolet 6700 FT-IR spectrometer (Waltham, MA, USA). Compounds were visualized under UV light and by iodine vapor.

#### 2.4.2. Fermentation, Extraction and Isolation of the Metabolites from the Δ*rif*-*orf5* Strain

The fermentation (20 L) was performed on YMG agar Petri dishes for 7 d at 28 °C. The culture was diced and extracted overnight with EtOAc/MeOH (4:1, *v*/*v*) at room temperature three times. The crude extract was partitioned between H_2_O and EtOAc (1:1, *v*/*v*) until the H_2_O layer was colorless. The EtOAc extract was partitioned between 95% aqueous MeOH and petroleum ether (PE) to afford the defatted MeOH extract. The MeOH extract was fractionated by medium-pressure liquid chromatography (MPLC) over RP C_18_ silica gel (130 g) eluted with gradient aqueous CH_3_CN (30%, 50%, 70% and 100% CH_3_CN, 500 mL each) to give Fr. A–J.

Fr. C (1.43 g) was purified by HPLC (4 mL/min, UV 254 nm) eluted with 32% CH_3_CN to afford **7** (*t*_R_ 13.4 min, 17.5 mg) and **12** (*t*_R_ 6.2 min, 29.8 mg). Fr. D (5.62 g) was subjected to CC over silica gel (150 g) eluted with gradient CH_2_Cl_2_:MeOH (50:1, 30:1, 15:1 and 5:1, 500 mL each) to afford Fr. D1–4. Fr. D2 (1.85 g) was purified by HPLC (4 mL/min, UV 254 nm) eluted with 35% CH_3_CN to afford **10** (*t*_R_ 8.6 min, 33 mg) and **14** (*t*_R_ 10.3 min, 41 mg). Fr. D3 (2.49 g) was purified by HPLC (4 mL/min, UV 254 nm) eluted with 40% CH_3_CN to afford **6** (*t*_R_ 7.9 min, 9.9 mg), **8** (*t*_R_ 15.3 min, 17.0 mg) and **13** (*t*_R_ 11.4 min, 179.5 mg). Fr. E (1.74 g) was purified by HPLC (4 mL/min, UV 254 nm) eluted with 43% CH_3_CN to afford **2** (*t*_R_ 7.5 min, 10.5 mg), **4** (*t*_R_ 13.4 min, 6.8 mg) and **5** (*t*_R_ 6.2 min, 29.7 mg). Similarly, compounds **9** (12.3 mg) and **11** (7.2 mg) were obtained from Fr. F, and **3** (13.5 mg) was purified from Fr. G by HPLC (4 mL/min, UV 254 nm) eluted with 45% CH_3_CN. Fr. I gave compound **1** (6.6 mg) through HPLC (4 mL/min; UV 254 nm) eluted with 58% CH_3_CN.

Compound **1**: dark brown powder; [α]^20^_*D*_ = +10.0 (*c* 0.10, MeOH); UV (MeOH) *λ*_max_ (logε) 217 (4.40), 269 (4.30), 325 (4.00) nm; IR (KBr) *ν*_max_ 3369, 2963, 2925, 1688, 1631, 1496, 1322, 1142, 977, 862 cm^−1^; ^1^H NMR data, Table 1; ^13^C NMR data, Table 2; HRESIMS: *m*/*z* 686.3172 [M + H]^+^ (calcd for C_36_H_48_NO_12_^+^, 686.3171), and 708.2992 [M + Na]^+^ (calcd for C_36_H_47_NNaO_12_^+^, 708.2990).

Compound **2**: dark brown powder; [α]^20^_*D*_ = −4.0 (*c* 0.10, MeOH); UV (MeOH) *λ*_max_ (logε) 218 (4.38), 274 (4.27), 323 (3.99) nm; IR (KBr) *ν*_max_ 3359, 2972, 2931, 1687, 1629, 1503, 1326, 1122, 1047, 979, 875, 755 cm^−1^; ^1^H NMR data, Table 1; ^13^C NMR data, Table 2; HRESIMS: *m*/*z* 688.2956 [M + H]^+^ (calcd for C_35_H_46_NO_13_^+^, 688.2964) and 710.2781 [M + Na]^+^ (calad for C_35_H_45_NNaO_13_^+^, 710.2783).

Compound **3**: dark brown powder; [α]^20^_*D*_ = +12.0 (*c* 0.10, MeOH); UV (MeOH) *λ*_max_ (logε) 218 (4.12), 272 (3.94), 323 (3.66) nm; IR (KBr) *ν*_max_ 3366, 2972, 2932, 1598, 1498, 1377, 1325, 1121, 1069, 981, 758 cm^−1^; ^1^H NMR data, Table 1; ^13^C NMR data, Table 2; HRESIMS: *m*/*z* 672.3011 [M + H]^+^ (calcd for C_35_H_46_NO_12_^+^, 672.3015), and 694.2835 [M + Na]^+^ (calcd for C_35_H_45_NNaO_12_^+^, 694.2834).

Compound **4**: maroon powder; [α]^20^_*D*_ = +28.0 (*c* 0.10, MeOH); UV (MeOH) *λ*_max_ (logε) 214 (3.90), 273 (3.66), 323 (3.37) nm; IR (KBr) *ν*_max_ 3409, 2948, 2836, 1656, 1451, 1413, 1203, 1114, 1024, 695 cm^−1^; ^1^H NMR data, Table 3; ^13^C NMR data, Table 2; HRESIMS: *m*/*z* 674.3172 [M + H]^+^ (calcd for C_35_H_48_NO_12_^+^, 674.3171), and 696.2988 [M + Na]^+^ (calcd for C_35_H_47_NNaO_12_^+^, 696.2990).

Compound **5**: brown powder; [α]^20^_*D*_ = +206.3 (*c* 0.10, MeOH); UV (MeOH) *λ*_max_ (logε) 225 (4.44), 263 (4.24), 326 (3.93) nm; IR (KBr) *ν*_max_ 3368, 2970, 2934, 1631, 1607, 1495, 1388, 1197, 1053, 974, 887 cm^−1^; ^1^H NMR data, Table 3; ^13^C NMR data, Table 2; HRESIMS: *m*/*z* 698.3170 [M + H]^+^ (calcd for C_37_H_48_NO_12_^+^, 698.3171), and 720.2986 [M + Na]^+^ (calcd for C_37_H_47_NNaO_12_^+^, 720.2990).

Compound **6**: brown powder; [α]^20^_*D*_ = +174.2 (*c* 0.10, MeOH); UV (MeOH) *λ*_max_ (logε) 222 (4.42), 312 (3.91) nm; IR (KBr) *ν*_max_ 3347, 2972, 2932, 1629, 1495, 1387, 1323, 1196, 968, 884 cm^−1^; ^1^H NMR data, Table 3; ^13^C NMR data, Table 2; HRESIMS: *m*/*z* 654.2914 [M + H]^+^ (calcd for C_35_H_44_NO_11_^+^, 654.2909), and 676.2731 [M + Na]^+^ (calcd for C_35_H_48_NNaO_11_^+^, 676.2728).

Compound **7**: brown powder; [α]^20^_*D*_ = +246.3 (*c* 0.11, MeOH); UV (MeOH) *λ*_max_ (logε) 222 (4.38), 319 (3.89) nm; IR (KBr) *ν*_max_ 3362, 2967, 2935, 1631, 1497, 1389, 1318, 1193, 1054, 976, 801 cm^−1^; ^1^H NMR data, Table 3; ^13^C NMR data, Table 2; HRESIMS: *m*/*z* 672.3019 [M + H]^+^ (calcd for C_35_H_46_NO_12_^+^, 672.3015), and 694.2839 [M + Na]^+^ (calcd for C_35_H_45_NNaO_12_^+^, 694.2834).

### 2.5. Bioactivity

#### 2.5.1. Antimicrobial Assay

Compounds **1**–**13** were assayed for their antimicrobial activity against *Staphylococcus aureus* ATCC 25923, *Mycobacterium smegmatis* mc^2^ 155, *Pseudomonas aeruginosa* PA01 and *Proteusbacillus vulgaris* CPCC 160013 with the paper disk diffusion assay as previously described [21]. The tested compounds (20 µg/µL, 2 µL each) were absorbed onto individual paper disks (Ø 6 mm) and placed on the surface of the agar. The assay plates were incubated for 24 h at 37 °C and examined for the presence of inhibitory zones.

The MIC values of active compounds against the growth of *Staphylococcus aureus* ATCC 25923 were measured through the microbroth dilution method [22]. Microorganisms were cultured in LB media in 96-well plates at a concentration of 1 × 10^6^ CFU/mL. The MIC values were obtained after incubating for 12 h at 37 °C with the tested compounds (concentration ranging from 320 to 0.039 µg/mL).

#### 2.5.2. Cytotoxicity Assay

The in vitro antiproliferative activity against HeLa and Caco-2 cells was measured as previously reported [23,24]. Briefly, cells were seeded in 96-well plates at 7 × 10^3^ cells/well and treated for 24 h with different concentrations of compounds **1**–**13**. Then, 10 µL Cell Counting Kit-8 (CCK-8) was added to each well and incubated for another 4 h. The absorbance was read at 480 nm by Spark 30086376 (TECAN, Männedorf, Switzerland).

## 3. Results

**Figure 2:**
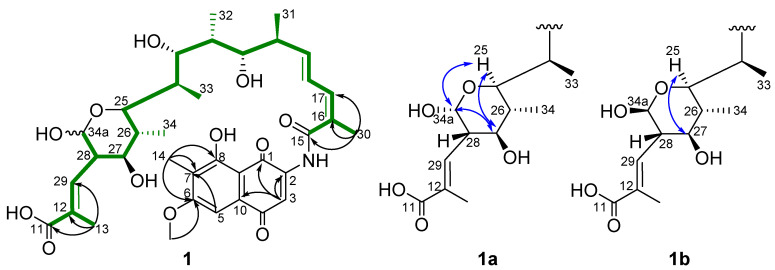
Selected HMBC (→), COSY (▬) and NOESY (↔) correlations of **1**.

Compound **1** was determined to have the molecular formula C_36_H_47_NO_12_ on the basis of the *quasi* molecular ion peaks at HRESIMS *m*/*z* 686.3172 [M + H]^+^ (calcd for C_36_H_48_NO_12_^+^_,_ 686.3171) and 708.2992 [M + Na]^+^ (calcd for C_36_H_48_NNaO_12_^+^, 708.2990). The presence of a naphthaquinone chromophore was indicated by the HMBC correlations from H-3 (*δ*_H_ 7.64) to C-1 (*δ*_C_ 184.7), C-2 (*δ*_C_ 143.0) and C-10 (*δ*_C_ 132.6), and from H-14 (*δ*_H_ 2.09) to C-6 (*δ*_C_ 165.9), C-7 (*δ*_C_ 120.3) and C-8 (*δ*_C_ 161.7). The MeO-6 (*δ*_H_ 4.00) was supported by the HMBC correlations from MeO-6 to C-6 and NOE correlations from MeO-6 to H-5 (*δ*_H_ 7.18) (Table 1 and Table 2, Tables S2 and S3, Figure 2). The twenty-four-carbon fragment from C-15 (*δ*_C_ 170.0) to C-11 (*δ*_C_ 172.3) was established on the basis of ^1^H-^1^H COSY correlations, along with the HMBC correlations from the H-30 (Me), H-31 (Me), H-32 (Me), H-33 (Me), H-34 (Me) and H-34a to the corresponding carbons (green, Figure 2). The hydroxylation of C-34a and oxidization to an aldehyde group followed by hemiacetal formation with the hydroxyl group at C-25 were determined based on the ^1^H NMR of H-34a (*δ*_H_ 4.54/5.08) (Table 1, Figure 2). The *ansa* chain was determined to undergo retro-Claisen cleavage between C-5 and C-11 on the basis of the chemical shift of C-11 downfield, the presence of the extra aromatic proton H-5 compared to that of normal rifamycins and HMBC from H-5 to C-7 (Tables S2 and S3). Hence, the planar structure of **1** was established. The stereochemistry of the hemiacetal existed as a pair of epimers (**1a** and **1b**) at C-34a, and **1a** was determined to be α-form on the basis of the coupling constants *J*_34a,__28_ = 8.4 Hz and the NOE correlations from H-34a (*δ*_H_ 4.54) to H-25 (*δ*_H_ 3.55) and H-27 (*δ*_H_ 3.18), and between H-25 and H-27. Accordingly, **1b** was determined to be β-form on the basis of the coupling constants *J*_34a,__28_ = 3.2 Hz (Figure 2). The stereochemistry of other carbons was assumed to be the same as that of rifamycin W-hemiacetal [25] based on biosynthetic logic [12]. Thus, compound **1a** was named 34a-α-6-*O*-methyl-rifamycin W-M1-hemiacetal and **1b** was named 34a-β-6-*O*-methyl-rifamycin W-M1-hemiacetal.

Compound **2** was confirmed to have the molecular formula C_35_H_45_NO_13_ on the basis of the HRESIMS *quasi* molecular ion peaks at *m*/*z* 688.2956 [M + H]^+^ and 710.2781 [M + Na]^+^. The NMR spectroscopic data of **2** were similar to that of **1**, except that C-34 was a hydroxymethyl (*δ*_H_ 4.33, 4.34, *δ*_C_ 66.0) instead of a methyl group, and the 6-hydroxyl group was free (Table 1 and Table 2). The relative configuration of **2** was proposed to be identical to that of **1**, and the hemiacetal existed as a pair of epimers (**2a** and **2b**) (Tables S4 and S5) as well. Thus, compound **2** was determined to be 34a-α-30-hydroxyrifamycin W-M1-hemiacetal (**2a**) and 34a-β-30-hydroxyrifamycin W-M1-hemiacetal (**2b**), respectively.

Similarly, the NMR (Tables S6 and S7) and HRESIMS (*m*/*z* 672.3011 [M + H]^+^ and 694.2835 [M + Na]^+^) comparison determined compound **3** to be 34a-α-rifamycin W-M1-hemiacetal (**3a**) and 34a-β-rifamycin W-M1-hemiacetal (**3b**), respectively.

The molecular formula of **4** was elucidated as C_3__5_H_4__7_NO_1__2_ on the basis of the HRESIMS *quasi* molecular ion peaks at *m*/*z* 674.3172 [M + H]^+^ and 696.2988 [M + Na]^+^. Similar to that of compounds **1**, **2** and **3**, the *ansa* chain of **4** also underwent retro-Claisen cleavage between C-5 and C-11 due to the presence of the extra aromatic proton H-5 (*δ*_H_ 7.07), the chemical shift of C-11 (*δ*_C_ 173.0) and the HMBC from H-5 to C-7 (Table 2 and Table 3, Table S8). Thus, compound **4** was determined to be rifamycin W-M1 [26].

The molecular formula of **5** was confirmed to be C_37_H_47_NO_12_ by the HRESIMS *quasi* molecular ion peaks at *m*/*z* 698.3170 [M + H]^+^ and 720.2986 [M + Na]^+^. A close NMR comparison with that of rifamycin W (**12**) (Tables S9 and S12) [27] revealed that **5** was 34a-*O*-acetyl-rifamycin W, which was confirmed by the HMBC correlations between H-34a (*δ*_H_ 4.01, 4.00) and the acetyl carbon (*δ*_C_ 172.9).

The molecular formula of **6** was elucidated as C_35_H_43_NO_11_ on the basis of the HRESIMS *quasi* molecular ion peaks at *m*/*z* 654.2914 [M + H]^+^ and 676.2731 [M + Na]^+^. NMR comparison with rifamycin W (**12**) (Tables S10 and S12) revealed that **6** was 23-ketorifamycin W on the basis of the downfield chemical shifts of C-22 (*δ*_C_ 49.7), C-23 (*δ*_C_ 211.3) and C-24 (*δ*_C_ 49.9).

Compound **7** was determined to have the molecular formula of C_35_H_45_NO_12_ on the basis of HRESIMS *quasi* molecular ion peaks at *m*/*z* 672.3019 [M + H]^+^ and 694.2839 [M + Na]^+^, revealing one more oxygen atom than that of rifamycin W. NMR comparison (Tables S11 and S12) determined **7** to be 20-hydroxyrifamycin W, which was supported by the chemical shift of C-30 (*δ*_C_ 77.0).

Based on the 1D and 2D NMR data, HRESIMS data and spectroscopic comparisons with those reported in the literature, compounds **8**–**13** were determined to be rifamycin Z (**8**) [28], 30-hydroxyrifamycin W-hemiacetal (**9**) [29], rifamycin W-hemiacetal (**10**) [25,30], 30-hydroxyrifamycin W (**11**) [30], rifamycin W (**12**) [25,27] and protorifamycin I (**13**) [31] (Figure S7).

Compounds **1**−**13** were assayed for their antimicrobial activity against *Staphylococcus aureus* ATCC 25923, *Mycobacterium smegmatis* mc^2^ 155, *Pseudomonas aeruginosa* PA01 and *Proteusbacillus vulgaris* CPCC 160013. The results showed that new compounds **1**–**3** and known compounds **11** and **13** exhibited inhibitory activity against *S. aureus* ATCC 25923, while other compounds showed no antimicrobial activity (Figure S56). Thus, new compounds **1**–**3** were further tested for their antibacterial activity against *S. aureus* ATCC 25923 using the microbroth dilution method [22], and their MIC values were determined to be 5, 40 and 0.5 µg/mL, respectively (Table S14).

In view of no evident bactericidal activity, compounds **1**–**13** were evaluated for their antiproliferative activity against HeLa and Caco-2 cells using Cell Counting Kit-8 (CCK-8) (Bimake, Houston, TX, USA) and etoposide (VP-16) as a positive control. Compounds **1** and **3** showed modest activity in inhibiting the proliferation of HeLa and Caco-2 cells with IC_50_ values of about 50 µM (Table S15, Figures S57 and S58).

## 4. Discussion

Post-PKS modifications play an important role in increasing the structural diversity and improving the biological activity of rifamycins. As the proposed earliest macrocyclic intermediate in rifamycin post-PKS biosynthesis, proansamycin X tended to undergo dehydration to form putative protorifamycins (without C-8 hydroxyl group) or undergo dehydrogenation to form rifamycin W [24,32,33]. Rifamycin W undergoes a rearrangement of the polyketide backbone to produce rifamycin B via the oxidative cleavage of the C-12/C-29 double bond. The mechanism of this oxidative cleavage has not been characterized yet. For the *rif*-*orf5* gene, when cloned and heterologously expressed in *E. coli*, the recombinant protein showed spectra typical of P450 cytochromes [34]. Thus, the *rif*-*orf5* gene was confirmed to code for a cytochrome P450 enzyme, which is the key step for oxygen incorporation in rifamycin B biosynthesis and may be involved in the cleavage of the olefinic moiety of rifamycin W [16,17].

**Figure 3:**
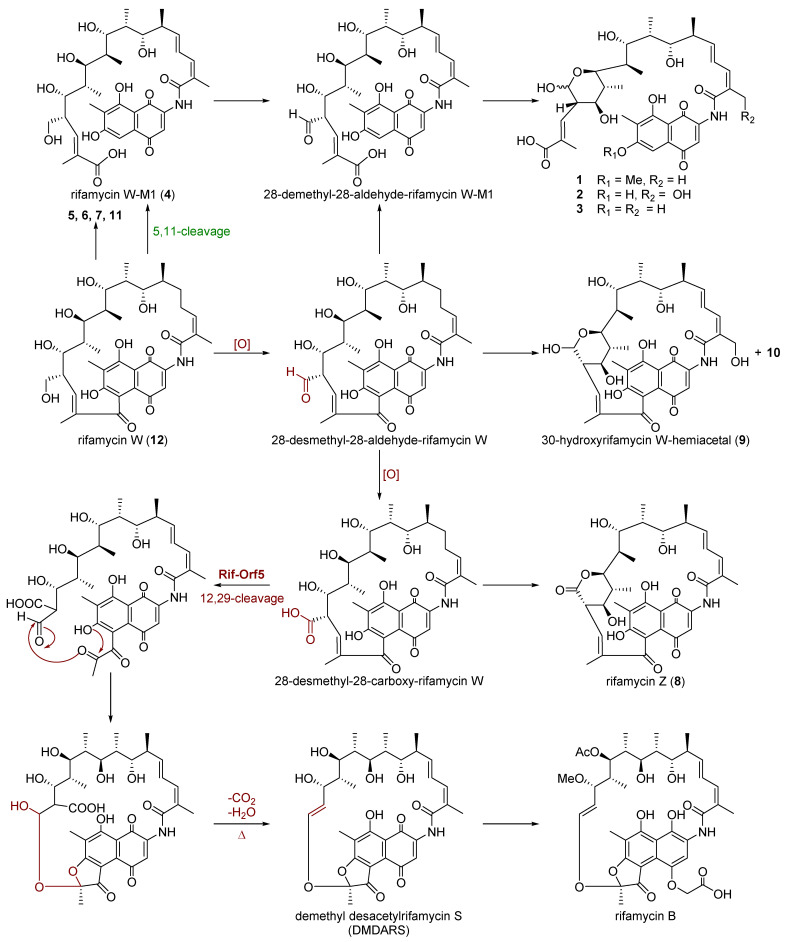
Proposed biosynthetic pathway of compounds from mutant Δ*rif*-*orf5*.

In this study, systematic isolation of the fermentation products of the mutant strain Δ*rif*-*orf5* afforded thirteen rifamycin W derivatives besides the main product rifamycin W (**12**), indicating that the *rif*-*orf5* gene was probably involved in the oxidative cleavage of the C-12/C-29 double bond. Compounds **1**–**4** all undergo C-5/C-11 retro-Claisen cleavage, as observed in the biosynthesis of proansamycin B-M1 and protorifamycin I–M1 [24,35], hygrocins I and J [36], divergolides R and S [37] and microansamycins G–I [38]. This C-5/C-11 cleavage probably occurred due to an over-accumulation of rifamycin W, which serves as a detoxification mechanism. Compounds **1**–**3**, **9** and **10** featured a hemiacetal, in which **9** and **10** existed in β-form according to the chemical shift of C-34a and the coupling constants between C-34a and C-28, while **1**–**3** existed as epimer pairs (Table S13), which may be due to the feasibility of polyketide chain cleavage in C-5/C-11. Additionally, the hemiacetal containing compounds **1**–**3**, **9** and **10**, as well as the lactone-containing rifamycin Z (**8**), indicated that the oxidation of C-34a alcohol to the carboxyl group may occur before the C-12/C-29 olefinic bond cleavage. In addition, compared to 8-deoxy rifamycins [24], compounds **5**, **6**, **7** and **11** also oxygenated at C-34a, C-23, C-20 and C-30, which suggested that the rifamycin *ansa* chain is prone to oxidization in these specific sites during fermentation (Figure 3).

## 5. Conclusions

In this study, the cytochrome P450 monooxygenase gene *rif*-*orf5* was confirmed to be involved in the oxidative cleavage of the *ansa* chain of rifamycin W through in vivo gene inactivation and isolation of the main product rifamycin W. Systematic isolation of the fermentation products of the mutant strain Δ*rif*-*orf5* afforded seven new rifamycin W congeners, from which **1**–**3** featured two epimeric forms of hemiacetal at C-34a, and C-5/C-11 retro-Claisen cleavage. Compounds **1**–**3** exhibited antibacterial activity against *Staphylococcus aureus*, and **1** and **3** showed modest antiproliferative activity against HeLa and Caco-2 cells.

## Figures and Tables

**Table 1:** ^1^H NMR spectroscopic data (400 MHz, CD_3_OD) of compounds **1**–**3** (*δ*_H_, *J* in Hz) *.

Position	1		2		3	
	1a	1b	2a	2b	3a	3b
3	7.64 (s)	7.64 (s)	7.64 (s)	7.64 (s)	7.61 (s)	7.61 (s)
5	7.18 (s)	7.18 (s)	6.99 (s)	6.99 (s)	7.02 (s)	7.02 (s)
MeO-6	4.00 (s)	4.00 (s)				
13	1.89 (s)	1.87 (s)	1.90 (s)	1.88 (s)	1.90 (s)	1.88 (s)
14	2.09 (s)	2.09 (s)	2.07 (s)	2.07 (s)	2.09 (s)	2.09 (s)
17	6.50 (d, 10.8)	6.50 (d, 10.8)	6.64 (d, 11.3)	6.64 (d, 11.3)	6.50 (d, 10.8)	6.50 (d, 10.8)
18	6.87 (dd, 11.2, 14.9)	6.87 (dd, 11.2, 14.9)	7.21 (dd, 11.1, 15.0)	7.21 (dd, 11.1, 15.0)	6.86 (dd, 12.4, 14.9)	6.86 (dd, 12.4, 14.9)
19	6.08 (m)	6.08 (m)	6.23 (m)	6.23 (m)	6.07 (m)	6.07 (m)
20	2.44 (m)	2.44 (m)	2.52 (m)	2.52 (m)	2.47 (m)	2.47 (m)
21	3.82 (m)	3.82 (m)	3.84 (d, 8.7)	3.84 (d, 8.7)	3.83 (m)	3.83 (m)
22	2.04 (m)	2.04 (m)	1.95 (m)	1.95 (m)	1.96 (m)	1.96 (m)
23	3.62 (m)	3.62 (m)	3.64 (m)	3.64 (m)	3.62 (m)	3.62 (m)
24	1.98 (m)	1.98 (m)	2.03 (m)	2.03 (m)	2.02 (m)	2.02 (m)
25	3.55 (m)	4.21 (d, 10.4)	3.56 (m)	4.22 (d, 10.3)	3.56 (m)	4.21 (m)
26	1.59 (m)	1.63 (m)	1.60 (m)	1.62 (m)	1.60 (m)	1.61 (m)
27	3.18 (t, 9.8)	3.55 (m)	3.20 (t, 9.8)	3.56 (m)	3.17 (m)	3.56 (m)
28	2.42 (m)	2.64 (td, 3.5, 10.0)	2.47 (m)	2.65 (td, 3.5, 10.2)	2.42 (m)	2.61 (m)
29	6.62 (d, 10.7)	6.79 (d, 10.1)	6.61 (d, 11.3)	6.80 (d, 10.0)	6.62 (d, 10.7)	6.78 (d, 10.1)
30	2.08 (s)	2.08 (s)	4.34/4.33 (s)	4.34/4.33 (s)	2.08 (s)	2.08 (s)
31	1.02 (s)	1.02 (s)	1.03 (d, 7.0)	1.03 (d, 7.0)	1.02 (s)	1.02 (s)
32	1.00 (s)	1.00 (s)	1.01 (d, 7.1)	1.01 (d, 7.1)	1.00 (s)	1.00 (s)
33	0.98 (s)	0.98 (s)	0.98 (d, 6.7)	0.98 (d, 6.7)	0.98 (s)	0.98 (s)
34	0.96 (s)	0.96 (s)	0.97 (d, 6.9)	0.97 (d, 6.9)	0.96 (s)	0.96 (s)
34a	4.54 (d, 8.4)	5.08 (d, 3.2)	4.56 (d, 6.9)	5.09 (d, 3.3)	4.54 (d, 6.9)	5.08 (d, 3.3)

* s: singlet, d: doublet, dd: double doublet, t: triplet, m: multiplet.

**Table 2:** ^13^C NMR spectroscopic data (100 MHz, CD_3_OD) of compounds **1**–**7** (*δ*_C_) *.

Position	1		2		3		4	5	6	7
	1a	1b	2a	2b	3a	3b				
1	184.7s	184.7s	184.1s	184.1s	184.1s	184.1s	183.7s	184.1s	184.0s	184.7s
2	143.0s	143.0s	143.2s	143.2s	142.4s	142.4s	142.7s	142.4s	142.4s	143.0s
3	117.3d	117.3d	117.2d	117.2d	117.2d	117.2d	117.2d	118.6d	119.1d	119.2d
4	187.1s	187.1s	186.8s	186.8s	186.5s	186.5s	186.8s	186.6s	186.9s	187.5s
5	103.7d	103.7d	108.6d	108.6d	108.8d	108.8d	108.9d	108.3s	108.1s	108.7s
6	165.9s	165.9s	163.9s	163.9s	164.0s	164.0s	164.0s	161.8s	163.6s	163.1s
MeO-6	57.0q	57.0q								
7	120.3s	120.3s	118.2s	118.2s	118.3s	118.3s	118.4s	119.2s	119.3s	119.2s
8	161.7s	161.7s	165.0s	165.0s	165.2s	165.2s	165.6s	164.0s	164.0s	164.7s
9	131.7s	131.7s	130.6s	130.6s	131.1s	131.1s	126.1s	125.5s	124.7s	126.0s
10	132.6s	132.6s	132.3s	132.3s	132.4s	132.4s	132.4s	129.9s	130.8s	130.9s
11	172.3s	172.3s	171.8s	171.8s	172.3s	172.3s	173.0s	200.1s	200.3s	201.1s
12	133.3s	132.4s	133.1s	131.5s	132.4s	131.1s	131.8s	141.6s	142.0s	142.0s
13	13.9q	13.3q	13.9q	13.3q	13.9q	13.3q	13.9q	12.7q	13.0q	13.4q
14	8.3q	8.3q	8.2q	8.2q	8.2q	8.2q	8.1q	8.7q	8.7q	9.3q
15	170.0s	170.0s	169.0s	169.0s	170.0s	170.0s	170.1s	172.2s	172.8s	172.2s
16	129.3s	129.3s	142.6s	142.6s	129.4s	129.4s	129.5s	132.2s	133.1s	133.4s
17	139.0d	139.0d	143.7d	143.7d	139.1d	139.1d	138.9d	135.2d	133.4d	136.2d
18	127.6d	127.6d	128.1d	128.1d	127.8d	127.8d	127.6d	126.3d	127.3d	136.2d
19	146.3d	146.3d	150.1d	150.1d	146.4d	146.4d	146.5d	141.6d	140.7d	126.1d
20	42.5d	42.5d	42.7d	42.7d	42.5d	42.5d	42.5d	39.2d	43.4d	77.0s
21	75.8d	75.8d	75.8d	75.8d	75.8d	75.8d	75.8d	74.9d	78.3d	76.7d
22	36.6d	36.6d	36.7d	36.7d	36.5d	36.5d	36.9d	34.4d	49.7d	35.3d
23	78.5d	78.5d	78.5d	78.5d	78.5d	78.5d	79.3 d	79.0d	211.3s	80.8d
24	37.0d	37.0d	37.1d	37.1d	37.0d	37.0d	36.7d	38.0d	49.9d	39.0d
25	73.4d	72.8d	73.3d	72.8d	73.4d	72.7d	73.3d	71.3d	71.2d	72.3d
26	40.3d	41.3d	40.3d	41.3d	40.4d	41.3d	40.9d	43.9d	42.7d	44.6d
27	77.5d	73.4d	77.1d	73.3d	77.1d	73.4d	72.1d	68.7d	68.3d	70.0d
28	53.9d	50.7d	53.9d	50.7d	53.9d	50.7d	47.0d	46.1d	49.3d	49.1d
29	141.0d	142.4d	141.2d	142.7d	141.1d	142.4d	142.7d	139.0d	140.2d	141.5d
30	20.7q	20.7q	66.0t	66.0t	20.7q	20.7q	20.7q	20.3q	20.4q	21.0q
31	17.3q	17.3q	17.2q	17.2q	17.2q	17.2q	17.5q	18.2q	20.2q	26.7q
32	11.2q	11.2q	11.2q	11.2q	11.2q	11.2q	10.5q	11.3q	14.8q	14.6q
33	10.5q	10.5q	10.7q	10.7q	10.9q	10.9q	10.7q	8.9q	8.4q	9.8q
34	12.9q	12.9q	12.8q	12.8q	12.8q	12.8q	10.8q	11.8q	11.9q	12.4q
34a	98.2d	94.5d	98.0d	94.6d	98.0d	94.5d	64.6t	65.8t	64.4t	65.1t
AcO-34a								21.0q 172.9s		

* s: quaternary carbon, d: tertiary carbon, t: secondary carbon, q: primary carbon.

**Table 3:** ^1^H NMR spectroscopic data (400 MHz, CD_3_OD) of compounds **4**–**7** (*δ*_H_, *J* in Hz) *.

Position	4	5	6	7
3	7.65 (s)	7.57 (s)	7.56 (s)	7.57 (s)
5	7.07 (s)			
13	1.89 (s)	2.08 (s)	2.04 (s)	2.06 (d, 1.0)
14	2.12 (s)	2.18 (s)	2.17 (s)	2.17 (s)
17	6.53 (t, 14.3)	6.25 (d, 10.8)	6.24 (d, 10.8)	6.26 (dd, 0.8, 10.9)
18	6.84 (dd, 10.9, 14.3)	6.51 (dd, 11.0, 15.8)	6.09 (dd, 11.0, 15.1)	5.96 (d, 16.0)
19	6.08 (dd, 8.2, 15.0)	6.09 (dd, 6.6, 15.9)	5.85 (dd, 9.6, 15.2)	6.47 (dd, 10.9, 15.9)
20	2.45 (m)	2.36 (m)	1.89 (m)	
21	3.82 (d, 8.8)	4.03 (m)	3.61 (dd, 1.5, 9.2)	3.95 (d, 1.2)
22	1.90 (m)	1.87 (m)	2.86 (dd, 6.8, 9.2)	2.01 (m)
23	3.60 (m)	3.48 (d, 10.2)		3.42 (q, 2.7, 9.4)
24	1.84 (m)	1.80 (m)	2.52 (m)	1.72 (m)
25	4.05 (d, 9.7)	3.98 (m)	3.87 (d, 10.2)	3.94 (dd, 1.9, 10.6)
26	1.80 (m)	1.43 (m)	1.35 (m)	1.40 (m)
27	4.13 (d, 5.5)	4.31 (s)	4.43 (s)	4.37 (br s)
28	2.82 (m)	2.89 (m)	2.58 (m)	2.65 (q, 7.1, 16.0)
29	6.91 (d, 10.4)	6.30 (d, 9.3)	6.28 (d, 9.1)	6.35 (dd, 1.0, 9.5)
30	2.09 (s)	2.09 (s)	2.05 (s)	2.10 (s)
31	1.00 (d, 6.8)	0.91 (d, 6.9)	1.06 (d, 3.1)	1.21 (s)
32	0.90 (d, 6.8)	1.05 (d, 7.0)	1.05 (d, 3.2)	1.17 (d, 7.0)
33	0.96 (d, 6.8)	0.72 (d, 6.8)	1.12 (d, 7.4)	0.74 (d, 6.8)
34	0.83 (d, 6.8)	0.40 (d, 7.0)	0.41 (d, 7.0)	0.41 (d, 7.0)
34a	3.62 (m) 3.54 (m)	4.01 (m) 4.00 (m)	3.52 (dd, 8.6, 10.9) 3.38 (dd, 6.1, 11.0)	3.40 (m) 3.58 (dd, 8.0, 10.9)
AcO-34a		2.03 (s)		

* s: singlet, d: doublet, dd: double doublet, t: triplet, m: multiplet.

## Data Availability

Not applicable.

## Supplementary Materials

The following are available online at https://www.mdpi.com/article/10.3390/biom11070920/s1, Figure S1: Construction flow chart for *rif*-*orf5* gene knock-out mutant Δ*rif*-*orf5*; Figure S2: Construction and enzymatic digestion verification of pOJ260-orf5, which was verified by sequencing; Figure S3: PCR verification of *rif*-*orf5* gene knock-out mutant Δ*rif*-*orf5*; Figure S4: Construction and enzymatic digestion verification of pSET152-orf5, which was verified by sequencing; Figure S5: PCR verification of *rif*-*orf5* gene complementation mutant Δ*rif-orf5*::*orf5*; Figure S6: HPLC detection of *rif*-*orf5* gene knock-out and gene complementation mutants; Figure S7: Structures of known compounds; Figure S8–S14: NMR and HRESIMS spectra of **1**; Figure S15–S21: NMR and HRESIMS spectra of **2**; Figure S22–S28: NMR and HRESIMS spectra of **3**; Figure S29–S34: NMR and HRESIMS spectra of **4**; Figure S35–S41: NMR and HRESIMS spectra of **5**; Figure S42–S48: NMR and HRESIMS spectra of **6**; Figure S49–S55: NMR and HRESIMS spectra of **7**; Figure S56: Antimicrobial activity of compounds **1**–**13**; Figure S57: Antiproliferative activity of compounds **1**–**13** (50 and 10 μM, respectively) against HeLa cells; Figure S58: Antiproliferative activity of compounds **1**–**13** (50 and 10 μM, respectively) against Caco-2 cells; Table S1: Primers used in this study; Table S2–S11: NMR data of compounds **1**–**7**; Table S12: NMR spectroscopic data for rifamycin W (**12**); Table S13: Selected ^1^H NMR spectroscopic data for hemiacetal of compounds **1**–**3** and **9**, **10**; Table S14: Diameter of the inhibition zones and MIC of active compounds **1**–**3** against *Staphylococcus aureus* ATCC 25923; Table S15: Antiproliferative activity against HeLa and Caco-2 cells of compounds **1**–**13**.

## References

[1] Rinehart K.L., Shield L.S., Herz W., Grisebach H., Kirby G.W. (1976). Chemistry of the ansamycin antibiotics. Fortschritte der Chemie Organischer Naturstoffe/Progress in the Chemistry of Organic Natural Products.

[2] Wehrli W. (1978). ChemInform Abstract: Ansamycins: Chemistry, biosynthesis and biological activity. Chem. Inf..

[3] Sensi P. (1957). Applications of paper chromatography & countercurrent distribution to steroids & antibiotics. Boll. Chim. Farm..

[4] Sensi P., Greco A.M., Gallo G.G., Rolland G. (1957). Isolation and structure determination of a new amicetin-like antibiotic: Amicetin B. Antibiot. Chemother..

[5] Sensi P., Margalith P., Timbal M.T. (1959). Rifomycin, a new antibiotic. Preliminary report. Farmaco Sci..

[6] Wehrli W., Staehelin M. (1969). The rifamycins—Relation of chemical structure and action on RNA polymerase. Biochim. et Biophys. Acta (BBA) Nucleic Acids Protein Synth..

[7] Ramos-e-Silva M., Rebello P.F. (2001). Leprosy. Recognition and treatment. Am. J. Clin. Dermatol..

[8] Murphy C.K., Karginova E., Sahm D., Rothstein D.M. (2007). In Vitro Activity of Novel Rifamycins against Gram-positive Clinical Isolates. J. Antibiot..

[9] Czerwonka D., Domagalska J., Pyta K., Kubicka M.M., Pecyna P., Gajecka M., Przybylski P. (2016). Structure–activity relationship studies of new rifamycins containing (L) -amino acid esters as inhibitors of bacterial RNA polymerases. Eur. J. Med. Chem..

[10] Girling D.J. (1977). Adverse reactions to rifampicin in antituberculosis regimens. J. Antimicrob. Chemother..

[11] Goldstein B.P. (2014). Resistance to rifampicin: A review. J. Antibiot..

[12] August P.R., Tang L., Yoon Y.J., Ning S., Müller R., Yu T.-W., Taylor M., Hoffmann D., Kim C.-G., Zhang X. (1998). Biosynthesis of the ansamycin antibiotic rifamycin: Deductions from the molecular analysis of the rif biosynthetic gene cluster of *Amycolatopsis mediterranei* S699. Chem. Biol..

[13] Li T., Yoon Y.J., Choi C.-Y., Hutchinson C.R. (1998). Characterization of the enzymatic domains of in the modular polyketide synthase involved in rifamycin B biosynthesis by *Amycolatopsis mediterranei*. Gene.

[14] Schupp T., Toupet C., Engel N., Goff S. (1998). Cloning and sequence analysis of the putative rifamycin polyketide synthase gene cluster from *Amycolatopsis mediterranei*. FEMS Microbiol. Lett..

[15] Floss H.G., Yu T.-W. (1999). Lessons from the rifamycin biosynthetic gene cluster. Curr. Opin. Chem. Biol..

[16] Floss H.G., Yu T.-W. (2005). Rifamycin-Mode of Action, Resistance, and Biosynthesis. Chem. Rev..

[17] Xu J., Wan E., Kim C.-J., Floss H.G., Mahmud T. (2005). Identification of tailoring genes involved in the modification of the polyketide backbone of rifamycin B by *Amycolatopsis mediterranei* S699. Microbiology.

[18] Bierman M., Logan R., O’Brien K., Seno E., Rao R.N., Schoner B. (1992). Plasmid cloning vectors for the conjugal transfer of DNA from *Escherichia coli* to *Streptomyces* spp.. Gene.

[19] Hu Z., Hunziker D., Hutchinson C.R., Khosla C. (1999). A host–vector system for analysis and manipulation of rifamycin polyketide biosynthesis in *Amycolatopsis mediterranei*. Microbiology.

[20] Gibson D.G., Young L., Chuang R.Y., Venter J.C., Hutchison C.A., Smith H.O. (2009). Enzymatic assembly of DNA molecules up to several hundred kilobases. Nat. Methods.

[21] Raahave D. (1974). Paper Disk-Agar Diffusion Assay of Penicillin in the Presence of Streptomycin. Antimicrob. Agents Chemother..

[22] Arendrup M.C., Prakash A., Meletiadis J., Sharma C., Chowdhary A. (2017). Comparison of EUCAST and CLSI reference micro-dilution MICs of eight antifungal compounds for Candida auris and associated tentative epidemiological cutoff values. Anti-microb. Agents Chemother..

[23] Jiang Z., Zhou Q., Ge C., Yang J., Li H., Chen T., Xie H., Cui Y., Shao M., Li J. (2019). Rpn10 promotes tumor progression by regulating hypoxia-inducible factor 1 alpha through the PTEN/Akt signaling pathway in hepatocellular carcinoma. Cancer Lett..

[24] Ye F., Shi Y., Zhao S., Li Z., Wang H., Lu C., Shen Y. (2020). 8-Deoxy-rifamycin derivatives from *Amycolatopsis mediterranei* S699 Δ*rifT* strain. Biomolecules.

[25] Stratmann A., Schupp T., Toupet C., Schilling W., Oberer L., Traber R. (2002). New Insights into Rifamycin B Biosynthesis: Isolation of Proansamycin B and 34a-Deoxy-rifamycin W as Early Macrocyclic Intermediates Indicating Two Separated Biosynthetic Pathways. J. Antibiot..

[26] Ghisalba O., Traxler P., Fuhrer H., Richter W.J. (1980). Early intermediates in the biosynthesis of ansamycins. III. Isolation and identification of further 8-deoxyansamycins of the rifamycin-type. J. Antibiot..

[27] Richard J.W., Edoardo M., Giancarlo L. (1974). Ansamycin biogenesis: Studies on a novel rifamycin isolated from a mutant strain of *Nocardia mediterranei*. Proc. Natl. Acad. Sci. USA.

[28] Cricchio R., Antonini P., Ferrari P., Ripamonti A., Tuan G., Martinelli E. (1981). Rifamycin Z, a novel ansamycin from a mutant of *Nocardia mediterranea*. J. Antibiot..

[29] Shi Y., Zhang J., Tian X., Wu X., Li T., Lu C., Shen Y. (2019). Isolation of 11,12-seco-Rifamycin W Derivatives Reveals a Cleavage Pattern of the Rifamycin Ansa Chain. Org. Lett..

[30] Traxler P., Schupp T., Fuhrer H., Richter W.J. (1981). 3-Hydroxyrifamycin S and further novel ansamycins from a recombinant strain R-21 of *Nocardia mediterranei*. J. Antibiot..

[31] Ghisalba O., Traxler P., Nuesch J. (1978). Early intermediates in the biosynthesis of ansamycins. I. Isolation and identification of protorifamycin I. J. Antibiot..

[32] Stratmann A., Toupet C., Schilling W., Traber R., Oberer L., Schupp T. (1999). Intermediates of rifamycin polyketide synthase produced by an *Amycolatopsis mediterranei* mutant with inactivated *rifF* gene. Microbiology.

[33] Yu T.-W., Shen Y., Doi-Katayama Y., Tang L., Park C., Moore B., Hutchinson C.R., Floss H.G. (1999). Direct evidence that the rifamycin polyketide synthase assembles polyketide chains processively. Proc. Natl. Acad. Sci. USA.

[34] Mejía A., Luna D., Fernández F.J., Barrios-González J., Gutierrez L.H., Reyes A.G., Absalón A.E., Kelly S. (2018). Improving rifamycin production in *Amycolatopsis mediterranei* by expressing a Vitreoscilla hemoglobin (vhb) gene fused to a cytochrome P450 monooxygenase domain. 3 Biotech.

[35] Ghisalba O., Traxler P., Fuhrer H., Richter W.J. (1979). Early intermediates in the biosynthesis of ansamycins. II. Isolation and identification of proansamycin B-M1 and protorifamycin I-M1. J. Antibiot..

[36] Li S., Lu C., Ou J., Deng J., Shen Y. (2015). Overexpression of hgc1 increases the production and diversity of hygrocins in *Streptomyces* sp. LZ35. RSC Adv..

[37] Zhao G., Li S., Guo Z., Sun M., Lu C. (2015). Overexpression of div8 increases the production and diversity of divergolides in *Streptomyces* sp. W112. RSC Adv..

[38] Wang J., Li W., Wang H., Lu C. (2018). Pentaketide Ansamycin Microansamycins A–I from Micromonospora sp. Reveal Diverse Post-PKS Modifications. Org. Lett..

