# Supplementary material for: Rifamycin W Analogues from Amycolatopsis mediterranei S699 Δrif-orf5 Strain
Source: Biomolecules. 2021 Jun 22;11(7):920. doi: 10.3390/biom11070920 (PMC8301457; doi:10.3390/biom11070920)
Supplement: Supplementary file 1 [file biomolecules-11-00920-s001.zip › biomolecules-1240431-supplementary.pdf]

Supporting Information

Rifamycin W Analogues from *Amycolatopsis mediterranei* S699 Δ*rif-orf5* Strain

Yanrong Shi [1,2], Feng Ye [1], Yuliang Song [1], Xiaochun Zhang [1], Chunhua Lu [1] and Yuemao Shen[1,*]

[1] Department of Natural Product Chemistry, Key Laboratory of Chemical Biology (Ministry of Education), School of Pharmaceutical Sciences, Cheeloo College of Medicine, Shandong University, Jinan, Shandong 250012, China; yrshi910212@sdu.edu.cn (Y.S.); yefeng1997@mail.sdu.edu.cn (F.Y.); 201936124@mail.sdu.edu.cn (Y.S.); 201816082@mail.sdu.edu.cn (X.Z.); ahua0966@sdu.edu.cn (C.L.)

[2] Key Laboratory Experimental Teratology of the Ministry of Education and Department of Physiology, School of Basic Medical Sciences, Cheeloo College of Medicine, Shandong University, Jinan, Shandong 250012, China

* Correspondence: yshen@sdu.edu.cn; Tel.:+86-531-8838-2108

Table of Contents

1. Molecular cloning and mutant construction 4

1.1 Construction flow chart for *rif-orf5* gene knock-out mutant (Figure S1) 4

1.2 Construction and verification of *rif-orf5* gene knock-out vector pOJ260-orf5 (Figure S2) 5

1.3 Verification of *rif-orf5* gene knock-out mutant Δ*rif-orf5* (Figure S3) 5

1.4 Construction and verification of *rif-orf5* gene complementation vector pSET152-orf5 (Figure S4) 6

1.5 Verification of *rif-orf5* gene complementation mutant Δ*rif-orf5::orf5* (Figure S5) 6

1.6 HPLC analysis of the metabolites of *A. mediterranei* S699 Δ*rif-orf5* and Δ*rif-orf5::orf5* (Figure S6) 7

1.7 Structures of known compounds isolated from Δ*rif-orf5* (Figure S7) 7

1.8 Primers used in this study (Table S1) 8

2. NMR data of compounds **1–7** 8

Table S2. NMR spectroscopic data of **1a** in CD_3OD (δ in ppm, *J* in Hz) 8

Table S3. NMR spectroscopic data of **1b** in CD_3OD (δ in ppm, *J* in Hz) 9

Table S4. NMR spectroscopic data of **2a** in CD_3OD (δ in ppm, *J* in Hz) 11

Table S5. NMR spectroscopic data of **2b** in CD_3OD (δ in ppm, *J* in Hz) 12

Table S6. NMR spectroscopic data of **3a** in CD_3OD (δ in ppm, *J* in Hz) 13

Table S7. NMR spectroscopic data of **3b** in CD_3OD (δ in ppm, *J* in Hz) 15

Table S8. NMR spectroscopic data of **4** in CD_3OD (δ in ppm, *J* in Hz) 16

Table S9. NMR spectroscopic data of **5** in CD_3OD (δ in ppm, *J* in Hz) 17

Table S10. NMR spectroscopic data of **6** in CD_3OD (δ in ppm, *J* in Hz) 19

Table S11. NMR spectroscopic data of **7** in CD_3OD (δ in ppm, *J* in Hz) 20

Table S12. NMR spectroscopic data of **12** in CD_3OD (δ in ppm, *J* in Hz) 21

Table S13. Selected ^1H NMR spectroscopic data for the hemiacetals of compounds **1–3** and **9**, **10** 23

3. NMR and HRESIMS spectra of compounds **1–7** 23

Figures S8–14. NMR and HRESIMS spectra of **1** 23

Figures S15–21. NMR and HRESIMS spectra of **2** 27

Figures S22–28. NMR and HRESIMS spectra of **3** 30

Figures S29–34. NMR and HRESIMS spectra of **4** 34

Figures S35–41. NMR and HRESIMS spectra of **5** 37
Figures S42–48. NMR and HRESIMS spectra of **6** 40
Figures S49–55. NMR and HRESIMS spectra of **7** 44
4 Bioactivity 47
4.1 Antimicrobial activity of compounds **1–13** (Figure S56) 47
Table S14. Diameters of the inhibition zones and MICs of compounds **1–3** against *Staphylococcus aureus* ATCC 25923 48
4.2 Cytotoxicity assay 48
TableS15. Antiproliferative activity against HeLa and Caco-2 cells of compounds **1–13** 48
Figure S57. Antiproliferative activity against HeLa cells of compounds **1–13** (50 and 10 μM, respectively). 49
Figure S58. Antiproliferative activity against Caco-2 cells of compounds **1–13** (50 and 10 μM, respectively). 50

1. Molecular cloning and mutant construction

1.1. Construction flow chart for *rif-orf5* gene knock-out mutant (Figure S1)

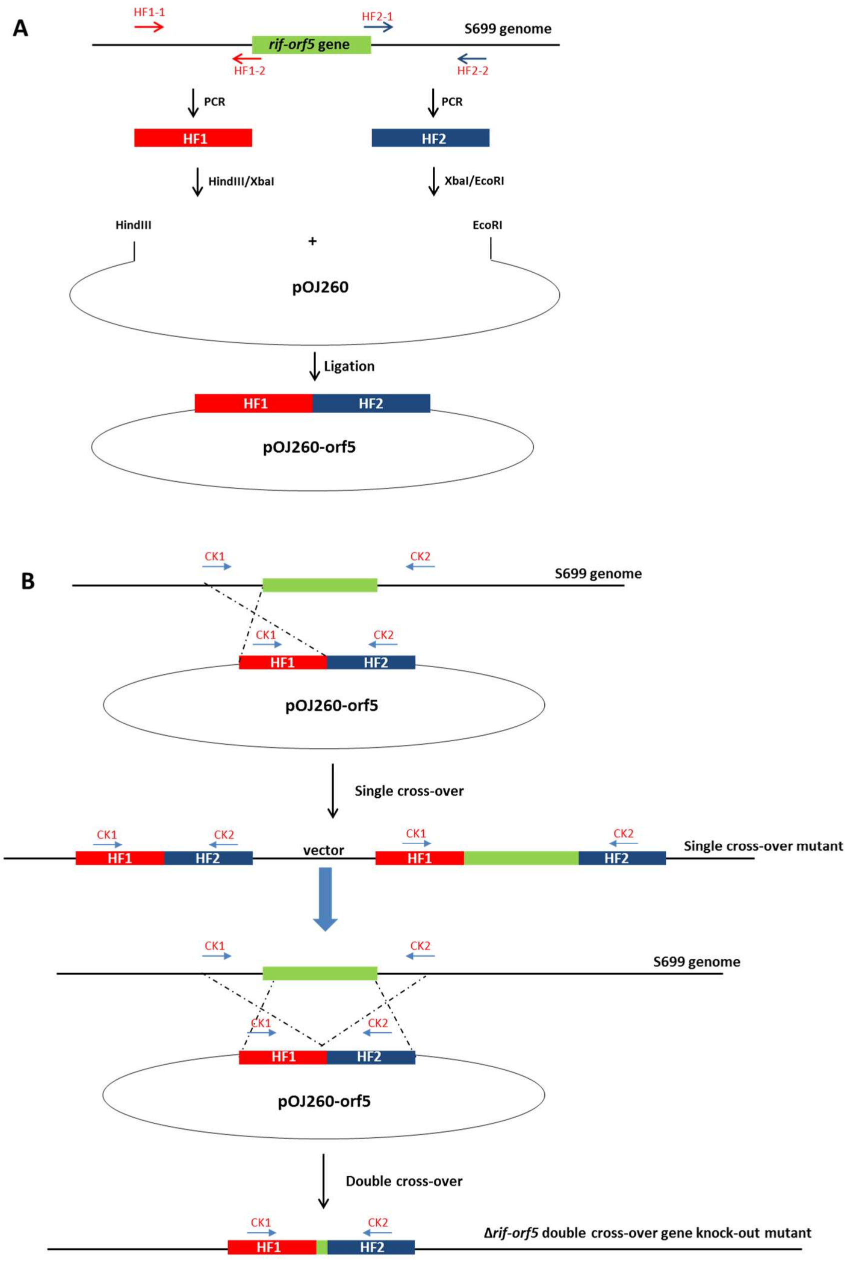

Figure S1. Construction flow chart for *rif-orf5* gene knock-out mutant Δ*rif-orf5*. (A) Construction flow chart of *rif-orf5* gene knock-out vector pOJ260-orf5 through ligation; (B) construction flow chart of *rif-orf5* gene knock-out mutant Δ*rif-orf5* through homologous recombination.

1.2. Construction and verification of *rif-orf5* gene knock-out vector pOJ260-orf5 (Figure S2)

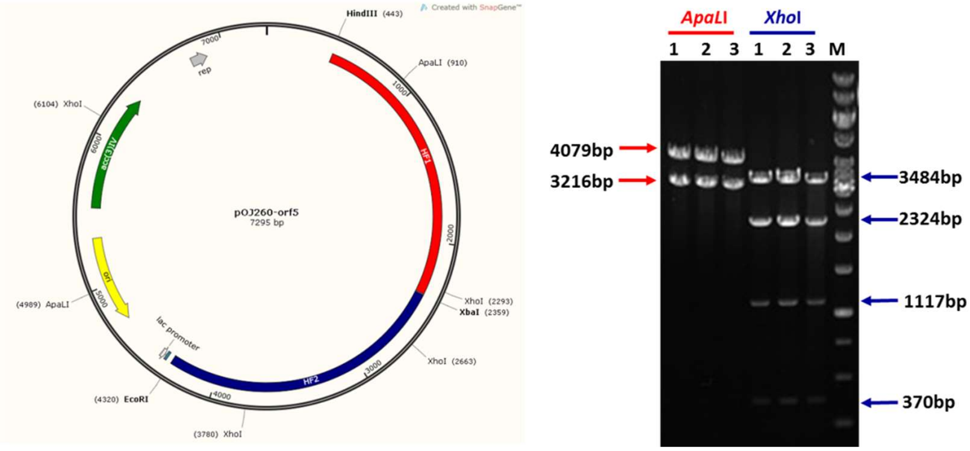

Figure S2. Construction and enzymatic digestion verification of pOJ260-orf5, which was verified by sequencing.

1.3. Verification of *rif-orf5* gene knock-out mutant Δ*rif-orf5* (Figure S3)

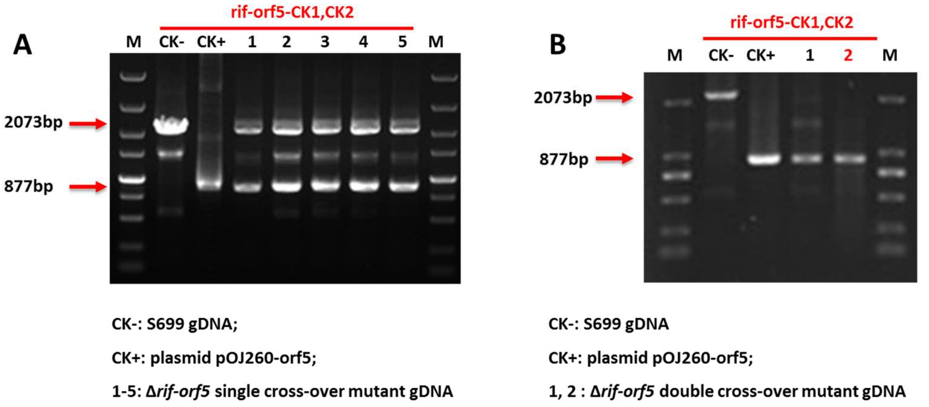

Figure S3. PCR verification of *rif-orf5* gene knock-out mutant Δ*rif-orf5*. (A) PCR verification of single crossover mutant; (B) PCR verification of double crossover mutant Δ*rif-orf5*.

1.4. Construction and verification of *rif-orf5* gene complementation vector pSET152-orf5 (Figure S4)

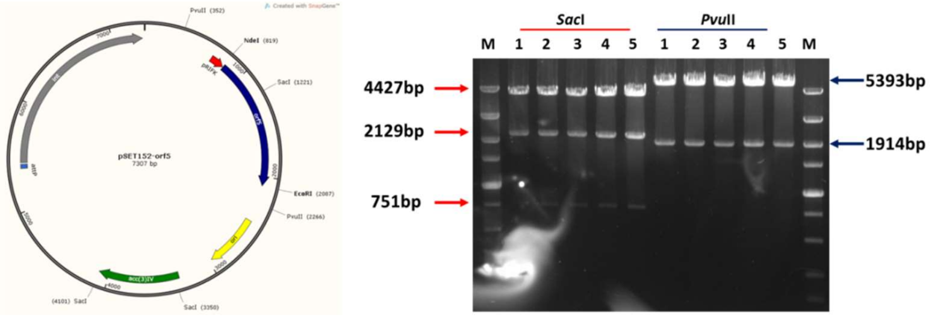

Figure S4. Construction and enzymatic digestion verification of pSET152-orf5, which was verified by sequencing.

1.5. Verification of *rif-orf5* gene complementation mutant Δ*rif-orf5::orf5* (Figure S5)

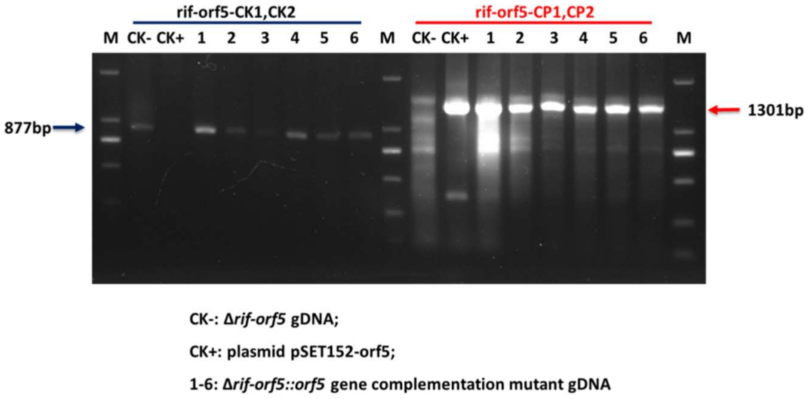

Figure S5. PCR verification of *rif-orf5* gene complementation mutant Δ*rif-orf5::orf5*.

1.6. HPLC analysis of the metabolites of *A. mediterranei* S699 Δ*rif-orf5* and Δ*rif-orf5::orf5* (Figure S6)

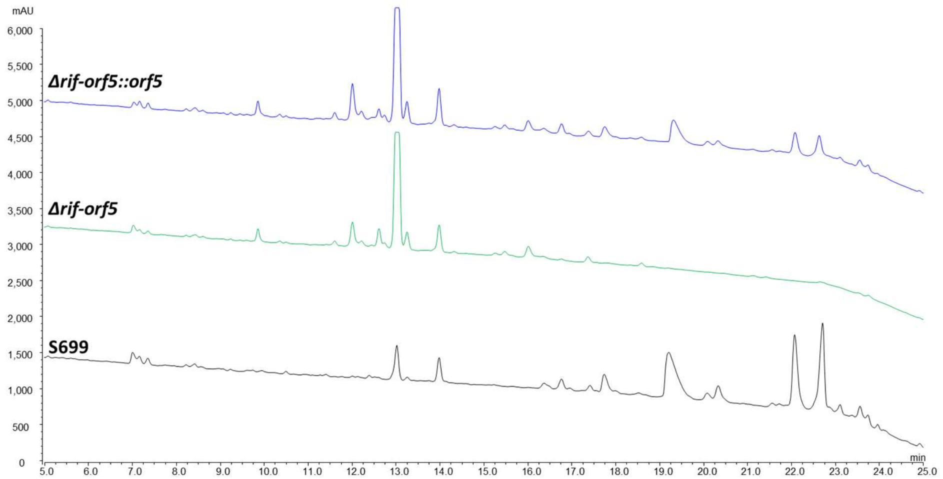

Figure S6. HPLC detection of *rif-orf5* gene knock-out and gene complementation mutants.

1.7. Structures of known compounds isolated from Δ*rif-orf5* (Figure S7)

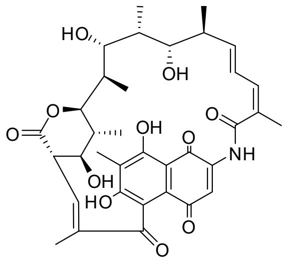

8 (rifamycin Z)

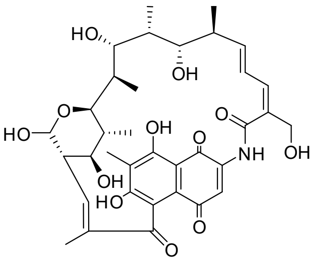

9 (30-hydroxyrifamycin W-hemiacetal)

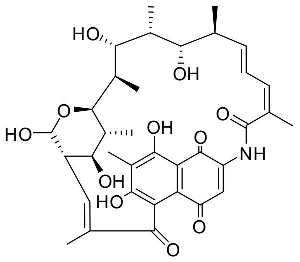

10 (rifamycin W-hemiacetal)

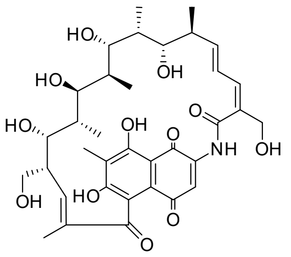

11 (30-hydroxyrifamycin W)

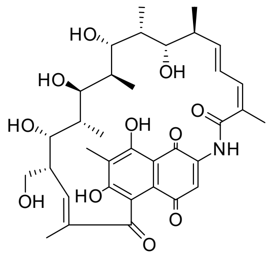

12 (rifamycin W)

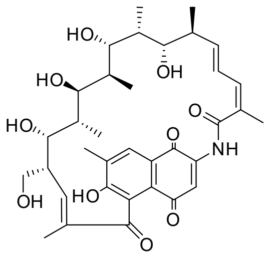

13 (protorifamycin I)

Figure S7. Structures of known compounds.

1.8. Primers used in this study (Table S1)

Table S1. Primers used in this study.

Primer	Oligonucleotide sequences (5′ to 3′)
rif-orf5-HF1-1	CC**AAGCTT**TGTTCACCGGCATGGTC
rif-orf5-HF1-2	GC**TCTAGA**ACCCGGACCAAGGACCT
rif-orf5-HF2-1	GC**TCTAGA**GGCAGTGGTGGTCATGG
rif-orf5-HF2-2	CG**GAATTC**GGAGTAGGAGGTGACGC
rif-orf5-CK1	TGATGACCTTCTGCTGGACG
rif-orf5-CK2	TCAAGAGCACCGACATCCAC
rif-orf5-CP1	GCGGAGATTCGGAGACATATGACCACCACTGCCGAGA
rif-orf5-CP2	GACATGATTACGAATTCTCAGGACGCCGGCCGGGCG

2. NMR data of compounds **1–7**

Table S2. NMR spectroscopic data of **1a** in CD_3OD (δ in ppm, J in Hz).

No.	δ_H (mult)	δ_C mult	HMBC	^1H-^1H COSY
1		184.7s		
2		143.0s		
3	7.64 (s)	117.3d	C-1, C-2, C-10	
4		187.1s		
5	7.18 (s)	103.7d	C-7	
6		165.9s		
6a	4.00 (s)	57.0q	C-6	
7		120.3s		
8		161.7s		
9		131.7s		
10		132.6s		
11		172.3s		
12		133.3s		

13	1.89 (s)	13.9q	C-11, C-12, C-29	
14	2.09 (s)	8.3q	C-6, C-7, C-8	
15		170.0s		
16		129.3s		
17	6.50 (d, 10.8)	139.0d	C-15, C-30	H-18
18	6.87 (dd, 11.2, 14.9)	127.6d		H-17, H-19
19	6.08 (m)	146.3d	C-17	H-18, H-20
20	2.44 (m)	42.5d		H-19, H-21, H-31
21	3.82 (m)	75.8d	C-31	H-20, H-22
22	2.04 (m)	36.6d		H-23, H-32
23	3.62 (m)	78.5d		H-22, H-24
24	1.98 (m)	37.0d		H-23, H-33
25	3.55 (m)	73.4d		H-24, H-26
26	1.59 (m)	40.3d		H-25, H-27, H-34
27	3.18 (t, 9.8)	77.5d		H-26, H-28
28	2.42 (m)	53.9d	C-34a	H-27, H-29, H-34a
29	6.62 (d, 10.7)	141.0d	C-11	H-28
30	2.08 (s)	20.7q	C-15, C-16, C-17	
31	1.02 (s)	17.3q	C-19, C-20, C-21	H-20
32	1.00 (s)	11.2q	C-21, C-22, C-23	H-22
33	0.98 (s)	10.5q	C-22, C-23, C-24	H-24
34	0.96 (s)	12.9q	C-25, C-26, C-27	H-26
34a	4.54 (d, 8.4)	98.2d		H-28

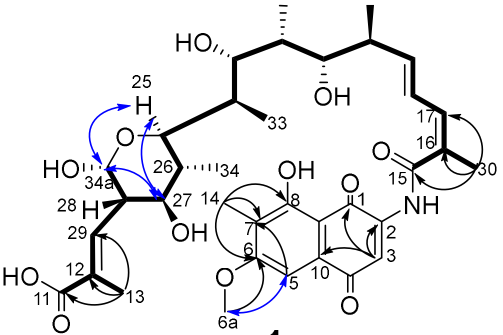

1a

Table S3. NMR spectroscopic data of **1b** in CD_3OD (δ in ppm, *J* in Hz).

No	δ_H (mult)	δ_C mult	HMBC	^1H-^1H COSY
1		184.7s		
2		143.0s		

3	7.64 (s)	117.3d	C-1, C-2, C-10	
4		187.1s		
5	7.18 (s)	103.7d	C-7	
6		165.9s		
6a	4.00 (s)	57.0q	C-6	
7		120.3s		
8		161.7s		
9		131.7s		
10		132.6s		
11		172.3s		
12		132.4s		
13	1.87 (s)	13.3q	C-11, C-12, C-29	
14	2.09 (s)	8.3q	C-6, C-7, C-8	
15		170.0s		
16		129.3s		
17	6.50 (d, 10.8)	139.0d	C-15, C-30	H-18
18	6.87 (dd, 11.2, 14.9)	127.6d		H-17, H-19
19	6.08 (m)	146.3d	C-17	H-18, H-20
20	2.44 (m)	42.5d		H-19,H-21, H-31
21	3.82 (m)	75.8d	C-31	H-20, H-22
22	2.04 (m)	36.6d		H-23, H-32
23	3.62 (m)	78.5d		H-22, H-24
24	1.98 (m)	37.0d		H-23, H-33
25	4.21 (d, 10.4)	72.8d		H-26
26	1.63 (m)	41.3d		H-25, H-27, H-34
27	3.55 (m)	73.4d		H-26, H-28
28	2.64 (dt, 3.5, 10.0)	50.7d		H-27, H-29, H-34a
29	6.79 (d, 10.1)	142.4d	C-11	H-28
30	2.08 (s)	20.7q	C-15, C-16, C-17	
31	1.02 (s)	17.3q	C-19, C-20, C-21	H-20
32	1.00 (s)	11.2q	C-21, C-22, C-23	H-22
33	0.98 (s)	10.5q	C-22, C-23, C-24	H-24
34	0.96 (s)	12.9q	C-25, C-26, C-27	H-26
34a	5.08 (d, 3.2)	94.5d		H-28

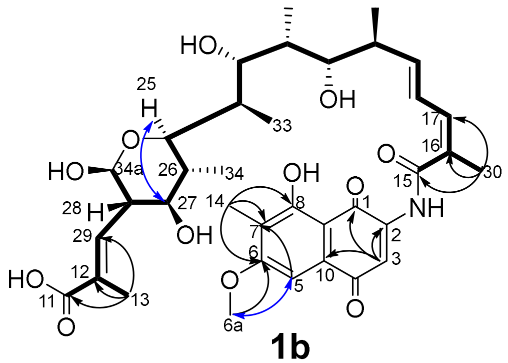

1b

Table S4. NMR spectroscopic data of **2a** in CD_3OD (δ in ppm, J in Hz).

No	δ_H (mult)	δ_C mult	HMBC	^{1}H-^{1}H COSY
1		184.1s		
2		143.2s		
3	7.64 (s)	117.2d	C-1, C-2, C-10	
4		186.8s		
5	6.99 (s)	108.6d	C-1	
6		163.9s		
7		118.2s		
8		165.0s		
9		130.6s		
10		132.3s		
11		171.8s		
12		133.1s		
13	1.90 (s)	13.9q	C-11, C-12, C-29	
14	2.07 (s)	8.2q	C-6, C-7, C-8	
15		169.0s		
16		142.6s		
17	6.64 (d, 11.3)	143.7d	C-15, C-19, C-30	H-18
18	7.21 (dd, 11.1, 15.0)	128.1d	C-20	H-17, H-19
19	6.23 (m)	150.1d	C-17	H-18, H-20
20	2.52 (m)	42.7d	C-19, C-21	H-19, H-21, H-31
21	3.84 (d, 8.7)	75.8d	C-32	H-20
22	1.95 (m)	36.7d	C-33	H-23
23	3.64 (m)	78.5d	C-33	H-24
24	2.03 (m)	37.1d	C-23	H-23, H-33
25	3.56 (m)	73.3d	C-34	H-25

26	1.60 (m)	40.3d		H-25, H-27, H-34
27	3.20 (t, 9.8)	77.1d	C-29, C-34	H-26, H-28
28	2.47 (m)	53.9d	C-12, C-27, C-29, C-34a	H-27, H-29, H-34a
29	6.61 (d, 11.3)	141.2d	C-11, C-13	H-28
30	4.34/4.33 (s)	66.0t	C-15, C-16, C-17	
31	1.03 (d, 7.0)	17.2q	C-19, C-20, C-21	H-20
32	1.01 (d, 7.1)	11.2q	C-21, C-22, C-23	H-22
33	0.98 (d, 6.7)	10.7q	C-22, C-23, C-24	H-24
34	0.97 (d, 6.9)	12.8q	C-25, C-26, C-27	H-26
34a	4.56 (d, 6.9)	98.0d	C-28	H-28

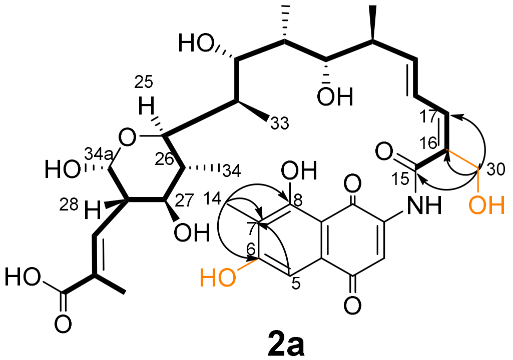

2a

Table S5. NMR spectroscopic data of **2b** in CD_3OD (δ in ppm, J in Hz).

No	δ_H (mult)	δ_C mult	HMBC	^1H-^1H COSY
1		184.1s		
2		143.2s		
3	7.64 (s)	117.2d	C-1, C-2, C-10	
4		186.8s		
5	6.99 (s)	108.6d	C-1	
6		163.9s		
7		118.2s		
8		165.0s		
9		130.6s		
10		132.3s		
11		171.8s		
12		131.5s		
13	1.88 (s)	13.3q	C-11, C-12, C-29	
14	2.07 (s)	8.2q	C-6, C-7, C-8	
15		169.0s		

16		142.6s		
17	6.64 (d, 11.3)	143.7d	C-15, C-19, C-30	H-18
18	7.21 (dd, 11.1, 15.0)	128.1d	C-20	H-17, H-19
19	6.23 (m)	150.1d	C-17	H-18, H-20
20	2.52 (m)	42.7d	C-19, C-21	H-19, H-21, H-31
21	3.84 (d, 8.7)	75.8d	C-32	H-20
22	1.95 (m)	36.7d	C-33	H-23
23	3.64 (m)	78.5d	C-33	H-24
24	2.03 (m)	37.1d	C-23	H-23, H-33
25	4.22 (d, 10.3)	72.8d		H-26
26	1.62 (m)	41.3d		H-25, H-27, H-34
27	3.56 (m)	73.3d	C-34	H-26, H-28
28	2.65 (dt, 3.5, 10.2)	50.7d	C-29	H-27, H-29, H-34a
29	6.80 (dd, 1.0, 10.0)	142.7d	C-11, C-13	H-28
30	4.34/4.33 (s)	66.0t	C-15, C-16, C-17	
31	1.03 (d, 7.0)	17.2q	C-19, C-20, C-21	H-20
32	1.01 (d, 7.1)	11.2q	C-21, C-22, C-23	H-22
33	0.98 (d, 6.7)	10.7q	C-22, C-23, C-24	H-24
34	0.97 (d, 6.9)	12.8q	C-25, C-26, C-27	H-26
34a	5.09 (d, 3.3)	94.6d	C-25	H-28

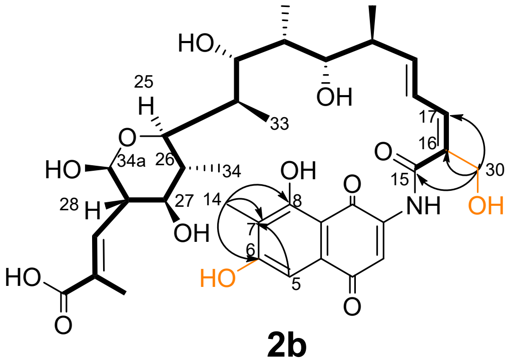

2b

Table S6. NMR spectroscopic data of **3a** in CD_3OD (δ in ppm, J in Hz).

No	δ_H (mult)	δ_C mult	HMBC	^1H-^1H COSY
1		184.1s		
2		142.4s		
3	7.61 (s)	117.2d	C-1, C-2, C-10	
4		186.5s		
5	7.02 (s)	108.8d	C-1	

6		164.0s		
7		118.3s		
8		165.2s		
9		131.1s		
10		132.4s		
11		172.3s		
12		132.4s		
13	1.90 (s)	13.9q	C-11, C-12, C-29	
14	2.09 (s)	8.2q	C-6, C-7, C-8	
15		170.0s		
16		129.4s		
17	6.50 (d, 10.8)	139.1d	C-15, C-30	H-18
18	6.86 (dd, 12.4, 14.9)	127.8d		H-17, H-19
19	6.07 (m)	146.4d	C-17	H-18, H-20
20	2.47 (m)	42.5d		H-19, H-21, H-31
21	3.83 (m)	75.8d	C-31	H-20
22	1.96 (m)	36.5d		H-23
23	3.62 (m)	78.5d		H-22, H-24
24	2.02 (m)	37.0d		H-23
25	3.56 (m)	73.4d		H-24, H-26
26	1.60 (m)	40.4d		H-27
27	3.17 (m)	77.1d		H-26, H-28
28	2.42 (m)	53.9d	C-34a	H-27, H-29, H-34a
29	6.62 (d, 10.7)	141.1d		H-28
30	2.08 (s)	20.7q	C-15, C-16, C-17	
31	1.02 (s)	17.2q	C-19, C-20, C-21	H-20
32	1.00 (s)	11.2q	C-21, C-22, C-23	H-22
33	0.98 (s)	10.9q	C-22, C-23, C-24	H-24
34	0.96 (s)	12.8q	C-25, C-26, C-27	H-26
34a	4.54 (d, 6.9)	98.0d		H-28

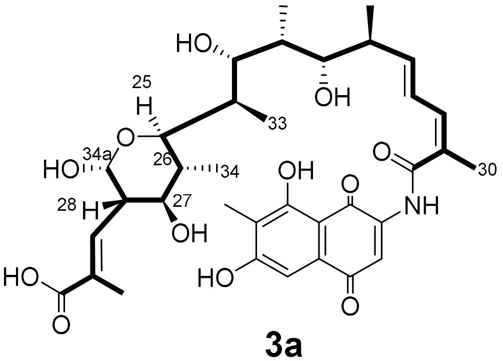

3a

Table S7. NMR spectroscopic data of **3b** in CD_3OD (δ in ppm, J in Hz).

No	δ_H (mult)	δ_C mult	HMBC	^1H-^1H COSY
1		184.1s		
2		142.4s		
3	7.61 (s)	117.2d	C-1, C-2, C-10	
4		186.5s		
5	7.02 (s)	108.8d	C-1	
6		164.0s		
7		118.3s		
8		165.2s		
9		131.1s		
10		132.4s		
11		172.3s		
12		131.1s		
13	1.88 (s)	13.3q	C-11, C-12, C-29	
14	2.09 (s)	8.2q	C-6, C-7, C-8	
15		170.0s		
16		129.4s		
17	6.50 (d, 10.8)	139.1d	C-15, C-30	H-18
18	6.86 (dd, 12.4, 14.9)	127.8d		H-17, H-19
19	6.07 (m)	146.4d	C-17	H-18, H-20
20	2.47 (m)	42.5d		H-19,H-21, H-31
21	3.83 (m)	75.8d	C-31	H-20
22	1.96 (m)	36.5d		H-23
23	3.62 (m)	78.5d		H-22, H-24
24	2.02 (m)	37.0d		H-23
25	4.21 (m)	72.7d	C-33	H-26
26	1.61 (m)	41.3d		H-25
27	3.56 (m)	73.4d		H-26, H-28
28	2.61 (m)	50.7d		H-27, H-29, H-34a
29	6.78 (d, 10.1)	142.4d		H-28
30	2.08 (s)	20.7q	C-15, C-16, C-17	
31	1.02 (s)	17.2q	C-19, C-20, C-21	H-20
32	1.00 (s)	11.2q	C-21, C-22, C-23	H-22
33	0.98 (s)	10.9q	C-22, C-23, C-24	H-24
34	0.96 (s)	12.8q	C-25, C-26, C-27	H-26
34a	5.08 (d, 3.3)	94.5d		H-28

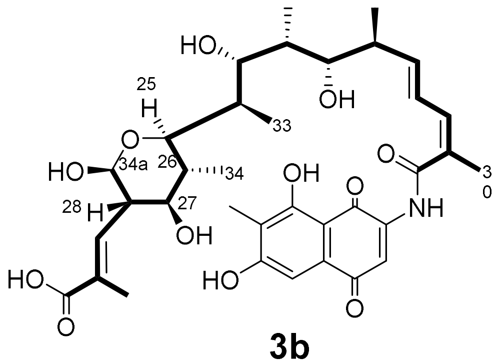

3b

Table S8. NMR spectroscopic data of **4** in CD_3OD (δ in ppm, *J* in Hz).

No	δ_H (mult)	δ_C mult	HMBC	^1H-^1H COSY
1		183.7s		
2		142.7s		
3	7.65 (s)	117.2d	C-1, C-2, C-10	
4		186.8s		
5	7.07 (s)	108.9d	C-4, C-7, C-9	
6		164.0s		
7		118.4s		
8		165.6s		
9		126.1s		
10		132.4s		
11		173.0s		
12		131.8s		
13	1.89 (s)	13.9q	C-11, C-12, C-29	
14	2.12 (s)	8.1q	C-6, C-7, C-8	
15		170.1s		
16		129.5s		
17	6.53 (t, 14.3)	138.9d	C-15	H-18, H-30
18	6.84 (dd, 10.9, 14.3)	127.6d		H-17, H-19
19	6.08 (dd, 8.2, 15.0)	146.5d	C-17	H-18, H-20
20	2.45 (m)	42.5d	C-19, C-21, C-31	H-19,H-21, H-31
21	3.82 (d, 8.8)	75.8d	C-32	H-20, H-22
22	1.90 (m)	36.9d		H-23, H-32
23	3.60 (m)	79.3 d		H-22, H-24
24	1.84 (m)	36.7d		H-23, H-25, H-33
25	4.05 (d, 9.7)	73.3d	C-34	H-26

26	1.80 (m)	40.9d		H-25, H-27, H-34
27	4.13 (d, 5.5)	72.1d	C-34a	H-26, H-28
28	2.82 (m)	47.0d	C-34a	H-27, H-29, H-34a
29	6.91 (d, 10.4)	142.7d		H-13, H-28
30	2.09 (s)	20.7q	C-15, C-16, C-17	
31	1.00 (d, 6.8)	17.5q	C-19, C-20, C-21	H-20
32	0.90 (d, 6.8)	10.5q	C-21, C-22, C-23	H-22
33	0.96 (d, 6.8)	10.7q	C-22, C-23, C-24	H-24
34	0.83 (d, 6.8)	10.8q	C-25, C-26, C-27	H-26
34a	3.62 (m)<br>3.54 (m)	64.6t	C-27	H-28

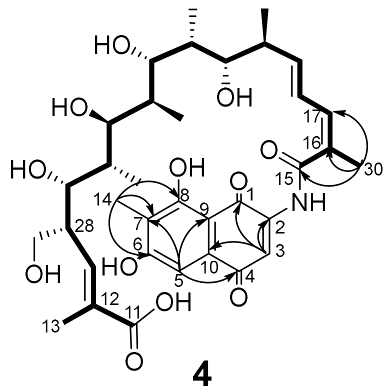

4

Table S9. NMR spectroscopic data of **5** in CD_3OD (δ in ppm, *J* in Hz).

No	δ_H (mult)	δ_C mult	HMBC	^1H-^1H COSY
1		184.1s		
2		142.4s		
3	7.57 (s)	118.6d	C-1, C-2, C-10	
4		186.6s		
5		108.3s		
6		161.8s		
7		119.2s		
8		164.0s		
9		125.5s		
10		129.9s		
11		200.1s		
12		141.6s		
13	2.08 (s)	12.7q	C-11, C-12, C-29	

14	2.18 (s)	8.7q	C-6, C-7, C-8	
15		172.2s		
16		132.2s		
17	6.25 (d, 10.8)	135.2d	C-15, C-18, C-19, C-30	H-18, H-30
18	6.51 (dd, 11.0, 15.8)	126.3d	C-16, C-17, C-20	H-17, H-19
19	6.09 (dd, 6.6, 15.9)	141.6d	C-17, C-20, C-21, C-31	H-18, H-20
20	2.36 (m)	39.2d	C-18, C-19, C-21, C-31	H-19, H-21, H-31
21	4.03 (m)	74.9d	C-19, C-20, C-23, C-32	H-20, H-22
22	1.87 (m)	34.4d	C-23, C-32	H-32
23	3.48 (d, 10.2)	79.0d	C-21, C-22, C-24, C-32	H-22, H-24
24	1.80 (m)	38.0d	C-23, C-33	H-23, H-33
25	3.98 (m)	71.3d	C-23, C-24, C-26, C-27, C-33	H-24, H-26
26	1.43 (m)	43.9d	C-25, C-34	H-25, H-34
27	4.31 (s)	68.7d	C-25, C-26, C-28, C-29, C-34, C-34a	H-26, H-28
28	2.89 (m)	46.1d	C-12, C-29, C-34a	H-29, H-34a
29	6.30 (d, 9.3)	139.0d	C-11, C-13, C-27	H-13, H-28
30	2.09 (s)	20.3q	C-15, C-16, C-17	
31	0.91 (d, 6.9)	18.2q	C-19, C-20, C-21	H-20
32	1.05 (d, 7.0)	11.3q	C-21, C-22, C-23	H-22
33	0.72 (d, 6.8)	8.9q	C-23, C-24, C-25	H-24
34	0.40 (d, 7.0)	11.8q	C-25, C-26, C-27	H-26
34a	4.01 (m)<br>4.00 (m)	65.8t	C-27, C-29, C-36	H-28
AcO-34a	2.03 (s)	21.0q<br>172.9s		

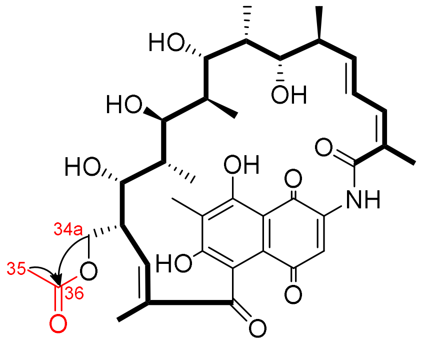

5

Table S10. NMR spectroscopic data of **6** in CD_3OD (δ in ppm, J in Hz).

No	δ_H (mult)	δ_C mult	HMBC	^1H-^1H COSY
1		184.0s		
2		142.4s		
3	7.56 (s)	119.1d	C-1, C-2, C-10	
4		186.9s		
5		108.1s		
6		163.6s		
7		119.3s		
8		164.0s		
9		124.7s		
10		130.8s		
11		200.3s		
12		142.0s		
13	2.04 (s)	13.0q	C-11, C-12, C-29	
14	2.17 (s)	8.7q	C-6, C-7, C-8	
15		172.8s		
16		133.1s		
17	6.24 (d, 10.8)	133.4d	C-15, C-30	H-18, H-30
18	6.09 (dd, 11.0, 15.1)	127.3d	C-16, C-20	H-17, H-19
19	5.85 (dd, 9.6, 15.2)	140.7d	C-17, C-30	H-18, H-20
20	1.89 (m)	43.4d	C-19	H-19, H-31
21	3.61 (dd, 1.5, 9.2)	78.3d	C-19, C-31	H-20, H-22
22	2.86 (dd, 6.8, 9.2)	49.7d	C-21, C-32	H-21, H-32
23		211.3s		
24	2.52 (m)	49.9d	C-33	H-33
25	3.87 (d, 10.2)	71.2d	C-26, C-27, C-33	H-24, H-26
26	1.35 (m)	42.7d	C-25	H-25, H-34
27	4.43 (s)	68.3d	C-25, C-26, C-28, C-29, C-34, C-34a	H-26, H-28
28	2.58 (m)	49.3d	C-29, C-34a	H-29, H-34a
29	6.28 (d, 9.1)	140.2d	C-11, C-13	H-28
30	2.05 (s)	20.4q	C-15, C-17	
31	1.06 (d, 3.1)	20.2q	C-19, C-20, C-21	H-20
32	1.05 (d, 3.2)	14.8q	C-21, C-22	H-22
33	1.12 (d, 7.4)	8.4q	C-24, C-25	H-24
34	0.41 (d, 7.0)	11.9q	C-25, C-26, C-27	H-26

34a	3.52 (dd, 8.6, 10.9)	64.4t	C-27, C-29	H-28
	3.38 (dd, 6.1, 11.0)			

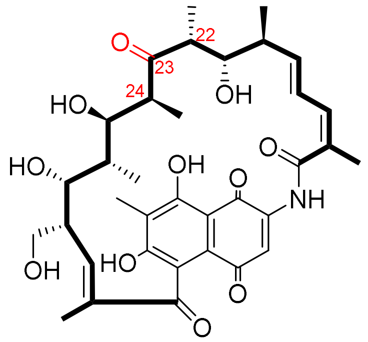

6

Table S11. NMR spectroscopic data of **7** in CD_3OD (δ in ppm, J in Hz).

No	δ_H (mult)	δ_C mult	HMBC	^{1}H-^{1}H COSY
1		184.7s		
2		143.0s		
3	7.57 (s)	119.2d	C-1, C-2, C-10	
4		187.5s		
5		108.7s		
6		163.1s		
7		119.2s		
8		164.7s		
9		126.0s		
10		130.9s		
11		201.1s		
12		142.0s		
13	2.06 (d, 1.0)	13.4q	C-11, C-12, C-29	H-29
14	2.17 (s)	9.3q	C-6, C-7, C-8	
15		172.2s		
16		133.4s		
17	6.26 (dd, 0.8, 10.9)	136.2d	C-15, C-18, C-19, C-30	H-18
18	5.96 (d, 16.0)	136.2d	C-17, C-19, C-21	H-19
19	6.47 (dd, 10.9, 15.9)	126.1d	C-16, C-20, C-21	H-18
20		77.0s		
21	3.95 (d, 1.2)	76.7d	C-18, C-22, C-23, C-32	H-22
22	2.01 (m)	35.3d	C-32	H-23, H-32
23	3.42 (dd, 2.7, 9.4)	80.8d	C-21	H-22, H-24

24	1.72 (m)	39.0d	C-23	H-23, H-33
25	3.94 (dd, 1.9, 10.6)	72.3d	C-24, C-26, C-27, C-33	H-26
26	1.40 (m)	44.6d	C-25	H-25, H-34
27	4.37 (br. s)	70.0d	C-25, C-26, C-28, C-29, C-34, C-34a	H-26, H-28
28	2.65 (q, 7.1, 16.0)	49.1d	C-29, C-34a	H-29, H-34a
29	6.35 (dd, 1.0, 9.5)	141.5d	C-11, C-13, C-27	H-13, H-28
30	2.10 (s)	21.0q	C-15, C-16, C-17	H-17
31	1.21 (s)	26.7q	C-19, C-20, C-21	H-20
32	1.17 (d, 7.0)	14.6q	C-21, C-22, C-23	H-22
33	0.74 (d, 6.8)	9.8q	C-23, C-24, C-25	H-24
34	0.41 (d, 7.0)	12.4q	C-25, C-26, C-27	H-26
34a	3.40 (m)<br>3.58 (dd, 8.0, 10.9)	65.1t	C-27, C-28, C-29	H-28

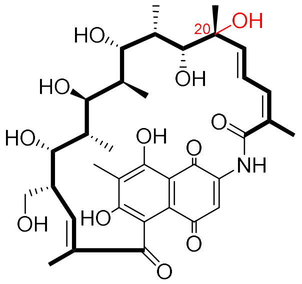

7

Table S12. NMR spectroscopic data of **12** in CD_3OD (δ in ppm, J in Hz).

No	δ_H (mult)	δ_C mult	HMBC	^1H-^1H COSY
1		185.1s		
2		143.1s		
3	7.58 (s)	119.3d	C-1, C-2, C-10	
4		187.3s		
5		109.0s		
6		162.3s		
7		120.0s		
8		164.1s		
9		125.9s		
10		130.6s		
11		201.4s		
12		141.6s		

13	2.08 (s)	13.4q	C-11, C-12, C-29	
14	2.18 (s)	9.3q	C-6, C-7, C-8	
15		172.9s		
16		132.9s		
17	6.26 (dd, 0.9, 11.0)	135.9d	C-15, C-18, C-19, C-30	H-18, H-30
18	6.52 (dd, 11.0, 15.9)	126.9d	C-16, C-17, C-20	H-17, H-19
19	6.09 (dd, 6.7, 16.0)	142.3d	C-17, C-20, C-21, C-31	H-20
20	2.36 (q, 7.1)	39.9d	C-18, C-19, C-21, C-31	H-19, H-21, H-31
21	4.04 (d, 10.5)	75.5d	C-19, C-20, C-32	H-20, H-22
22	1.87 (m)	35.1d	C-32	H-21, H-23, H-32
23	3.49 (dd, 2.1, 10.2)	80.8d	C-21, C-22, C-24, C-32	H-22, H-24
24	1.81 (m)	38.7d	C-23, C-33	H-23, H-25, H-33
25	4.01 (dd, 1.2, 10.1)	71.9d	C-21, C-23, C-24, C-26, C-27	H-24, H-26
26	1.42 (m)	38.7d	C-25, C-34	H-25, H-27, H-34
27	4.40 (s)	69.7d	C-25, C-26, C-28, C-29, C-34, C-34a	H-26, H-28
28	2.65 (dq, 1.2, 7.7)	50.5d	C-29, C-34a	H-27, H-29, H-34a
29	6.38 (dd, 1.0, 9.4)	141.7d	C-11, C-13, C-27, C-34a	H-13, H-28
30	2.11 (s)	20.9q	C-15, C-16, C-17	
31	0.92 (d, 7.0)	18.9q	C-19, C-20, C-21	H-20
32	1.06 (d, 7.1)	12.0q	C-21, C-22, C-23	H-22
33	0.73 (d, 6.8)	9.7q	C-23, C-24, C-25	H-24
34	0.42 (d, 7.0)	12.5q	C-25, C-26, C-27	H-26
34a	3.43 (dd, 6.3, 10.8)<br>3.57 (m)	65.1t	C-27, C-28, C-29	H-28

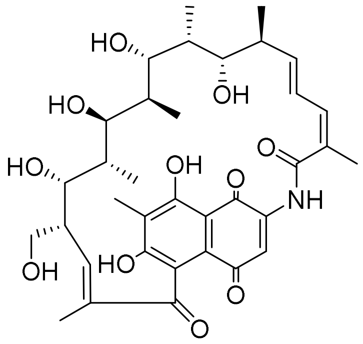

12 (rifamycin W)

Table S13. Selected ^{1}H NMR spectroscopic data for the hemiacetals of compounds **1**–**3** and **9**, **10**.

Pos.	δ_H (mult. *J* in Hz)							
	**1**		**2**		**3**		**9**	**10**
	**1a**	**1b**	**2a**	**2b**	**3a**	**3b**		
25	3.55 (m)	4.21 (d, 10.4)	3.56 (m)	4.22 (d, 10.3)	3.56 (m)	4.21 (m)	3.98 (m)	3.97 (d, 10.1)
26	1.59 (m)	1.63 (m)	1.60 (m)	1.62 (m)	1.60 (m)	1.61 (m)	1.50 (m)	1.51 (m)
27	3.18 (t, 9.8)	3.55 (m)	3.20 (t, 9.8)	3.56 (m)	3.17 (m)	3.56 (m)	3.22 (t, 10.2)	3.19 (t, 10.2)
28	2.42 (m)	2.64 (dt, 3.5, 10.0)	2.47 (m)	2.65 (dt, 3.5, 10.2)	2.42 (m)	2.61 (m)	2.51 (m)	2.54 (m)
29	6.62 (d, 10.7)	6.79 (d, 10.1)	6.61 (d, 11.3)	6.80 (d, 10.0)	6.62 (d, 10.7)	6.78 (d, 10.1)	5.81 (d, 8.3)	5.83 (d, 8.2)
34a	4.54 (d, 8.4)	5.08 (d, 3.2)	4.56 (d, 6.9)	5.09 (d, 3.3)	4.54 (d, 6.9)	5.08 (d, 3.3)	5.12 (d, 4.1)	5.10 (d, 4.1)

3. NMR and HRESIMS spectra of compounds **1**–**7**

Figures S8–14. NMR and HRESIMS spectra of **1**.

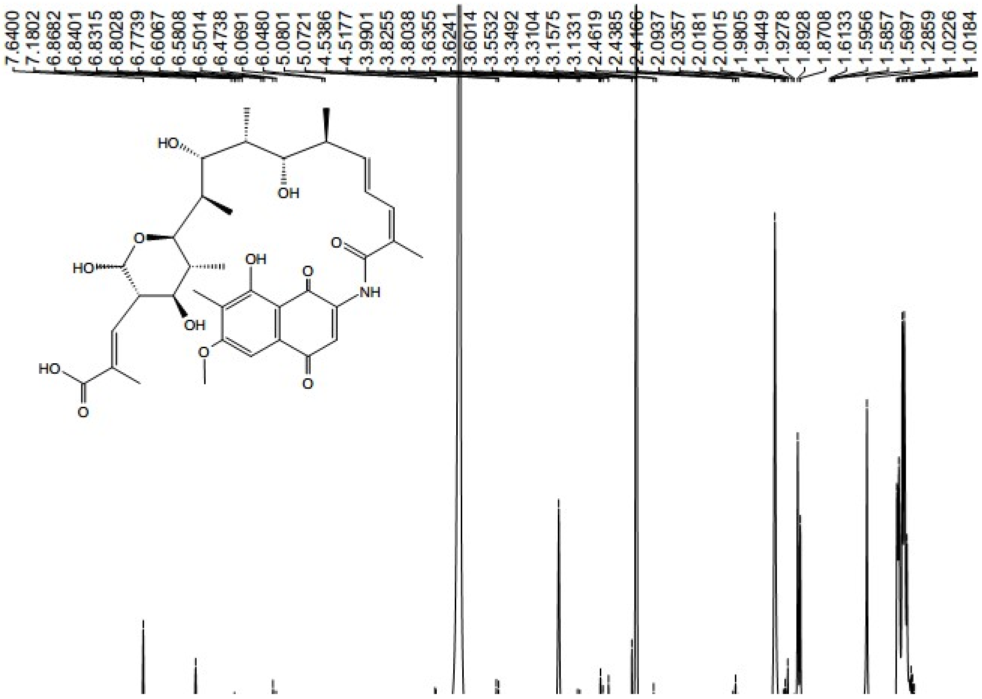

Figure S8. ^{1}H NMR spectrum of **1** in CD_3OD.

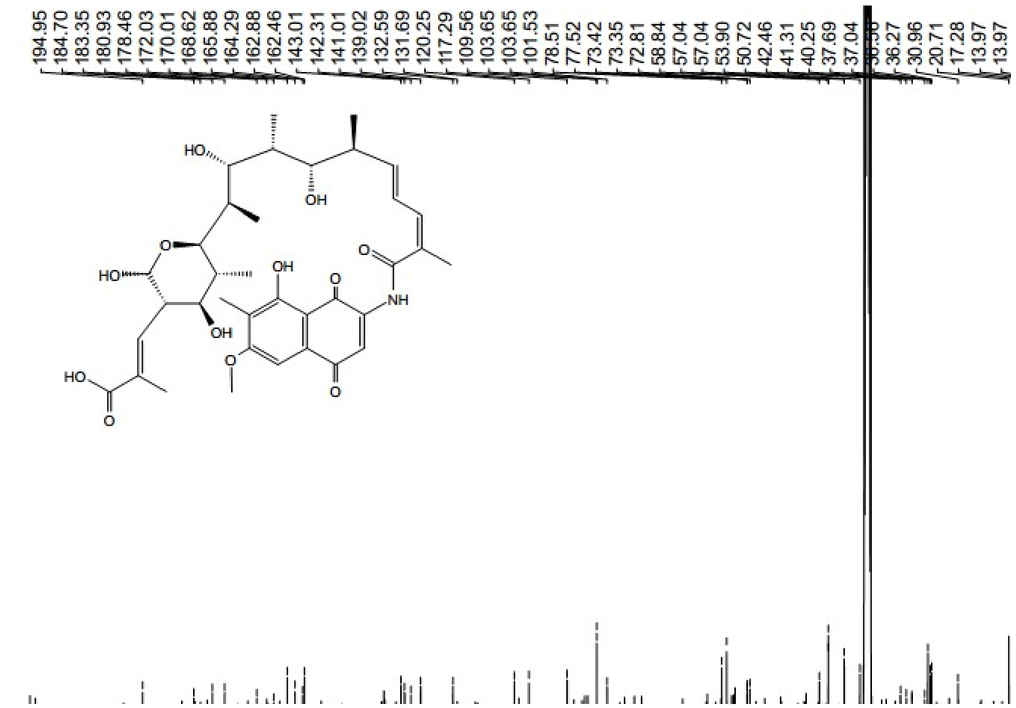

Figure S9. ^{13}C NMR spectrum of **1** in CD_3OD.

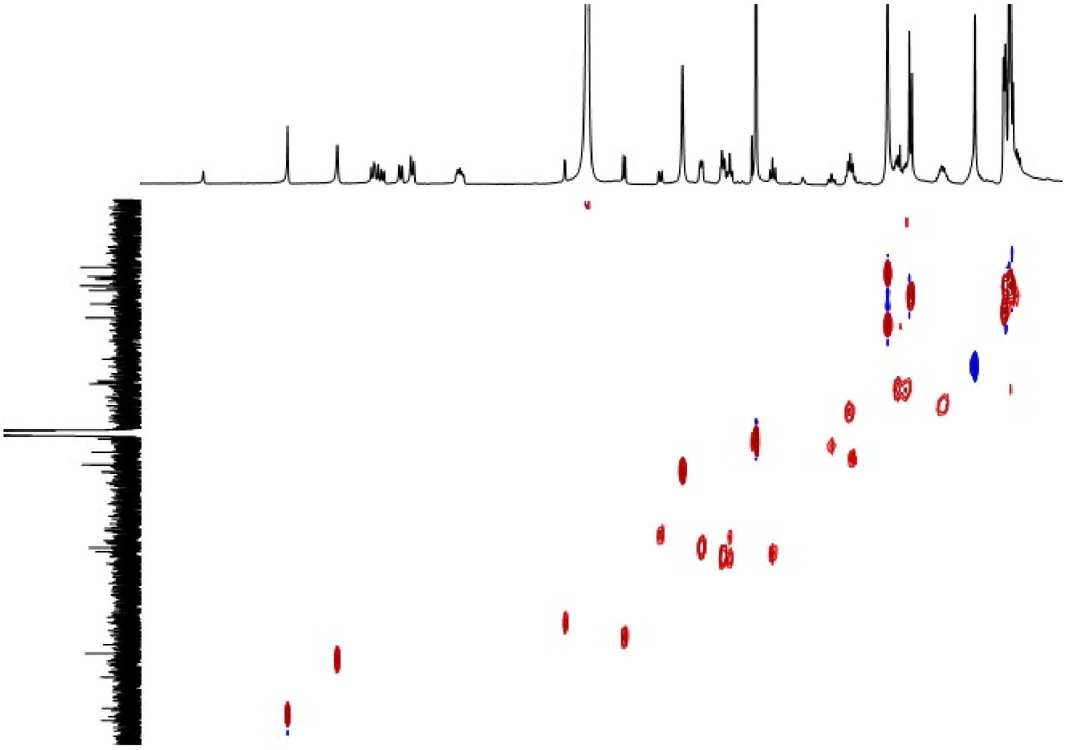

Figure S10. HSQC spectrum of **1** in CD_3OD.

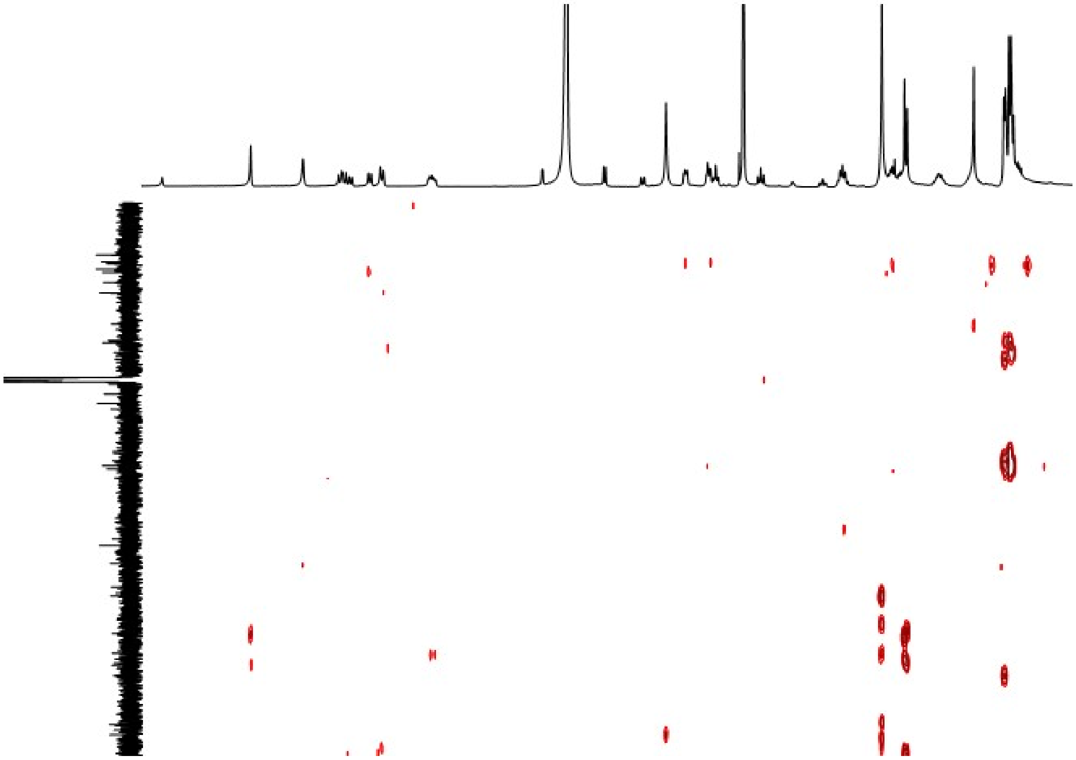

Figure S11. HMBC spectrum of **1** in CD_3OD.

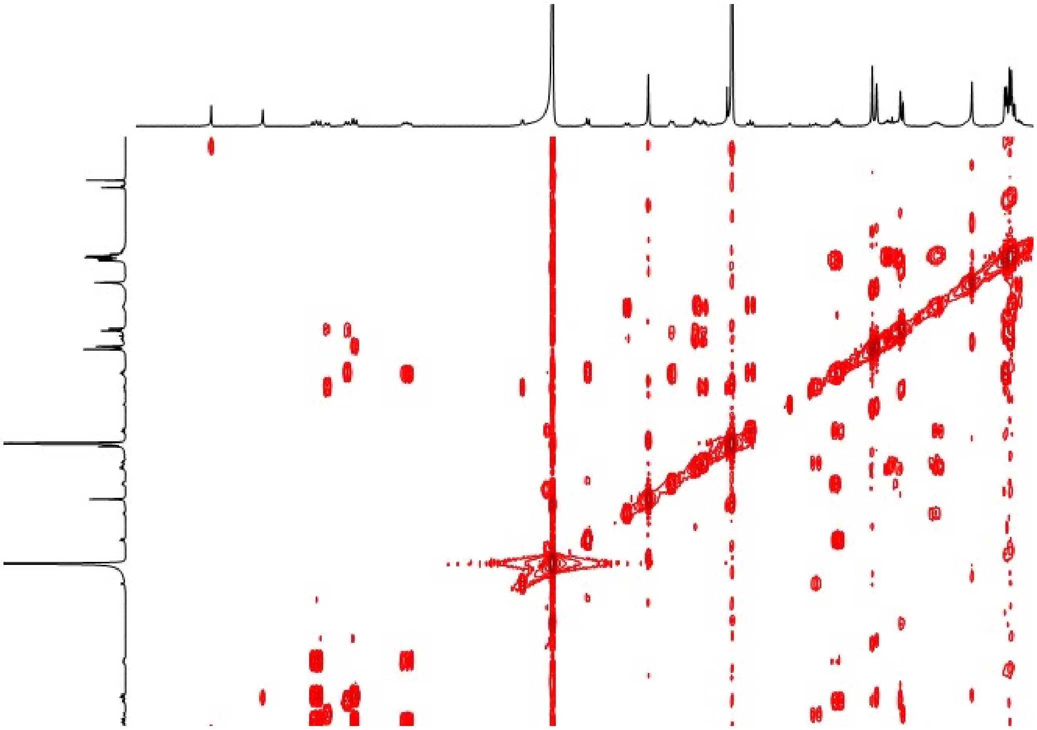

Figure S12. ^1H-^1H COSY spectrum of **1** in CD_3OD.

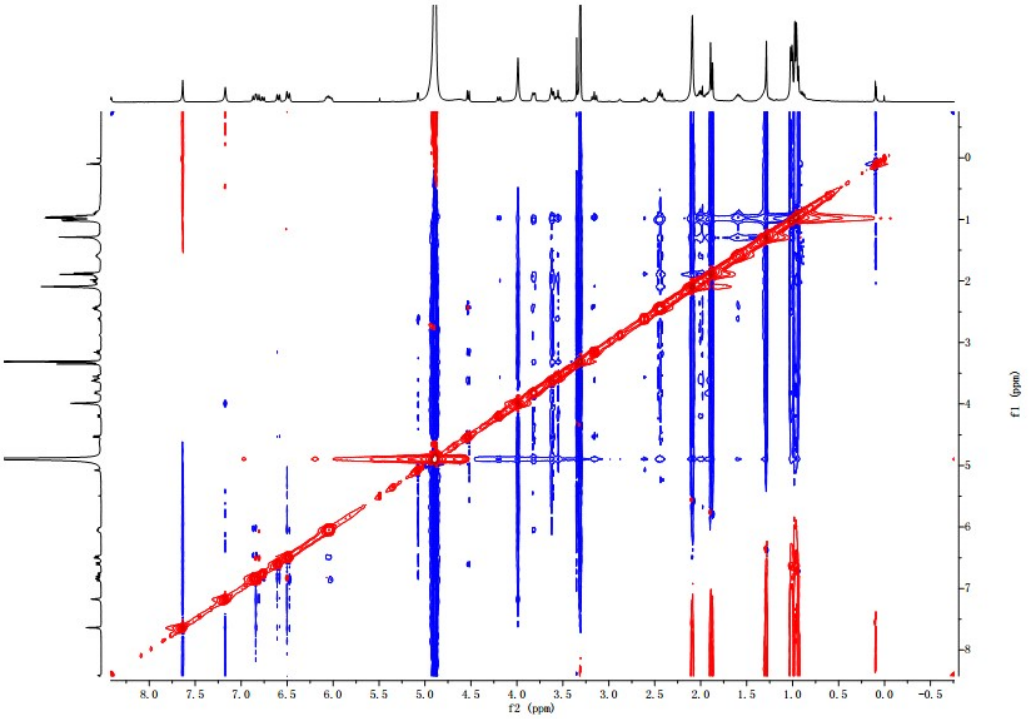

Figure S13. NOESY spectrum of **1** in CD_3OD.

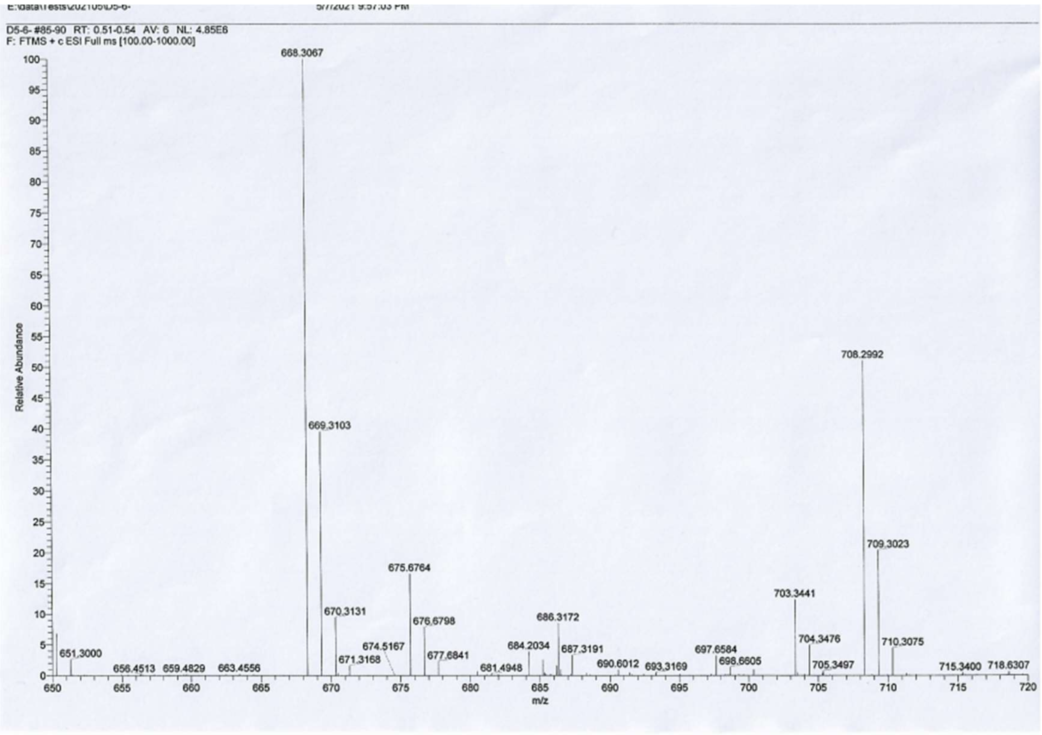

Figure S14. HRESIMS spectrum of **1**.

Figures S15–21. NMR and HRESIMS spectra of **2**.

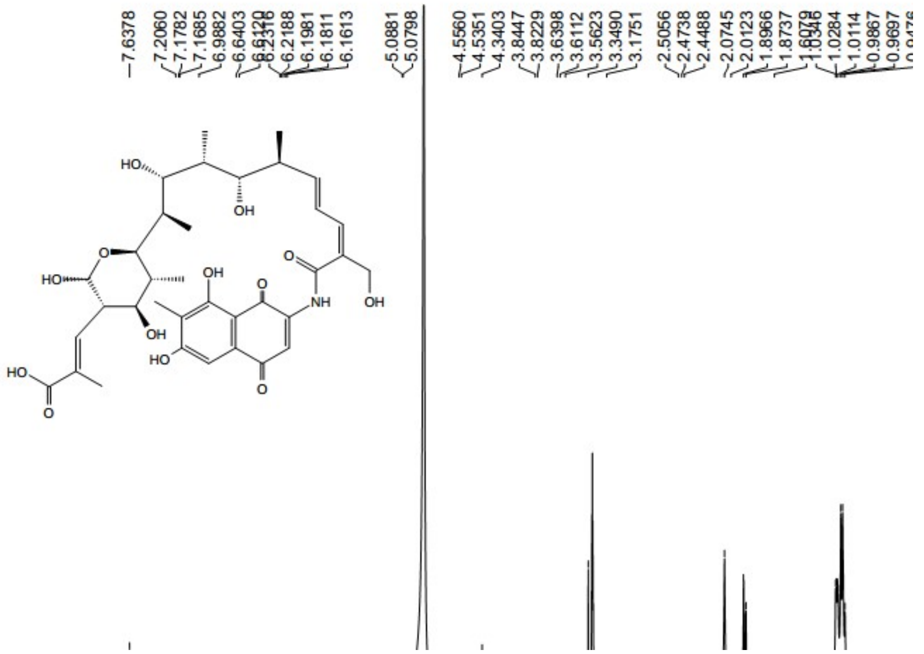

Figure S15. ^{1}H NMR spectrum of **2** in CD_3OD.

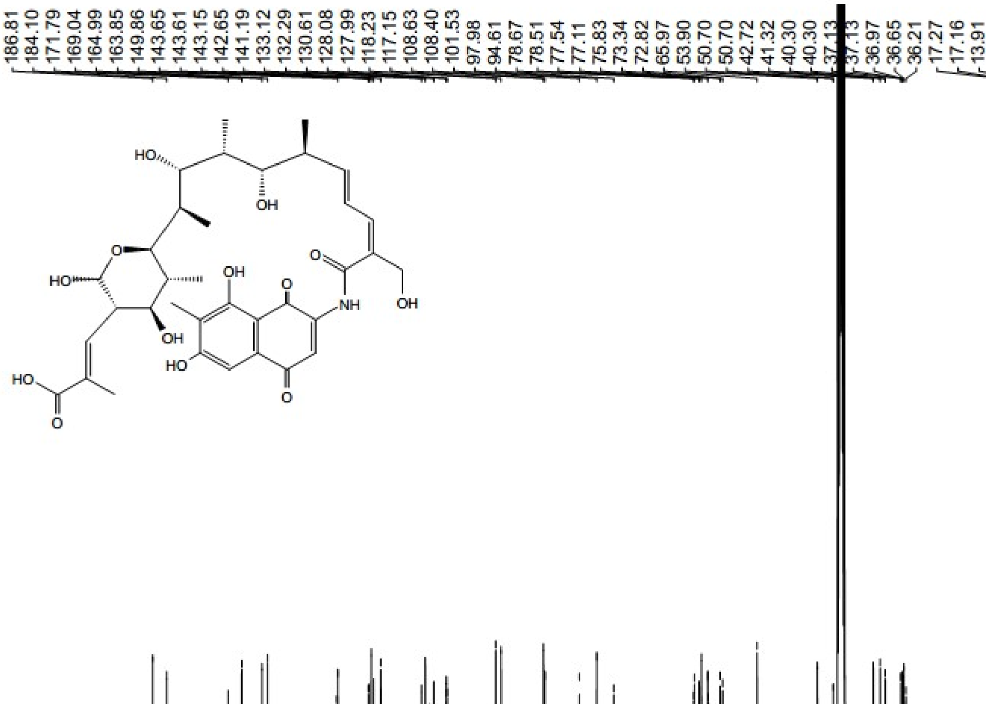

Figure S16. ^{13}C NMR spectrum of **2** in CD_3OD.

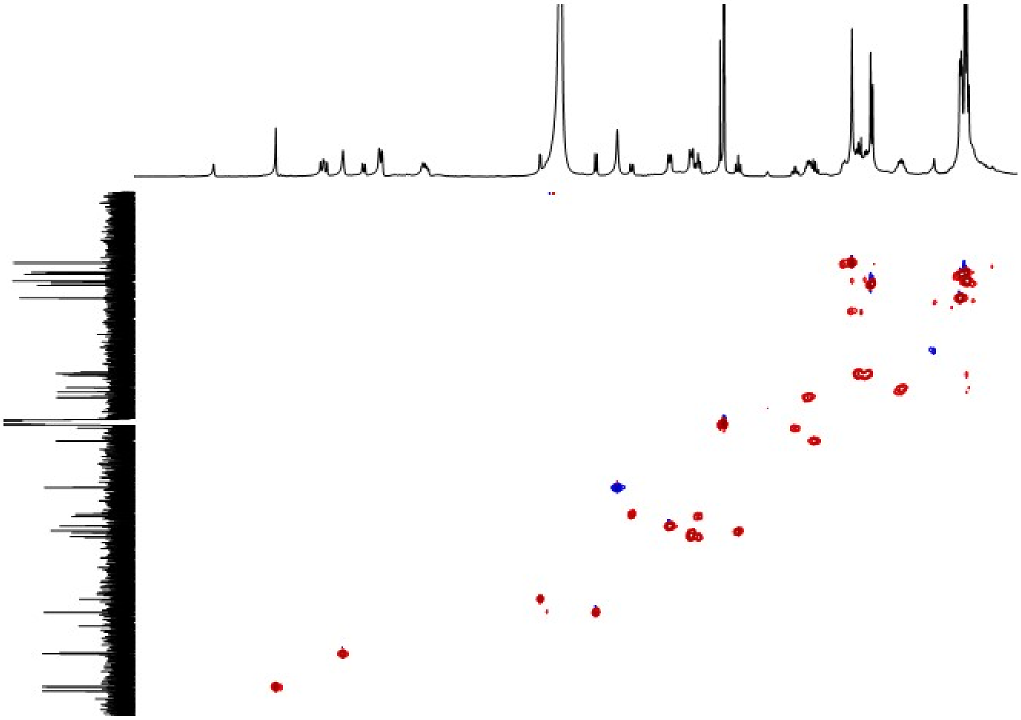

Figure S17. HSQC spectrum of **2** in CD_3OD.

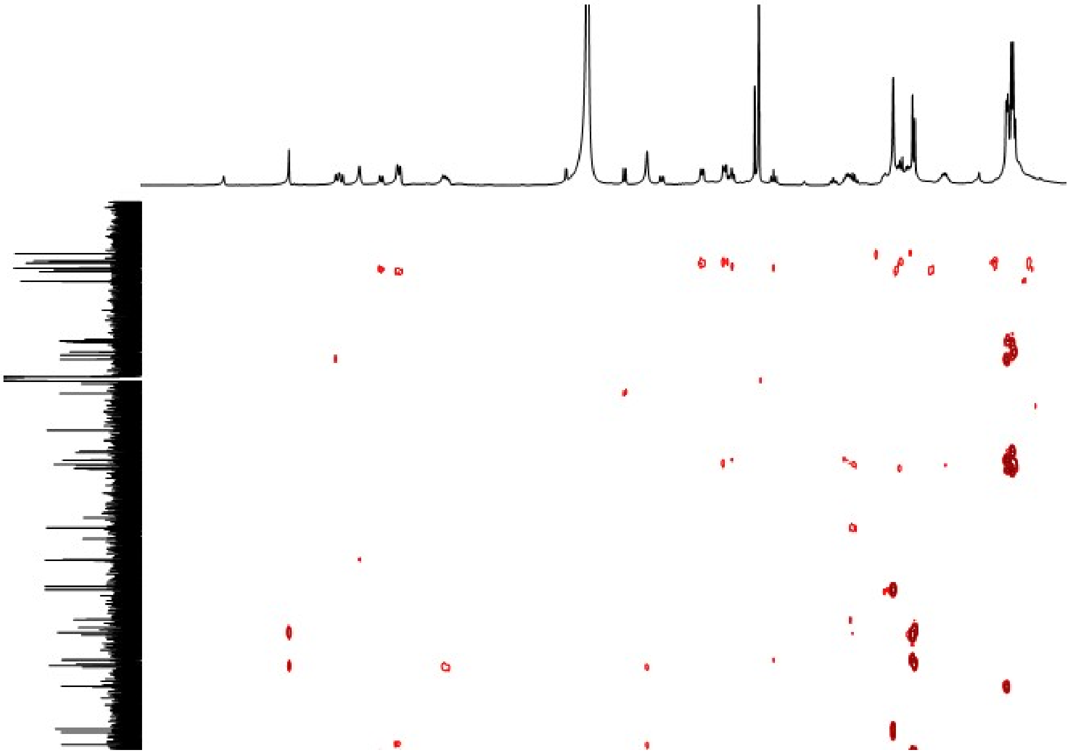

Figure S18. HMBC spectrum of **2** in CD_3OD.

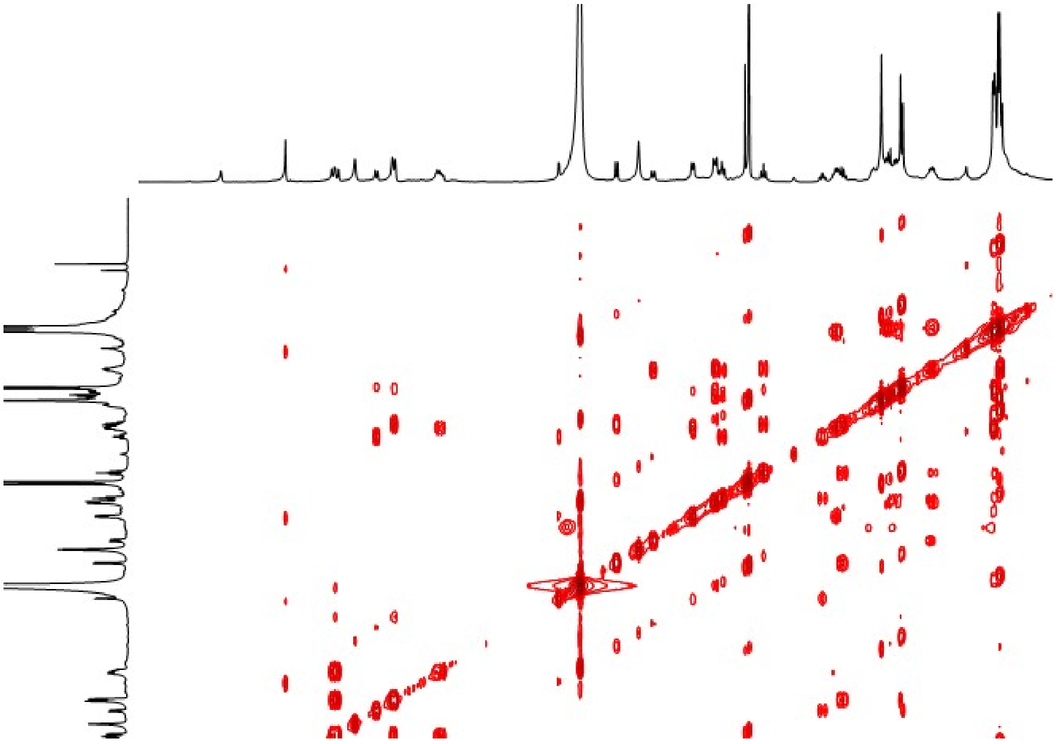

Figure S19. ^{1}H-^{1}H COSY spectrum of **2** in CD_3OD.

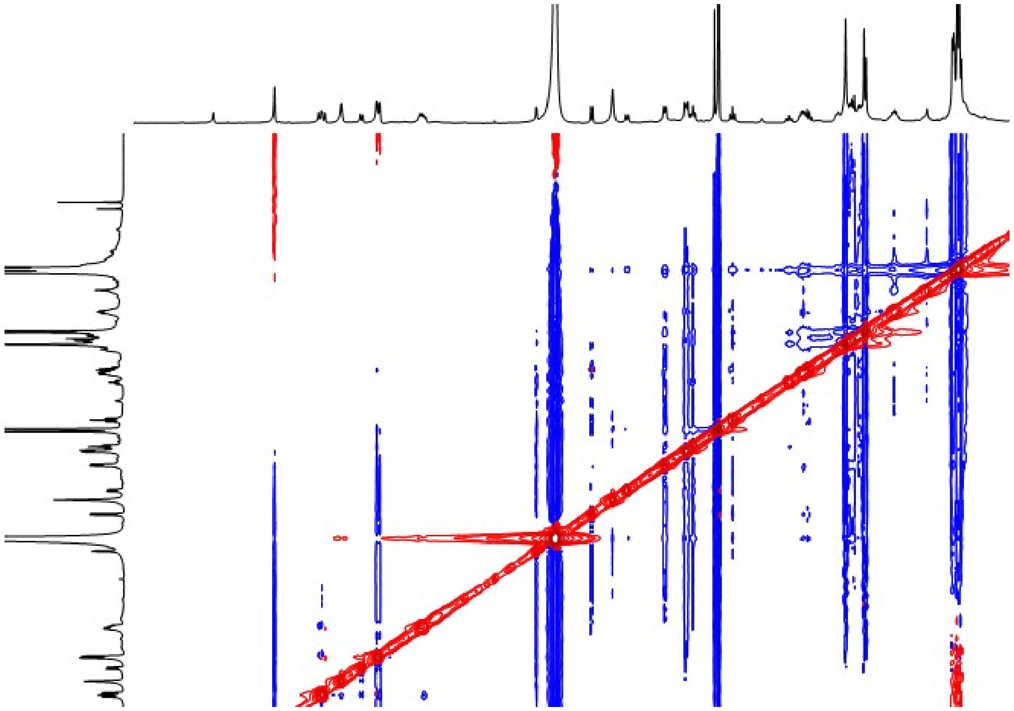

Figure S20. NOESY spectrum of **2** in CD_3OD.

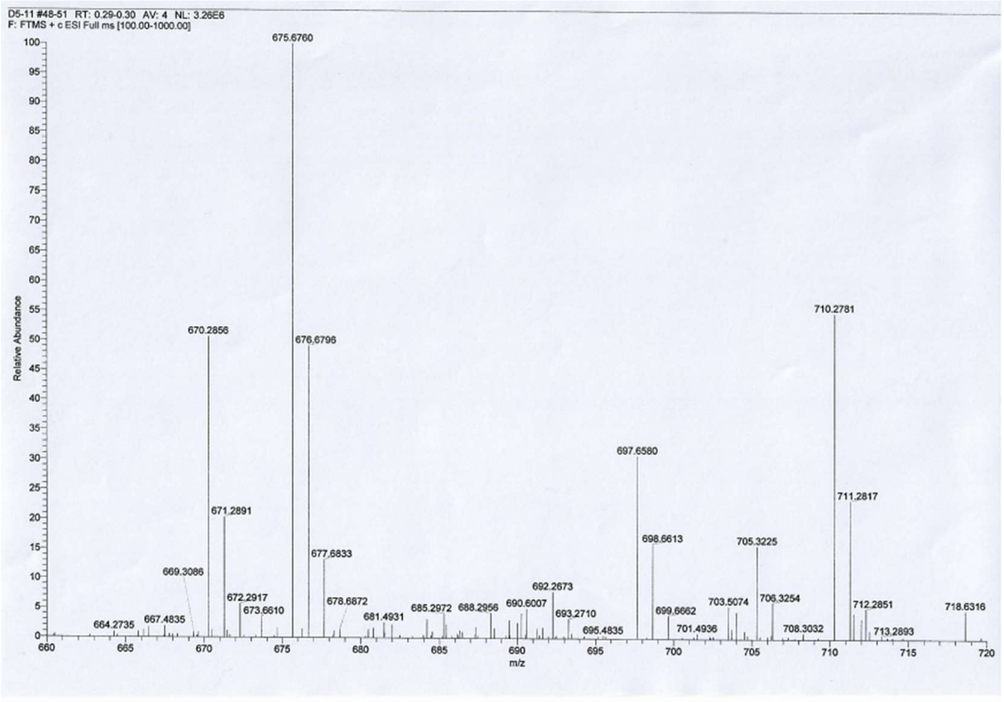

Figure S21. HRESIMS spectrum of **2**.

Figures S22–28. NMR and HRESIMS spectra of **3**.

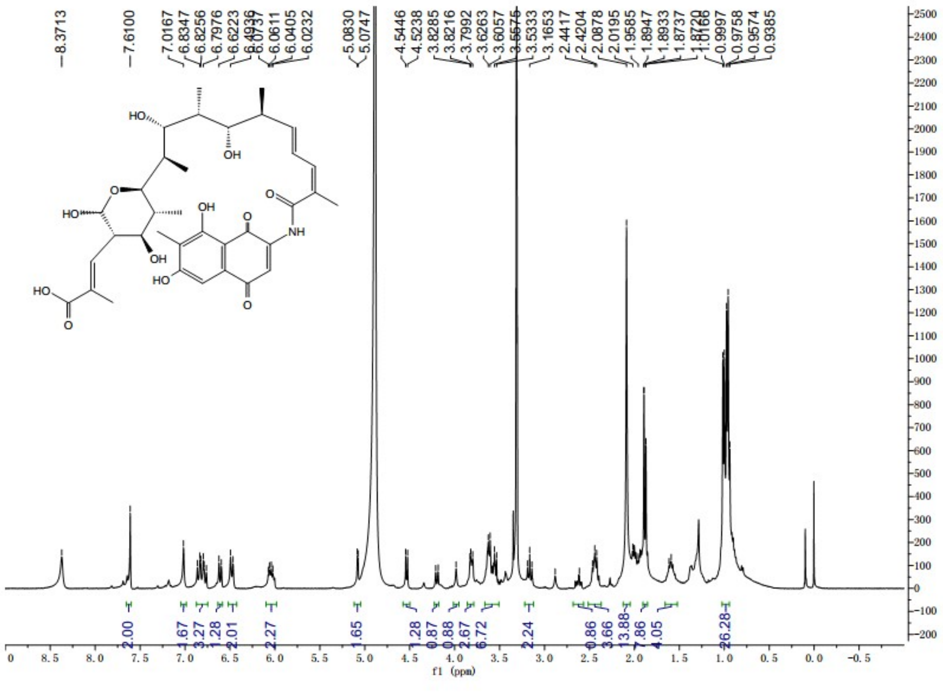

Figure S22. ^{1}H NMR spectrum of **3** in CD_3OD.

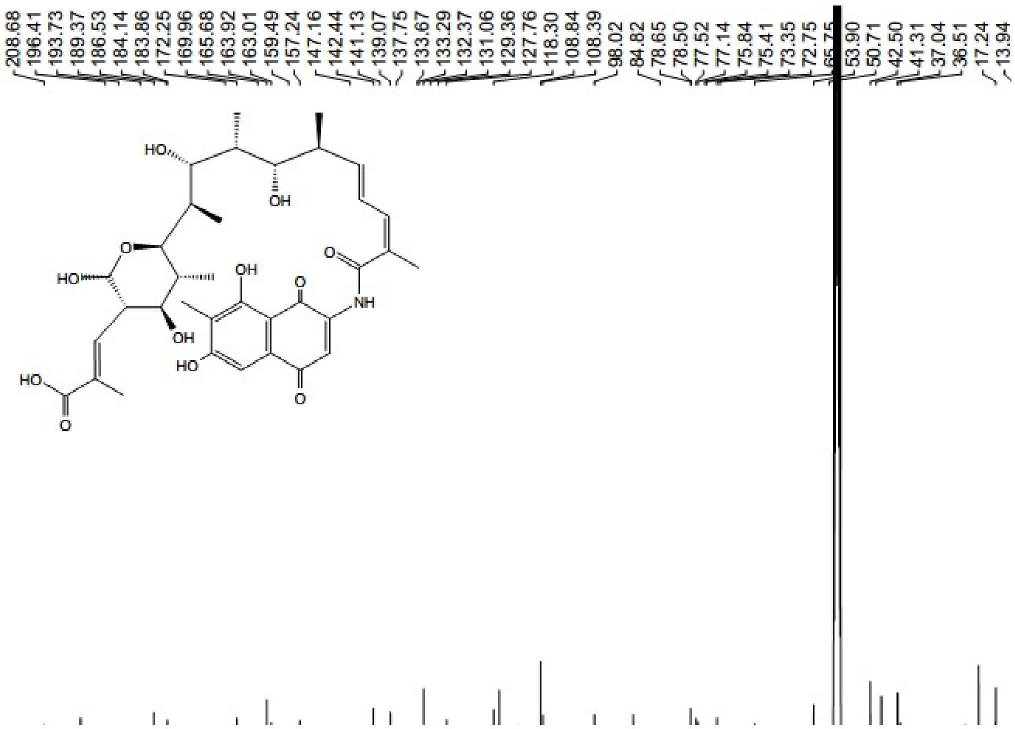

Figure S23. ^{13}C NMR spectrum of **3** in CD_3OD.

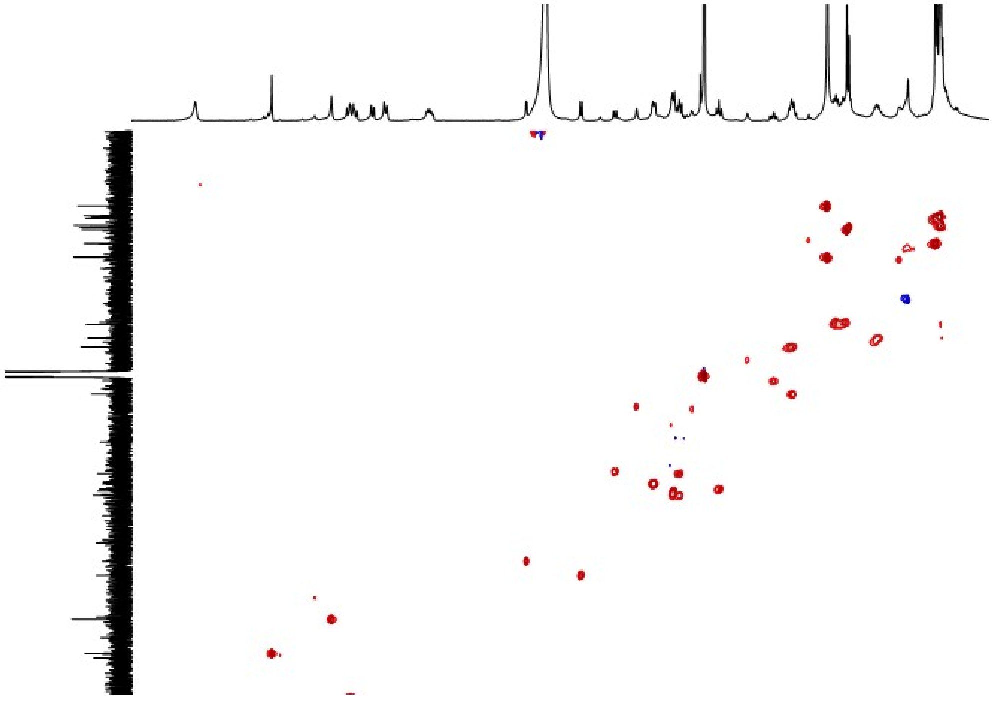

Figure S24. HSQC spectrum of **3** in CD_3OD.

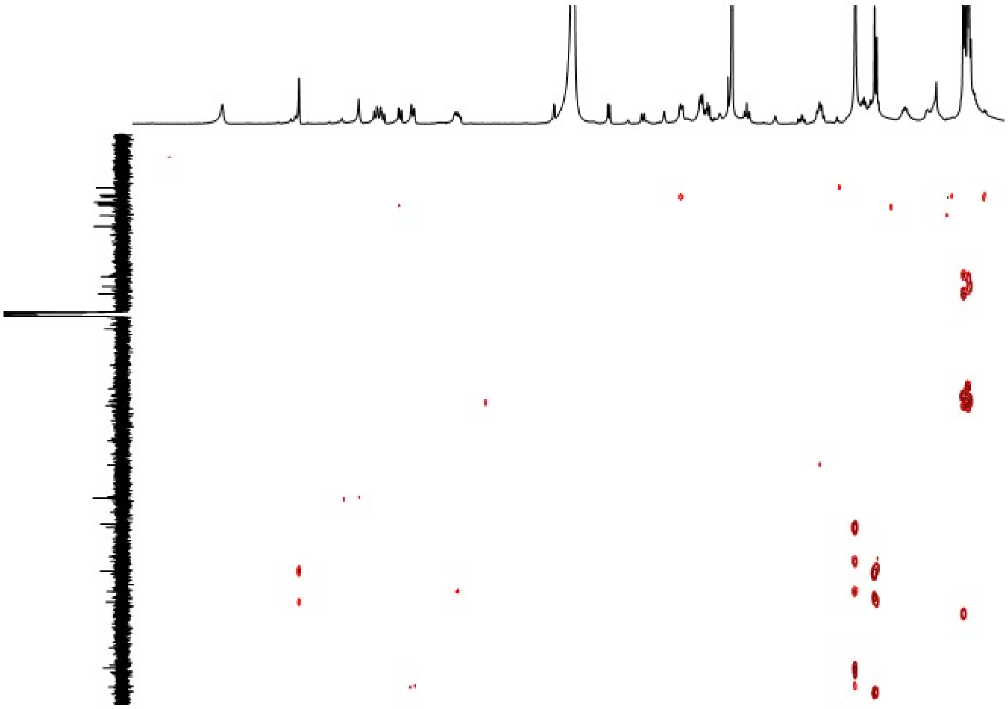

Figure S25. HMBC spectrum of **3** in CD_3OD.

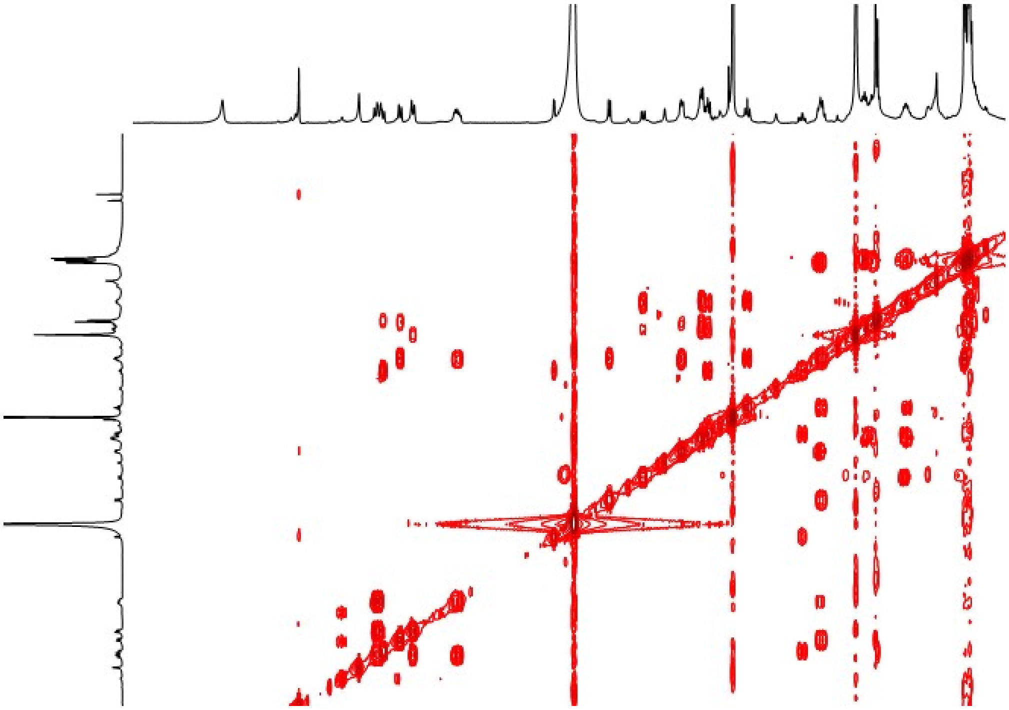

Figure S26. ^1H-^1H COSY spectrum of **3** in CD_3OD.

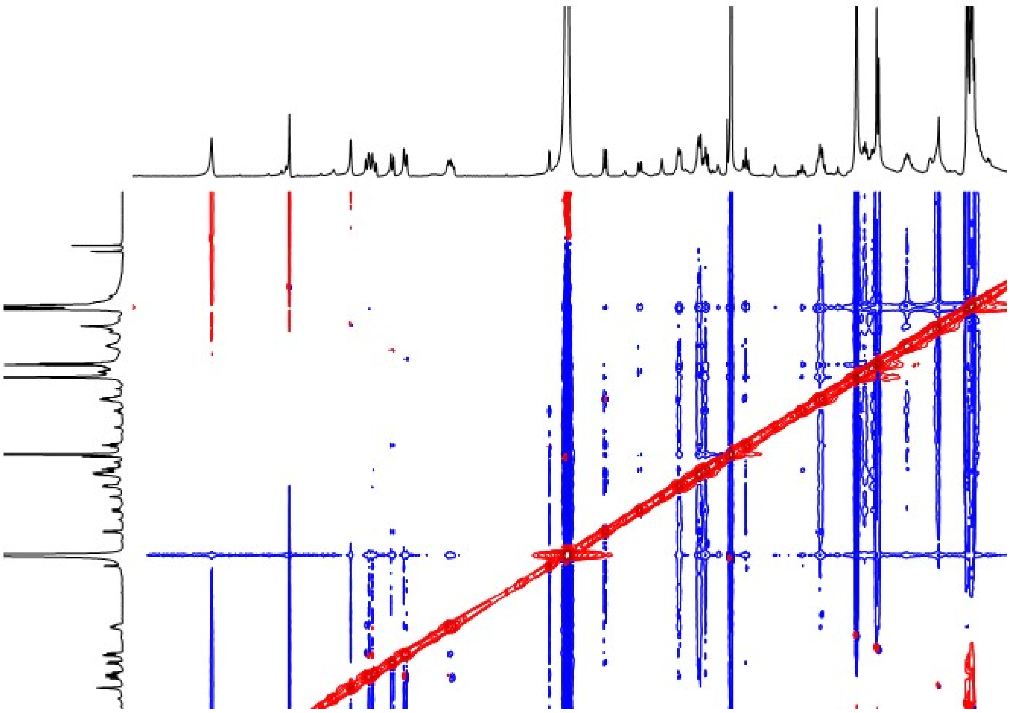

Figure S27. NOESY spectrum of **3** in CD_3OD.

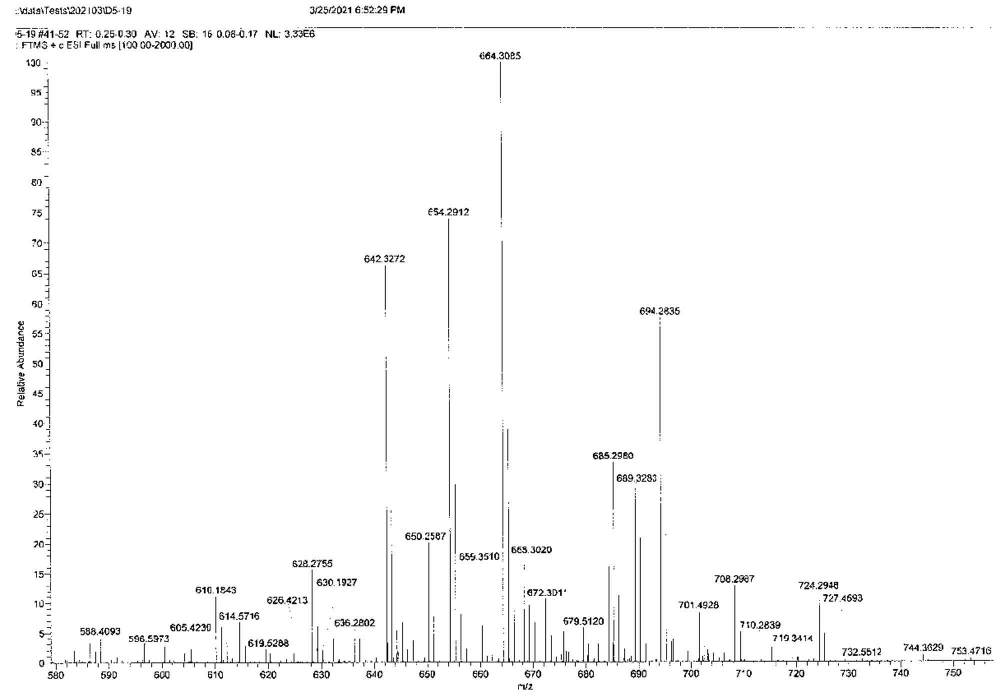

Figure S28. HRESIMS spectrum of **3**.

Figures S29–34. NMR and HRESIMS spectra of **4**.

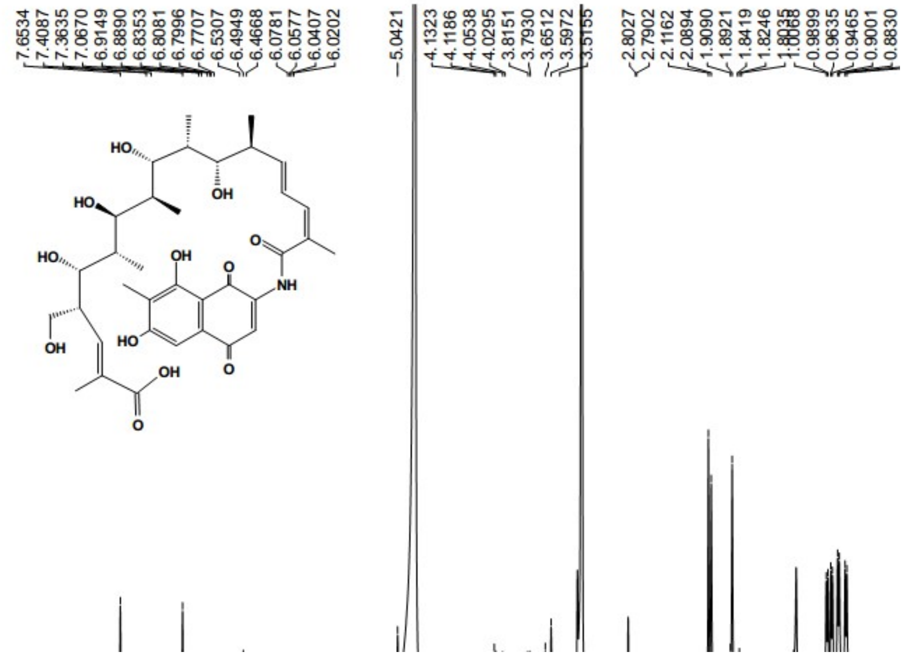

Figure S29. ^{1}H NMR spectrum of **4** in CD_3OD.

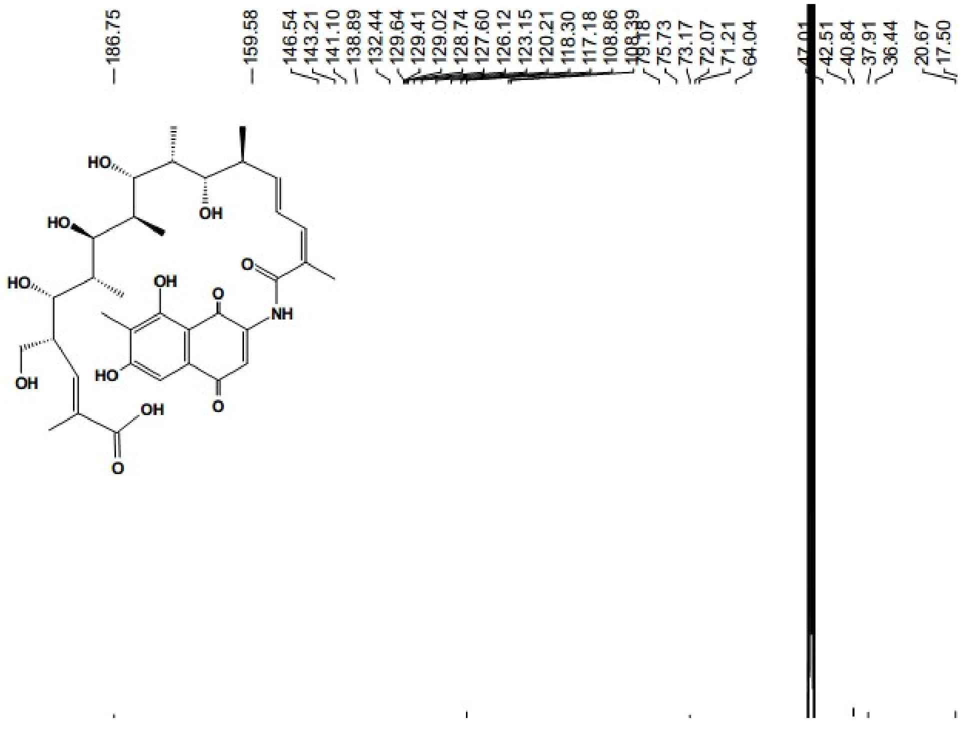

Figure S30. ^{13}C NMR spectrum of **4** in CD_3OD.

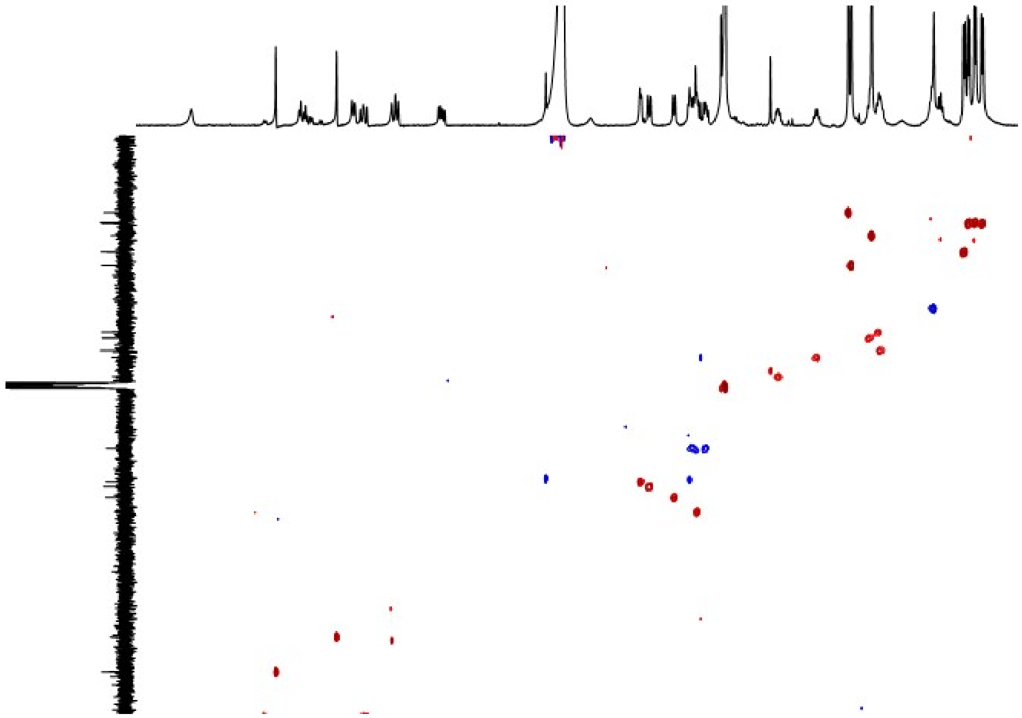

Figure S31. HSQC spectrum of **4** in CD_3OD.

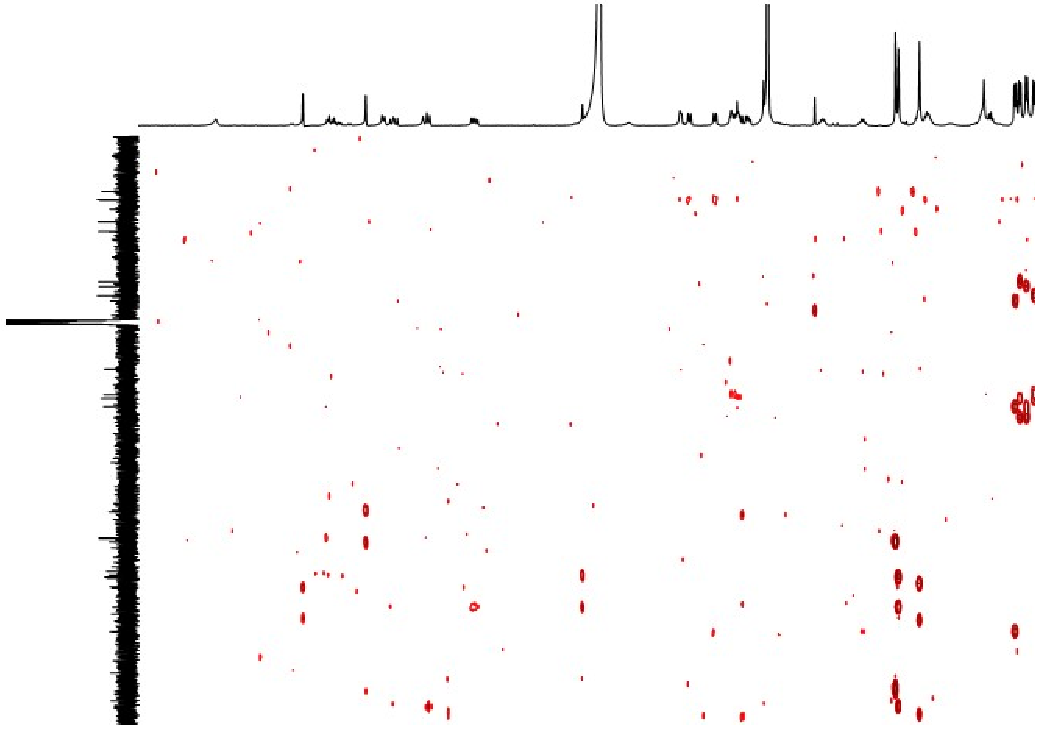

Figure S32. HMBC spectrum of **4** in CD_3OD.

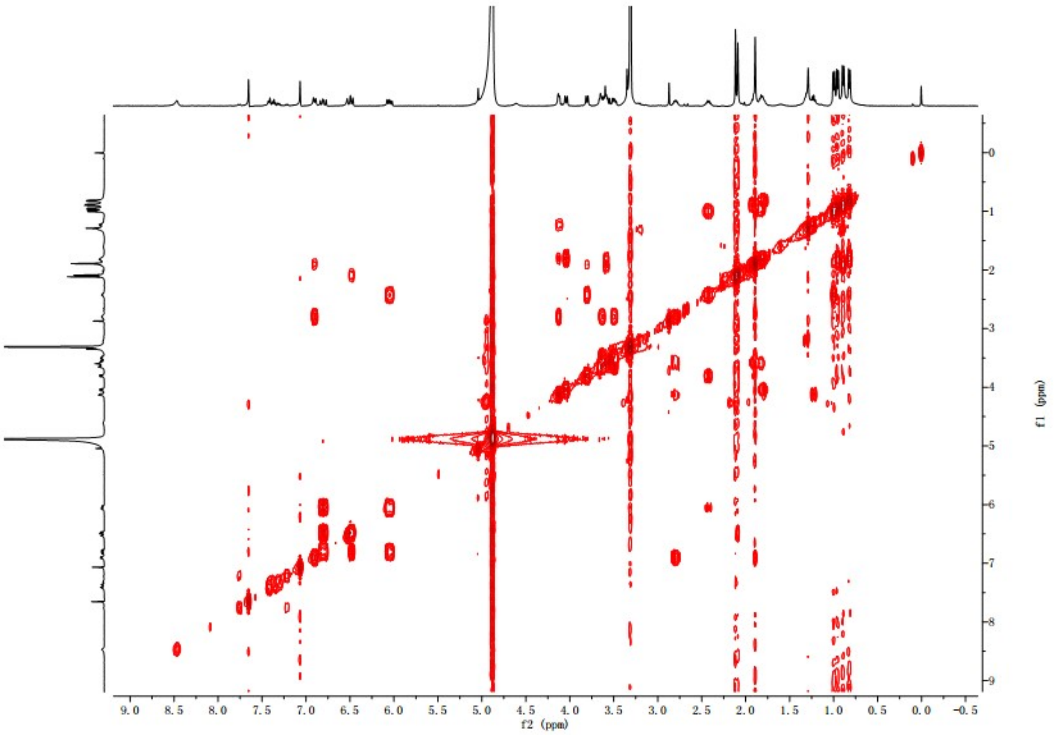

Figure S33. ^1H-^1H COSY spectrum of **4** in CD_3OD.

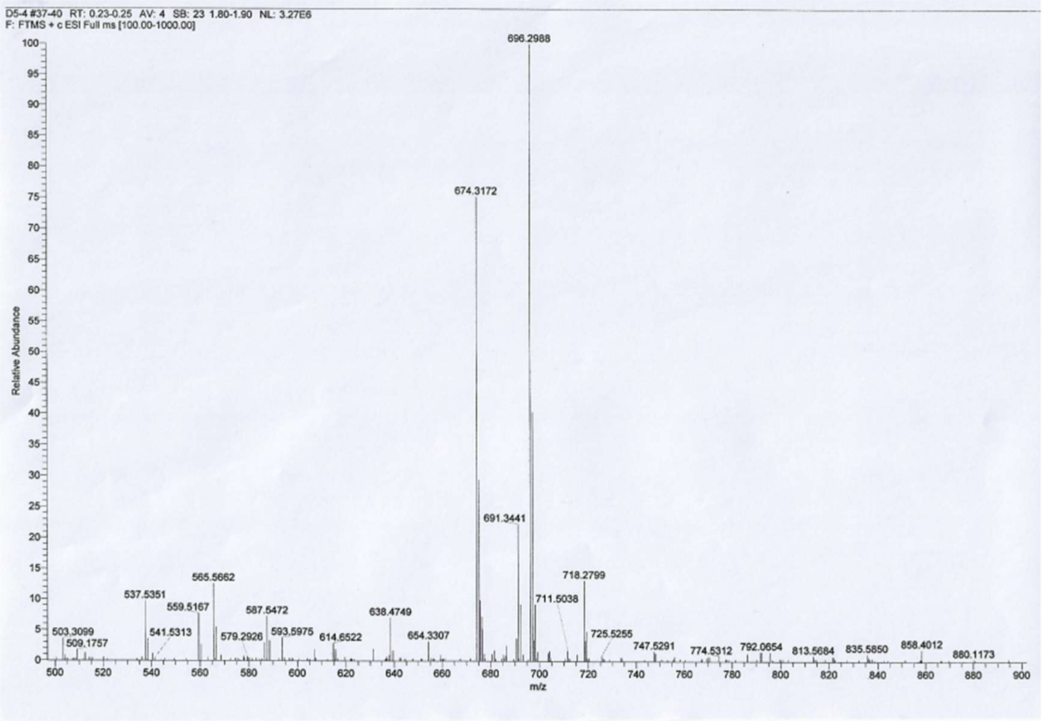

Figure S34. HRESIMS spectrum of **4**.

Figures S35–41. NMR and HRESIMS spectra of **5**.

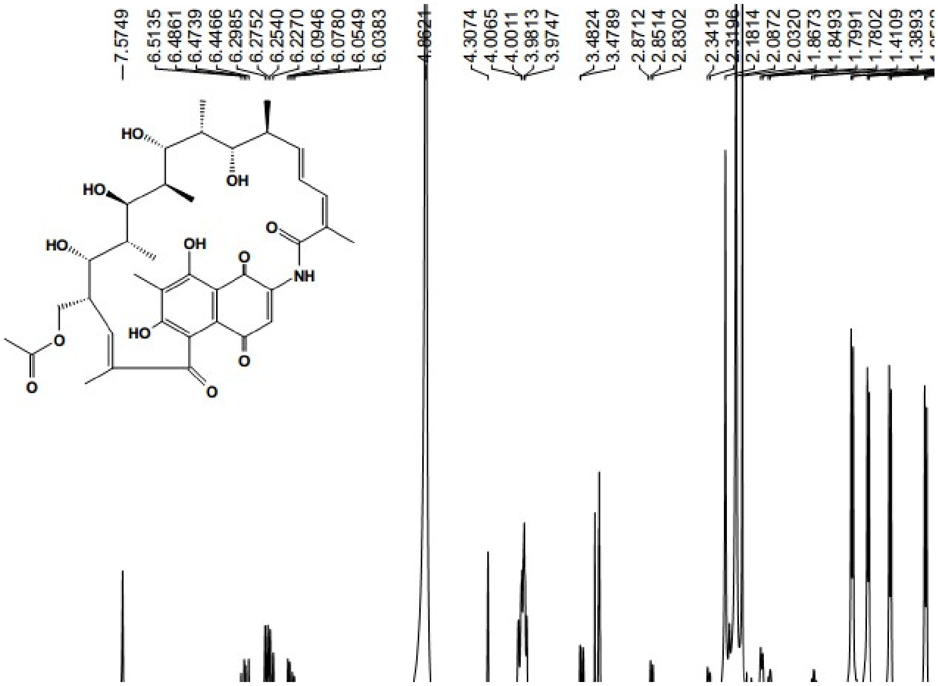

Figure S35. ^{1}H NMR spectrum of **5** in CD_3OD.

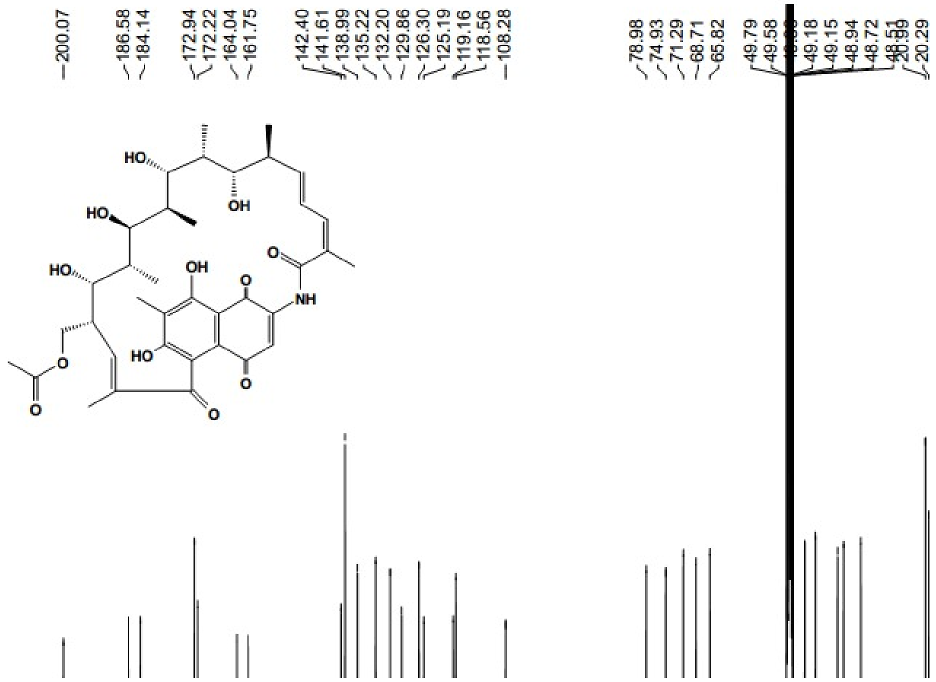

Figure S36. ^{13}C NMR spectrum of **5** in CD_3OD.

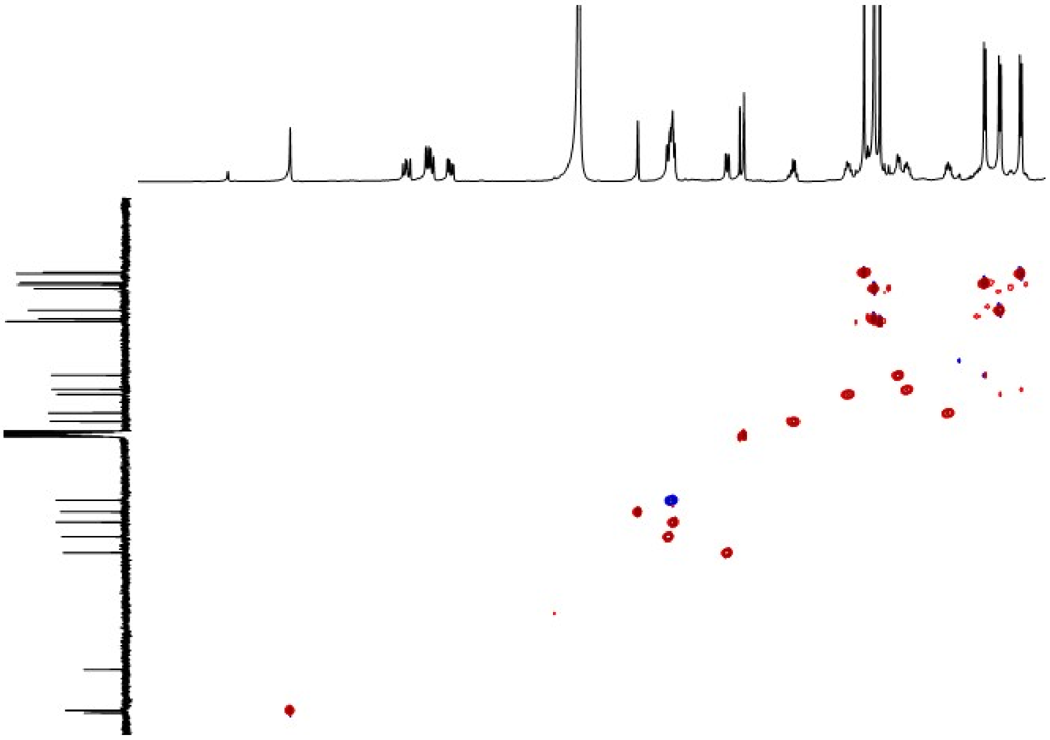

Figure S37. HSQC spectrum of **5** in CD_3OD.

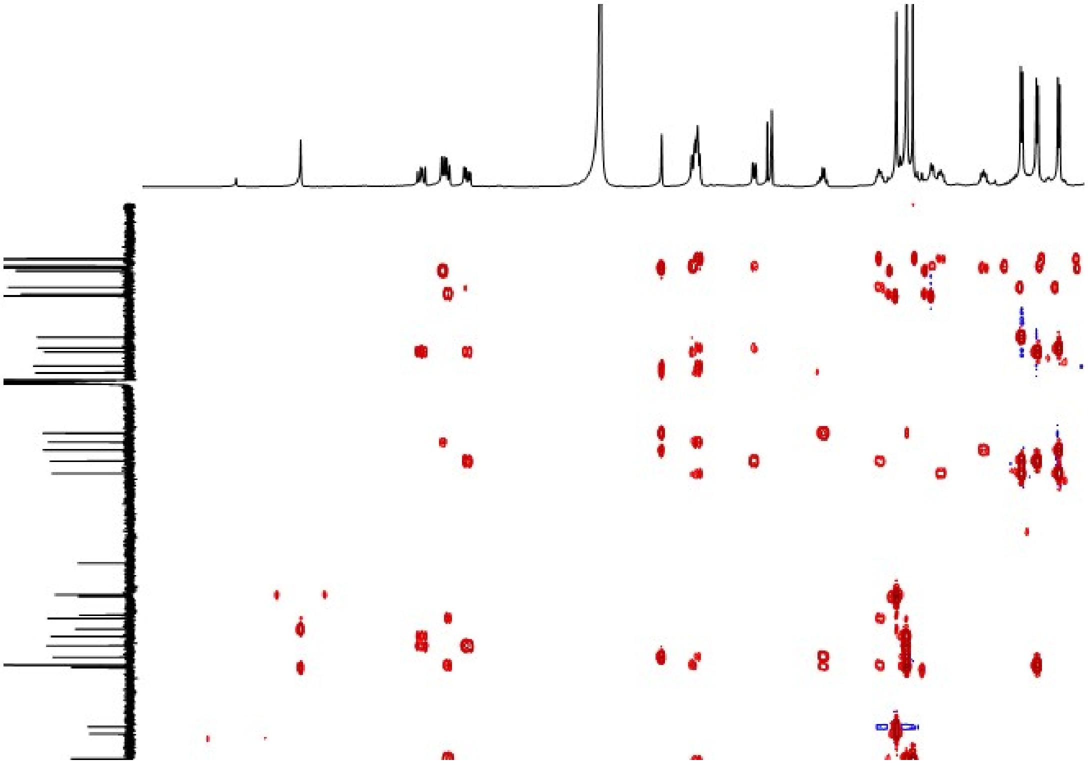

Figure S38. HMBC spectrum of **5** in CD_3OD.

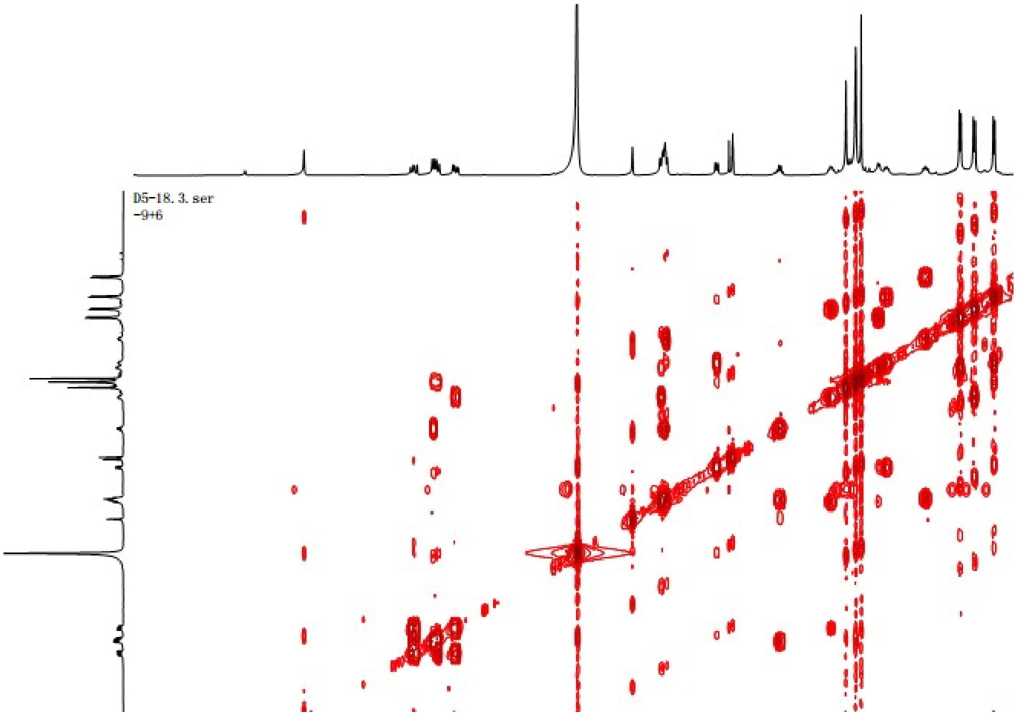

Figure S39. ^{1}H-^{1}H COSY spectrum of **5** in CD_3OD.

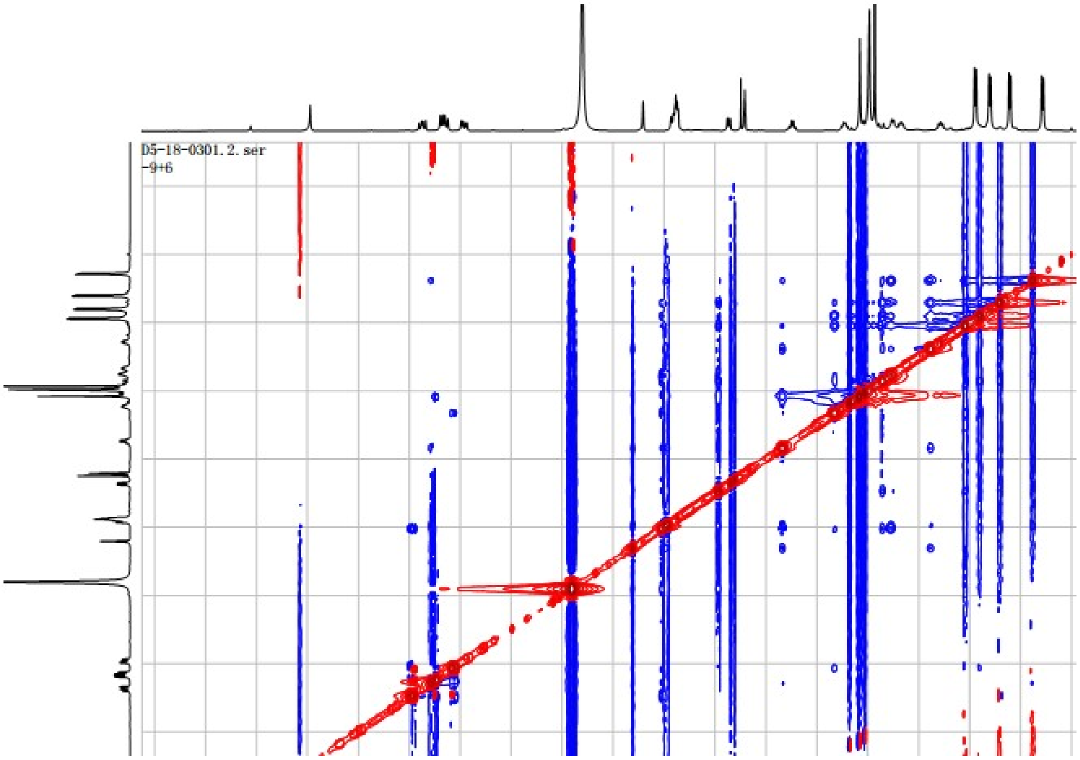

Figure S40. NOESY spectrum of **5** in CD_3OD.

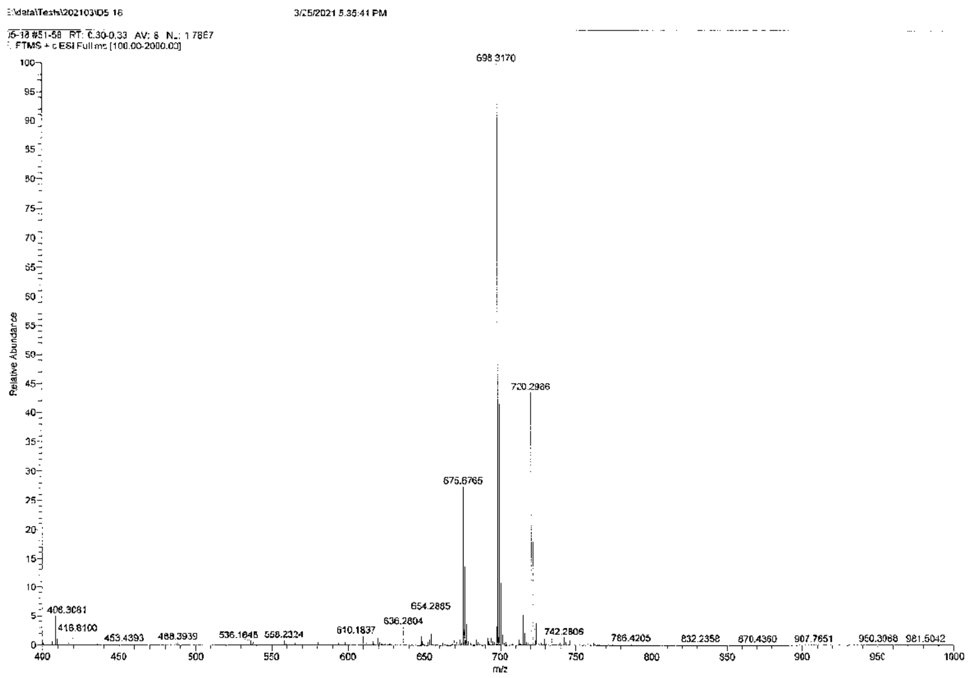

Figure S41. HRESIMS spectrum of **5**.

Figures S42–48. NMR and HRESIMS spectra of **6**.

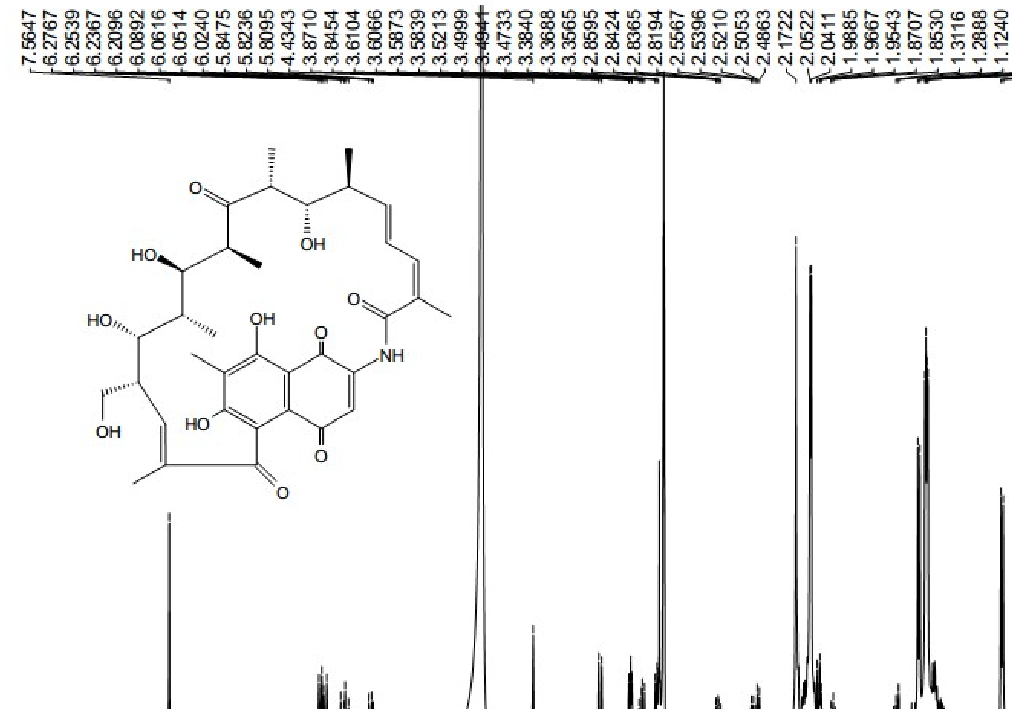

Figure S42. ^1H NMR spectrum of **6** in CD_3OD.

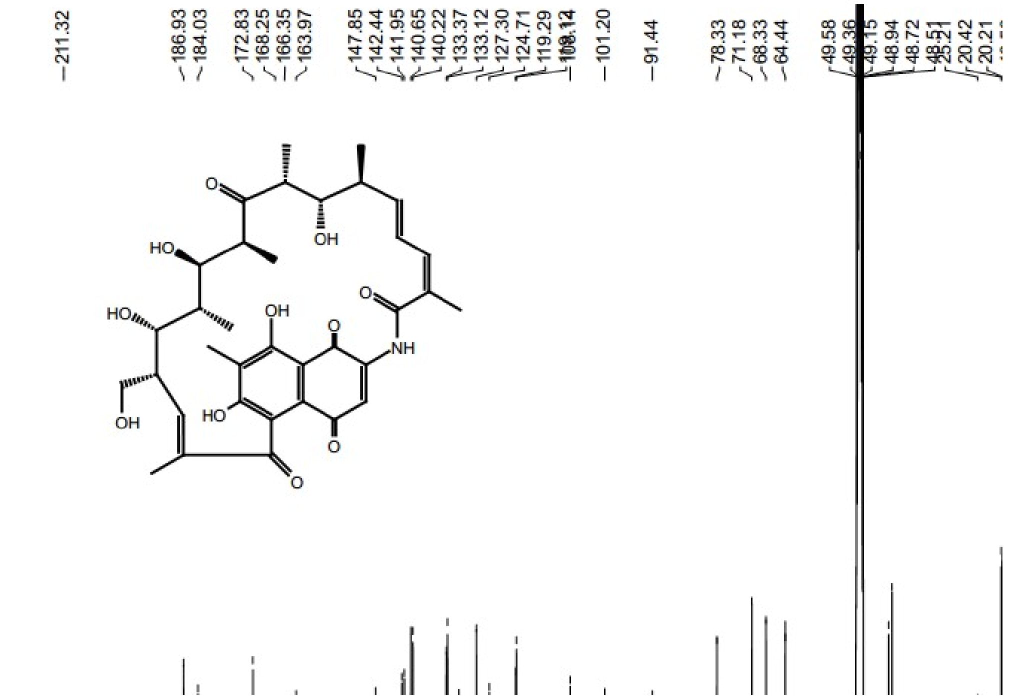

Figure S43. ^{13}C NMR spectrum of **6** in CD_3OD.

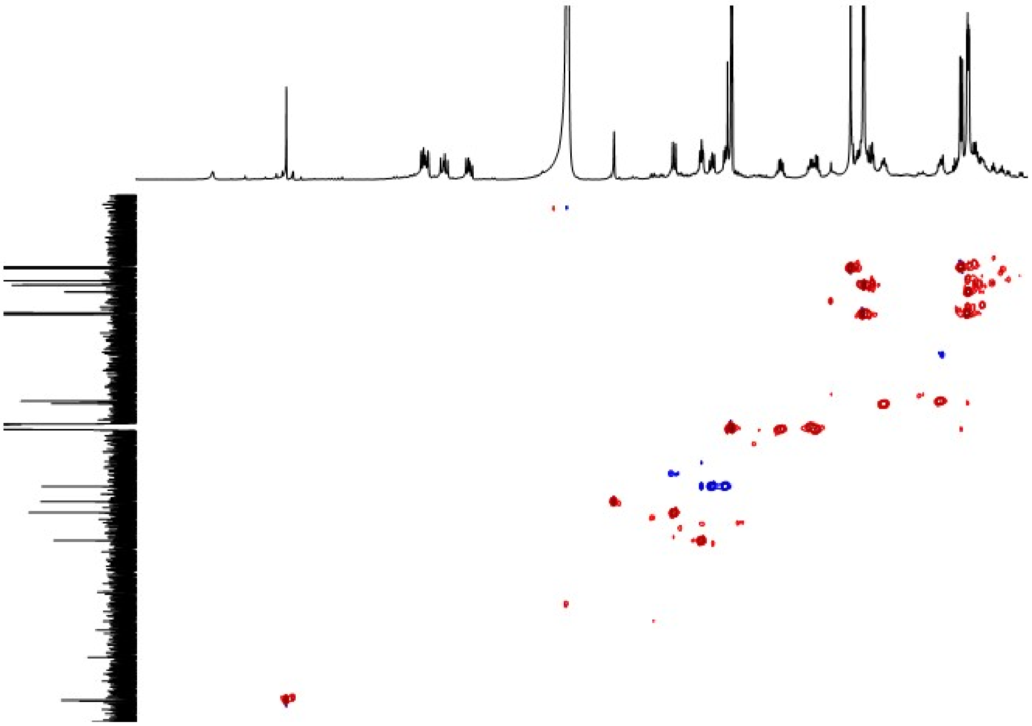

Figure S44. HSQC spectrum of **6** in CD_3OD.

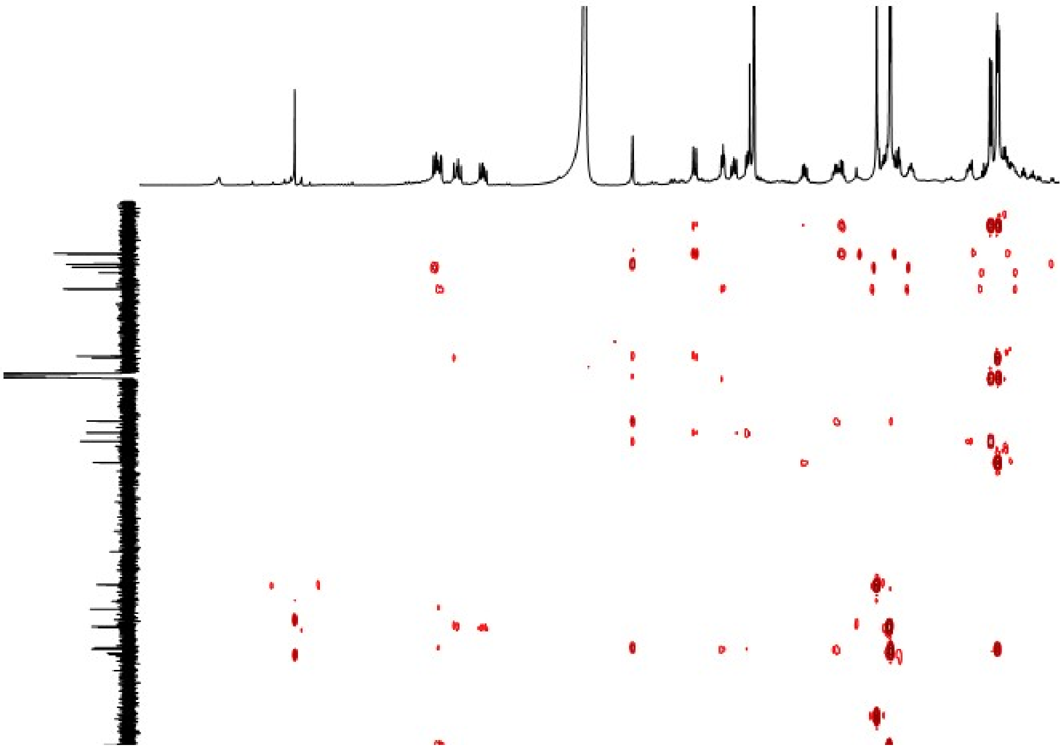

Figure S45. HMBC spectrum of **6** in CD_3OD.

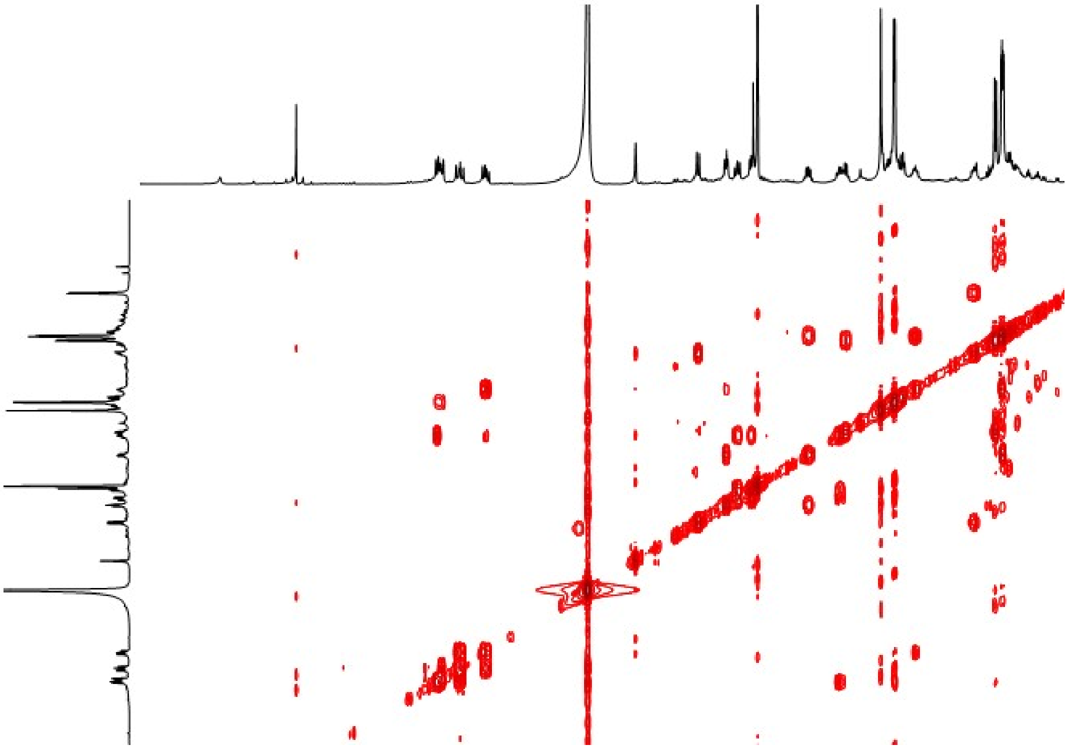

Figure S46. ^1H-^1H COSY spectrum of **6** in CD_3OD.

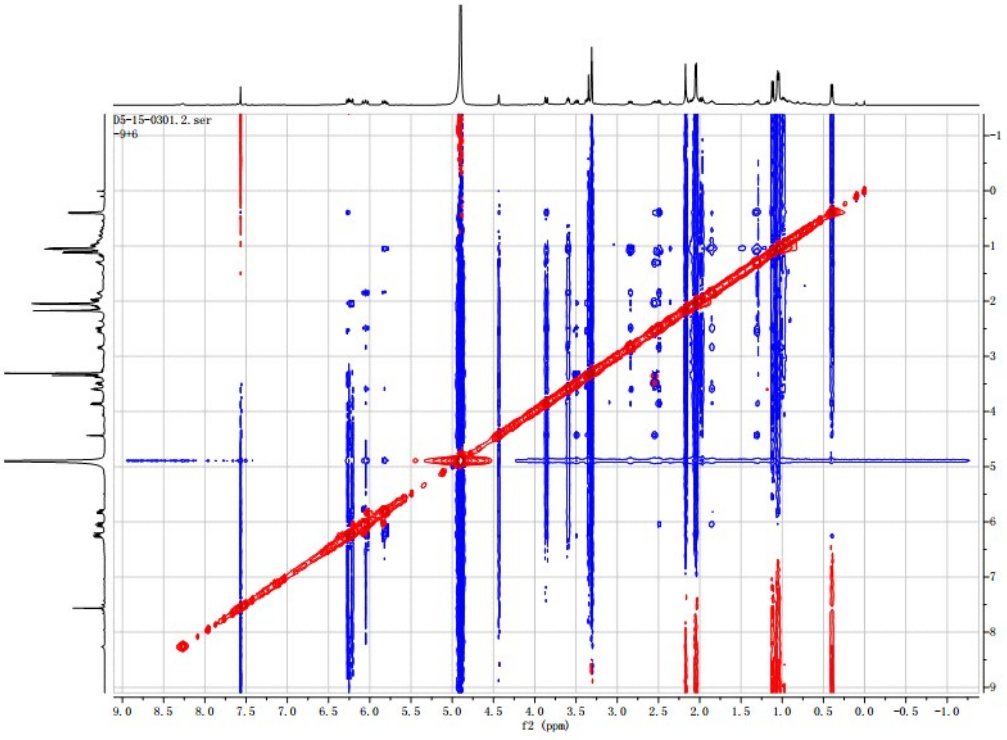

Figure S47. NOESY spectrum of **6** in CD_3OD.

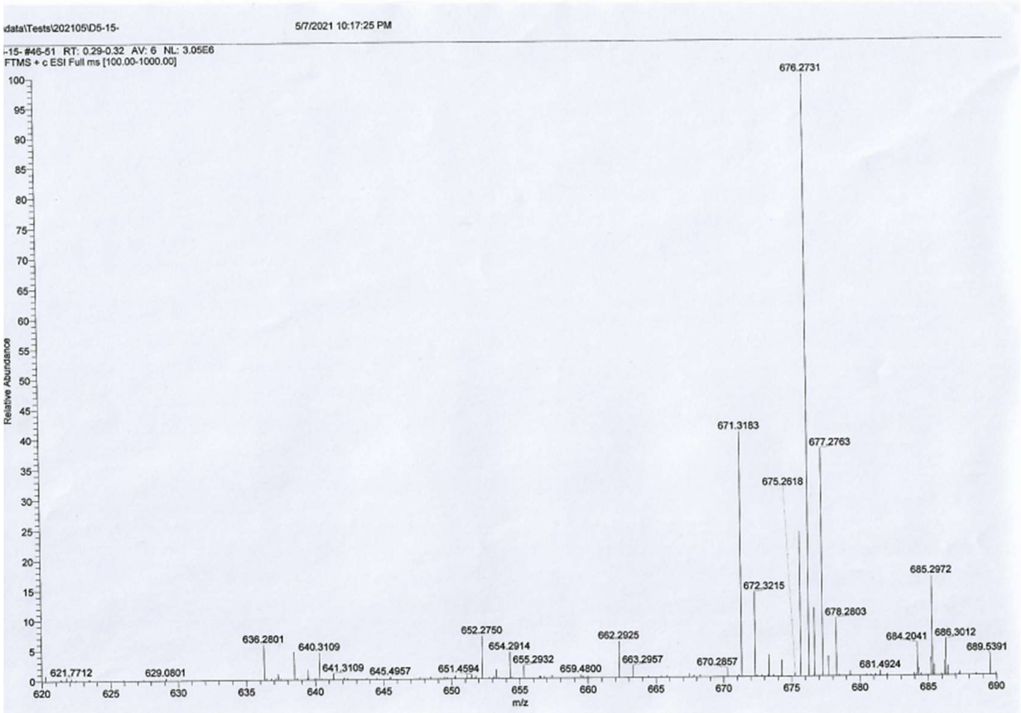

Figure S48. HRESIMS spectrum of **6**.

Figures S49–55. NMR and HRESIMS spectra of **7**.

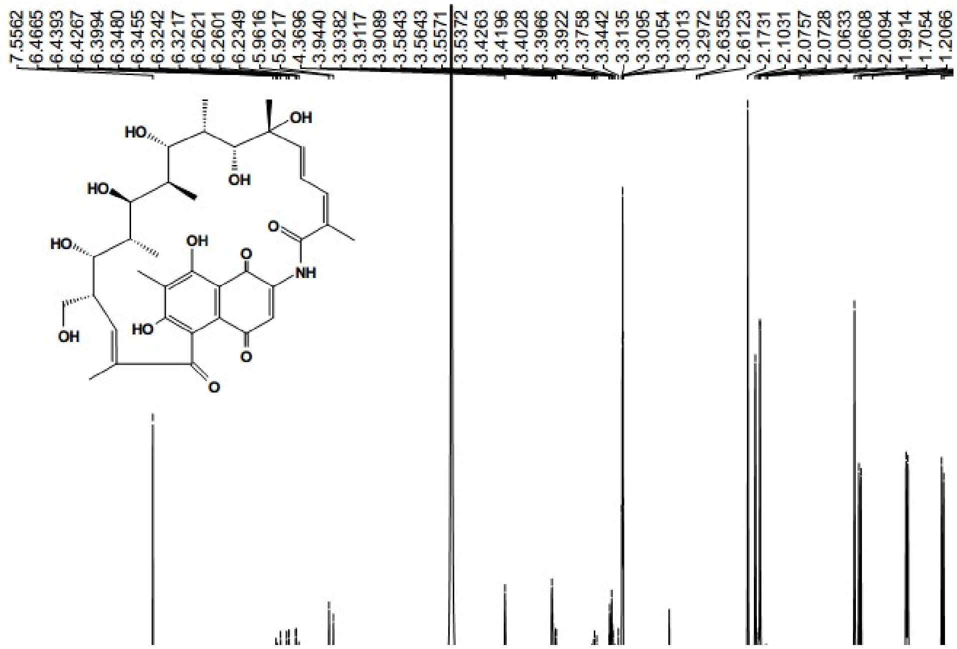

Figure S49. ^{1}H NMR spectrum of **7** in CD_3OD.

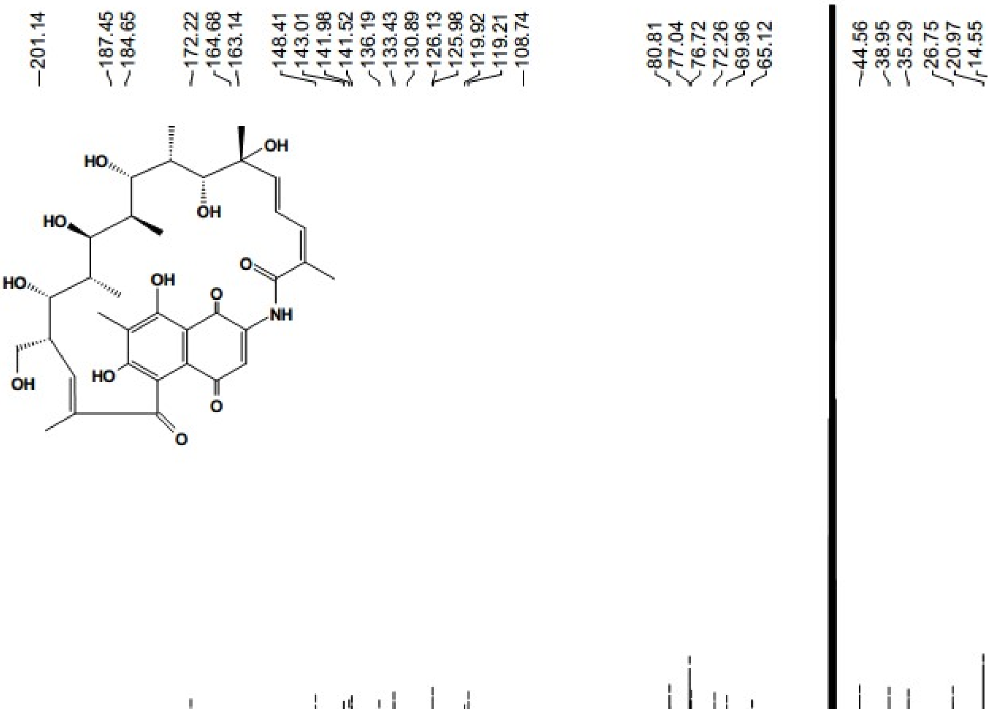

Figure S50. ^{13}C NMR spectrum of **7** in CD_3OD.

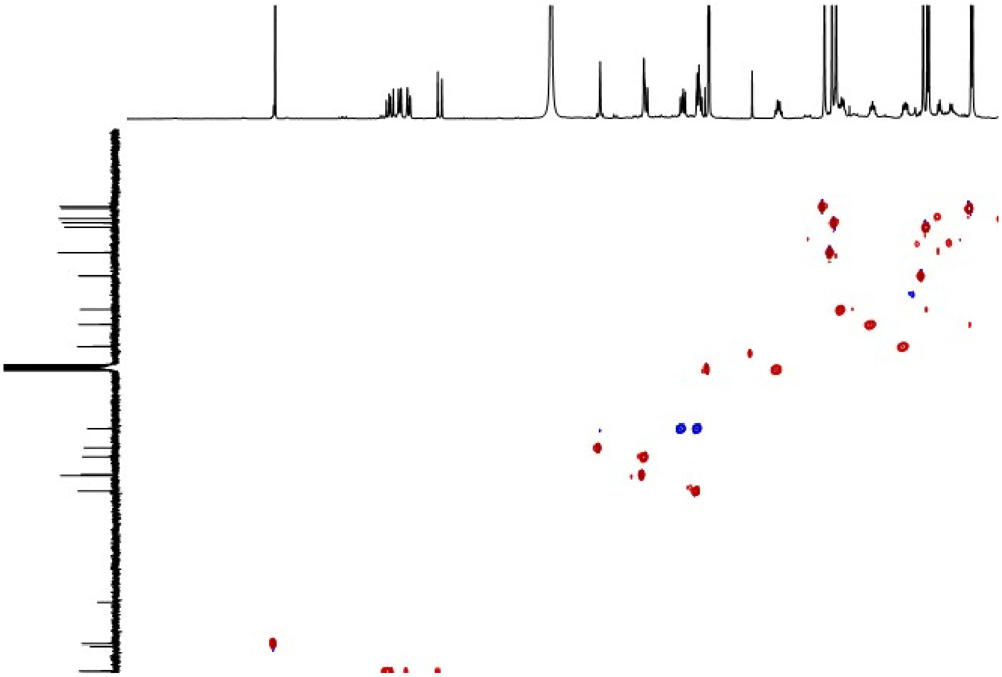

Figure S51. HSQC spectrum of **7** in CD_3OD.

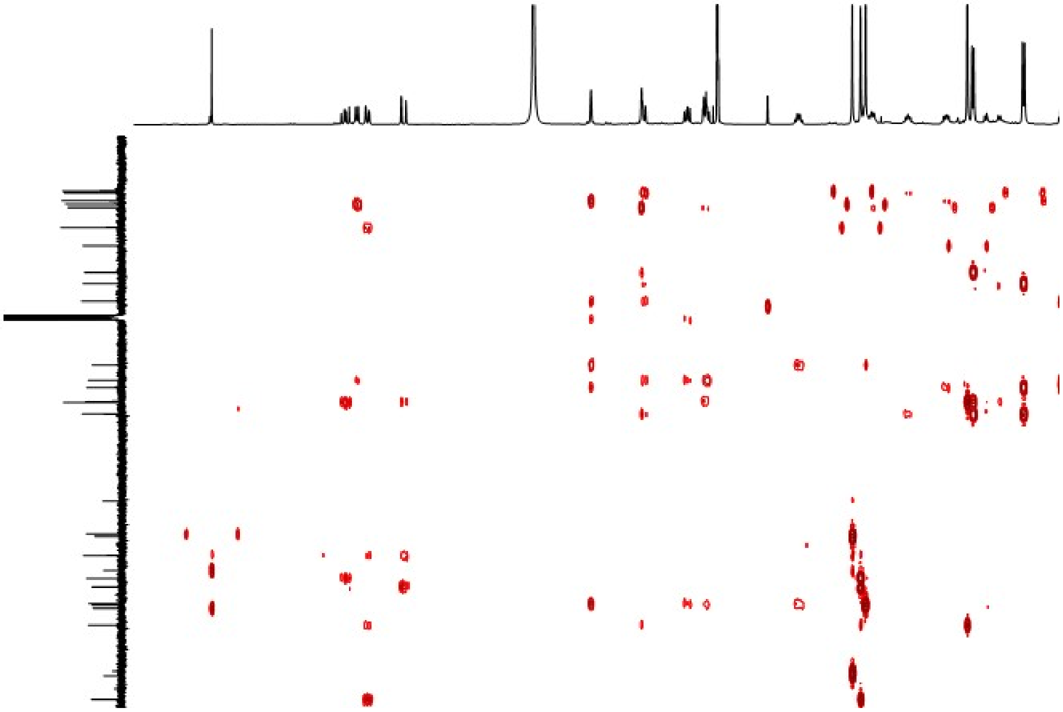

Figure S52. HMBC spectrum of **7** in CD_3OD.

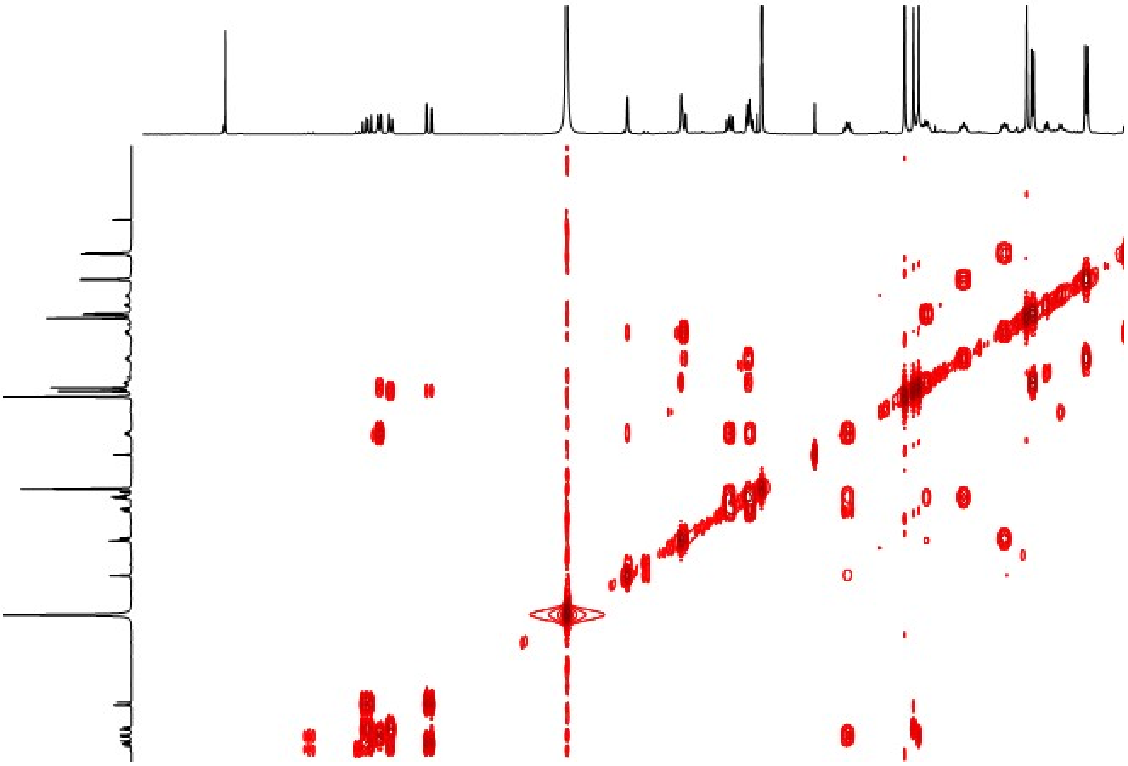

Figure S53. ^{1}H-^{1}H COSY spectrum of **7** in CD_3OD.

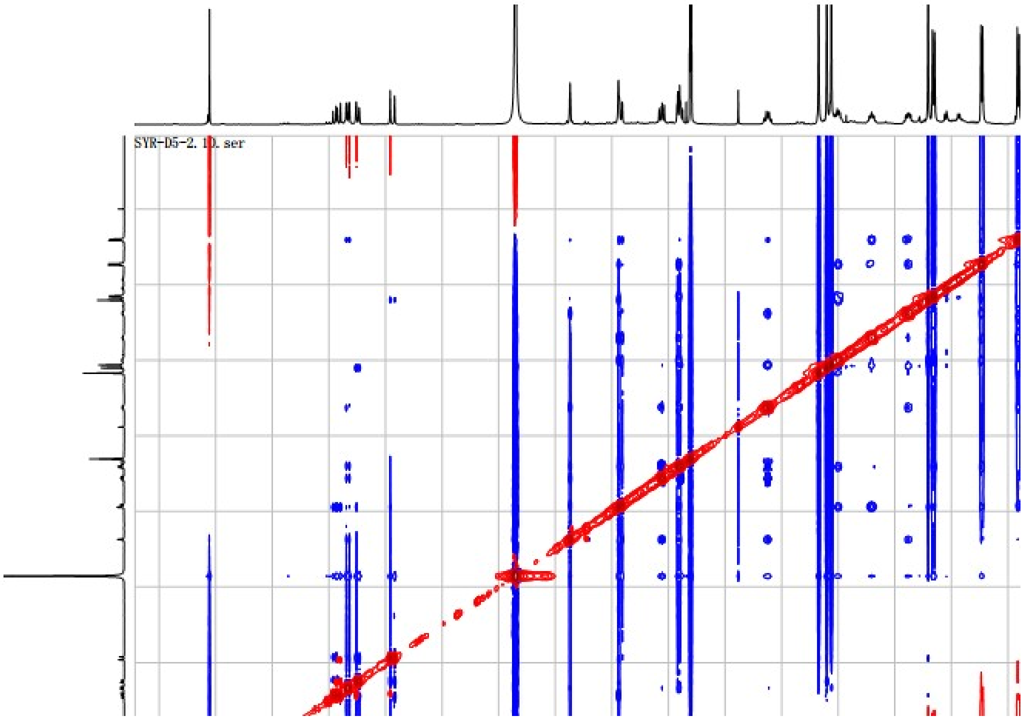

Figure S54. NOESY spectrum of **7** in CD_3OD.

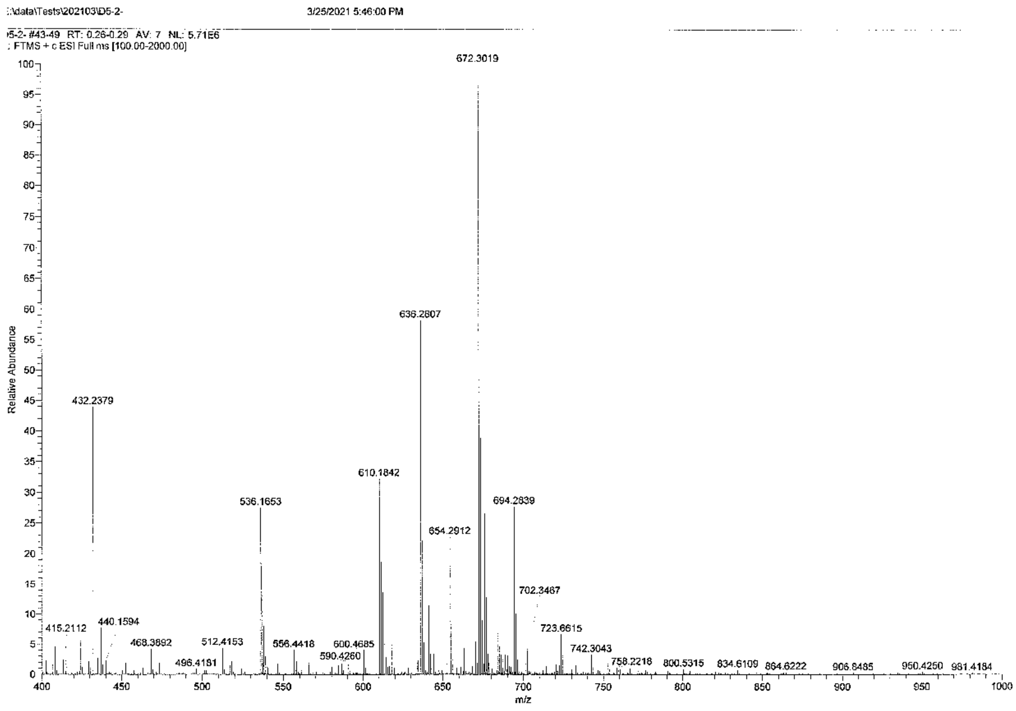

Figure S55. HRESIMS spectrum of **7**.

4 Bioactivity

4.1. Antimicrobial activity of compounds **1–13** (Figure S56)

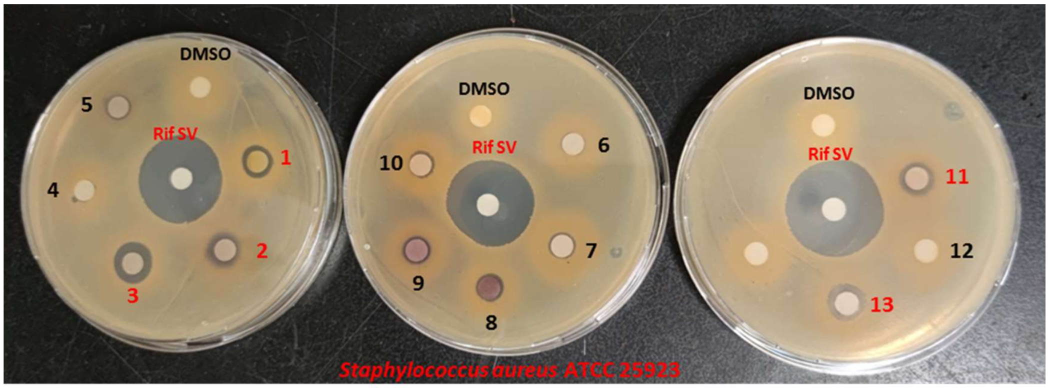

Figure S56. Antimicrobial activity of compounds **1–13.** The inhibitory activity of compounds **1–13** (40 μg each) against *Staphylococcus aureus* ATCC 25923. In the assays against bacteria, rifamycin SV sodium salt hexahydrate was used as the positive control. Dimethyl sulfoxide (DMSO) was used as the blank control

for antimicrobial experiments.

Table S14. Diameters of the inhibition zones and MICs of compounds **1–3** against *Staphylococcus aureus* ATCC 25923.

Test compounds	**1**	**2**	**3**	Rifamycin SV
Diameter of the inhibition zones (mm)	8	6	13	32
MIC (μg/mL)	5	40	0.5	0.05

4.2 Cytotoxicity assay

TableS15. Antiproliferative activity against HeLa and Caco-2 cells of compounds **1–13**.

	Cell viability			
	HeLa		Caco-2	
Compounds/μM	50	10	50	10
Blank	100.8569	101.2608	102.1205	99.27931
DMSO	97.85329	103.2322	98.89994	101.0048
VP-16	48.83457	74.92621	65.81704	90.33347
**1**	59.04932	71.38563	58.83704	86.15833
**2**	85.52472	94.18118	81.51816	95.27551
**3**	56.22951	87.60141	54.88866	89.36056
**4**	89.61155	98.84057	84.35121	97.59661
**5**	88.68462	99.74119	78.20783	94.16187
**6**	90.08352	99.40384	78.40975	94.21082

**7**	90.74119	96.74377	83.69445	93.83348
**8**	82.30292	96.93411	68.40941	89.34017
**9**	85.27713	93.88407	100.38479	93.6132
**10**	76.93016	91.7068	81.54116	92.20993
**11**	87.07372	96.46987	92.93604	96.18927
**12**	87.01028	94.85123	92.52812	96.37283
**13**	87.74377	95.25202	90.5823	93.85184

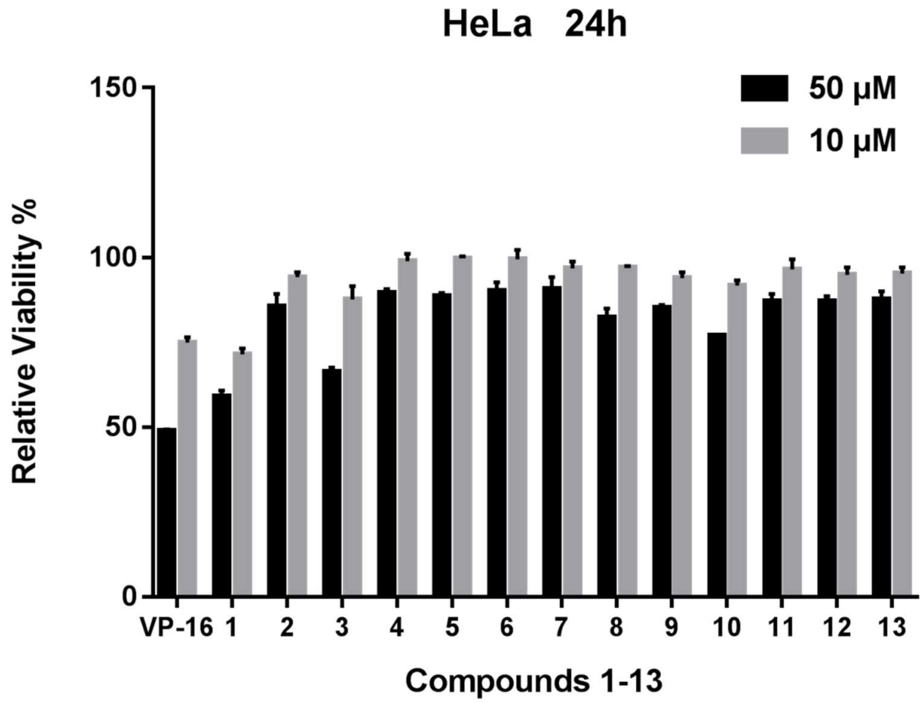

Figure S57. Antiproliferative activity against HeLa cells of compounds **1–13** (50 and 10 μM, respectively). VP-16, a typical antitumor drug that specifically inhibits cell cycle, was used as positive control; DMSO: vehicle control; blank: untreated control without DMSO.

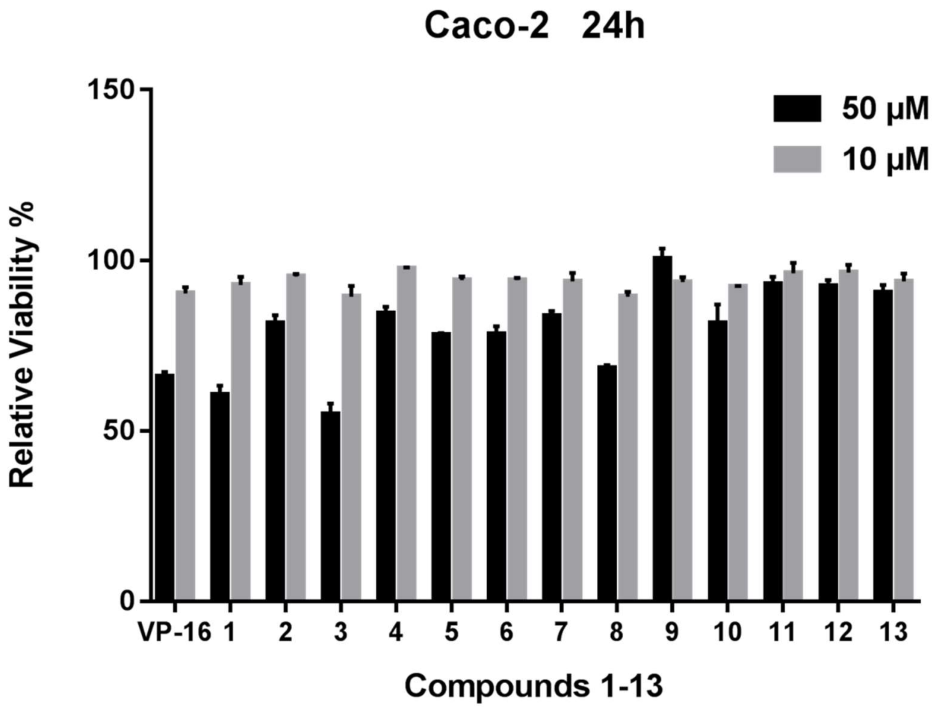

Figure S58. Antiproliferative activity against Caco-2 cells of compounds **1–13** (50 and 10 μM, respectively). VP-16, a typical antitumor drug that specifically inhibits cell cycle, was used as positive control; DMSO: vehicle control; blank: untreated control without DMSO.
